# Genetically encoded 3-aminotyrosine as catalytic residue in a designer Friedel–Crafts alkylase†

**DOI:** 10.1039/d5sc01055a

**Published:** 2025-03-31

**Authors:** Bart Brouwer, Franco Della-Felice, Andy-Mark W. H. Thunnissen, Gerard Roelfes

**Affiliations:** a Stratingh Institute for Chemistry, University of Groningen Nijenborgh 3 9747 AG Groningen The Netherlands j.g.roelfes@rug.nl; b Groningen Biomolecular Sciences and Biotechnology Institute, University of Groningen Nijenborgh 3 9747 AG Groningen The Netherlands

## Abstract

Genetic incorporation of noncanonical amino acids (ncAAs) harbouring catalytic side chains into proteins allows the creation of enzymes able to catalyse reactions that have no equivalent in nature. Here, we present for the first time the use of the ncAA 3-aminotyrosine (aY) as catalytic residue in a designer enzyme for iminium activation catalysis. Incorporation of aY into protein scaffold LmrR gave rise to an artificial Friedel–Crafts (FC) alkylase exhibiting complementary enantioselectivity to a previous FC-alkylase design using *p*-aminophenylalanine as catalytic residue in the same protein. The new FC-alkylase was optimized by directed evolution to afford a quadruple mutant that showed increased activity and excellent enantioselectivity (up to 95% ee). X-ray crystal structures of the parent and evolved designer enzymes suggest that the introduced mutations cause a narrowing of the active site and a reorientation of the catalytic –NH_2_ group. Furthermore, the evolved FC-alkylase was applied in whole-cell catalysis, facilitated by the straightforward incorporation of aY. Our work demonstrates that aY is a valuable addition to the biochemists toolbox for creating artificial enzymes.

## Abstract 2

An evolved designer enzyme featuring genetically encoded 3-aminotyrosine (aY) as catalytic residue promotes highly enantioselective Friedel–Crafts alkylations, is enantiocomplementary to a prior design, and enables whole-cell biocatalysis.

## Graphical abstract

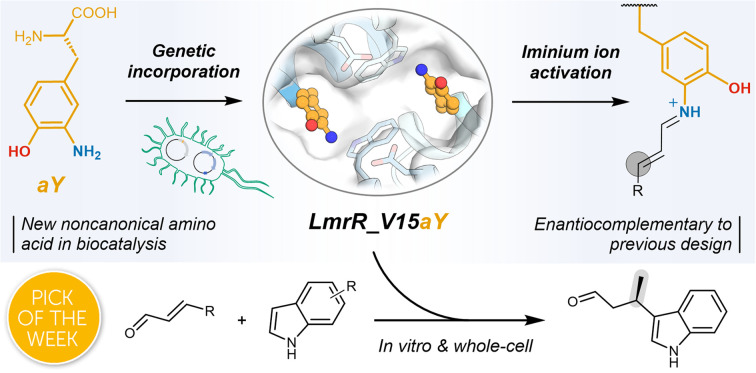

## Introduction

The efficiency and selectivity of enzymes under mild reaction conditions are attractive characteristics for their application in the chemical industry.^1–3^ However, for many reactions that are routinely used by chemists, there are no natural enzymes available. The biocatalytic repertoire of enzymes can be expanded by the development of designer enzymes harbouring chemical functionalities that are not generally observed in nature.^4–8^ Stop codon suppression (SCS) has emerged as a powerful strategy to create such enzymes as it allows for site-specific incorporation of a noncanonical amino acid (ncAA) into a protein scaffold at any desired position.^9–11^ The incorporation of ncAAs with side chains containing organocatalytic groups is particularly promising, as it allows for straightforward production of rudimentary enzymes with novel catalytic activities that can subsequently be optimized by directed evolution.^11,12^ NcAAs that have been used as organocatalytic residues in designer enzymes include: *p*-aminophenylalanine (pAF),^13–20^ N_δ_-methyl- and N_δ_-vinylhistidine,^21–23^ pyrrolysine derivatives harbouring a secondary amine,^24–27^ and, recently, *p*-boronophenylalanine (pBoF).^28–30^

**Scheme 1:**
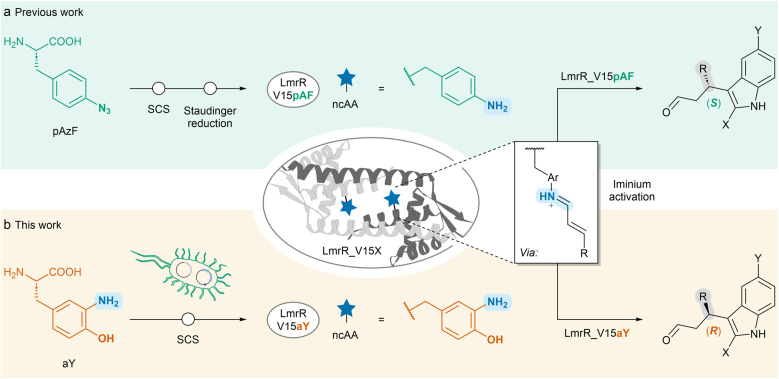
Creation of LmrR-based designer enzymes by genetic incorporation of ncAAs *via* SCS and their application in the vinylogous FC-alkylation reaction between α,β-unsaturated aldehydes and indoles *via* iminium activation. (a) Previous work incorporating pAzF at position V15, giving LmrR_V15pAF after post-translational Staudinger reduction that yields the (*S*)-enantiomer of the FC-alkylation product.^16^ (b) This work incorporating aY at position V15 directly, giving LmrR_V15aY that yields the (*R*)-enantiomer of the FC-alkylation product.

In previous work, we have introduced pAF into lactococcal multidrug resistance regulator (LmrR), a small homodimeric protein harbouring a pore that exhibits promiscuous substrate binding capabilities,^31,32^ either by direct incorporation using the dedicated orthogonal translation system (OTS),^19^ or, preferably, by introduction of *p*-azidophenylalanine (pAzF), followed by a post-translational Staudinger reduction.^13–18,20^ The designer enzymes thus created were successfully employed in iminium ion catalysis, including hydrazone formation and conjugate addition reactions, such as the vinylogous Friedel–Crafts (FC) alkylation (Scheme 1a). Encouraged by these results, we decided to further explore the nature of the catalytic moiety, as substitutions on aniline catalysts can impact their catalytic behavior.^33^ For this purpose, we decided to explore the application of the ncAA 3-aminotyrosine (aY) as catalytic residue (Scheme 1b). SCS has been used to incorporate aY as a mechanistic probe to study the role of redox active tyrosines in ribonucleotide reductase.^34–37^ It has also been employed to investigate outer-sphere interactions within the distal pocket of myoglobin, resulting in improved peroxidase activity in an engineered variant.^38^ We envisioned that aY could also serve as catalytic residue for iminium catalysis.

Here, we show that incorporation of aY into LmrR gives rise to a designer FC-alkylase that exhibits enantiocomplementarity to our previous design based on pAF (Scheme 1b), demonstrating that stereoselectivity can be controlled by the choice of catalytic residue. Directed evolution of the newly created designer enzyme gave rise to a highly selective FC-alkylase, reaching up to 95% ee. Moreover, the efficient and straightforward incorporation of aY into the protein, combined with the high activity and selectivity achieved in catalysis, allowed the use of the evolved designer enzyme in whole-cell biocatalysis.

## Results and discussion

### aY as catalytic residue

Whereas the first developed OTS for the genetic incorporation of aY had issues with incorporation efficiency, Zhang and Ai reported the evolution of the corresponding *Methanococcus jannaschii* tyrosyl-derived tRNA synthetase, resulting in a more efficient OTS with better fidelity towards aY.^39^ We used this improved OTS to incorporate aY *via* amber SCS at position V15 in the LmrR protein. Position V15 resides inside the pore of LmrR, and is the same position of ncAA incorporation as used in earlier works featuring pAF as catalytic residue.^13–20^ By adding the ncAA as a solid to the growth medium, *E. coli* was able to efficiently incorporate aY into LmrR, giving high yields (up to 170 mg L^−1^ cell culture) of dimeric LmrR_V15aY (further referred to as V15aY) after purification by affinity chromatography, as shown by SDS-PAGE and characterization by size exclusion chromatography (Fig. S1 and S2†). HRMS analysis of the purified protein confirmed the incorporation of aY (Fig. S3†).

With V15aY in hand, its activity in iminium catalysis was evaluated in the vinylogous FC-alkylation reaction between crotonaldehyde (1a) and 2-methylindole (2a), forming product 3a after *in situ* reduction with sodium borohydride for analytical purposes. Strikingly, the opposite enantiomer of product 3a was obtained when compared to LmrR_V15pAF (further referred to as V15pAF), albeit with low yield (Table 1, entries 2 and 3). It is remarkable that by solely changing the catalytic ncAA from pAF to aY, and with it the position of the –NH_2_ functionality from *para* to *meta*, the enantioselectivity towards this FC-alkylation was inverted. Optimization of the reaction conditions (60 μM catalyst, MES buffer pH 5.5) improved the performance to 10% yield and 67% ee (Table 1, entries 4–6 and Fig. S4†). To investigate the effect of the microenvironment of the LmrR pocket on the catalytic potential of V15aY, we decided to transplant two sets of active site mutations that were found in previous evolution campaigns of V15pAF. Variants V15aY_RMH, featuring mutations A92R_N19M_F93H found in the evolution of V15pAF towards the hydrazone formation,^14^ and V15aY_RGN, featuring mutations L18R_S95G_M89N found in the evolution of V15pAF as FC-alkylase,^16^ were produced and tested in the FC-alkylation reaction. Compared to V15aY, a two- and four-fold increase in yield was observed for V15aY_RMH and V15aY_RGN, respectively (Table 1, entries 7 and 8). However, the increase in activity was accompanied by a decrease in enantioselectivity, particularly for V15aY_RMH. These results illustrate the importance of the protein microenvironment around the catalytic ncAA. Controls with variants harbouring tyrosine instead of aY at V15 resulted in minimal yields and enantioselectivities (Table 1, entries 9 and 10), confirming the involvement of aY as catalytic residue.

**Table 1:** Vinylogous FC-alkylation reaction between crotonaldehyde (1a) and 2-methylindole (2a) catalyzed by LmrR variants^a^

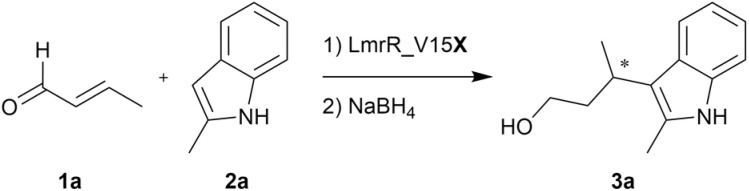					
Entry	Catalyst	Catalyst conc. (μM)	pH	Yield^b^ (%)	ee^c^ (%)
1	—	—	6.5	<1	n.d.
2	V15aY	20	6.5	3 ± 0	39 ± 2
3	V15pAF	20	6.5	53 ± 2	−39 ± 2^d^
4	V15aY	60	6.5	6 ± 0	54 ± 1
5	—	—	5.5	<1	n.d.
6	V15aY	60	5.5	10 ± 1	67 ± 1
7	V15aY_RMH	60	5.5	23 ± 1	11 ± 0
8	V15aY_RGN	60	5.5	39 ± 2	51 ± 2
9^e^	V15Y_RMH	60	5.5	4 ± 0	2 ± 0
10^e^	V15Y_RGN	60	5.5	2 ± 0	<1
11^e^	V15aY_R	60	5.5	16 ± 0	60 ± 0
12^e^	V15aY_G	60	5.5	9 ± 0	57 ± 0
13^e^	V15aY_N	60	5.5	13 ± 0	67 ± 1
14^e^	V15aY_RG	60	5.5	18 ± 0	59 ± 0
15	V15aY_RN	60	5.5	47 ± 2	71 ± 0
16^e^	V15aY_GN	60	5.5	15 ± 0	36 ± 0

a Reaction conditions: LmrR_V15X variants (20 or 60 μM dimer concentration), 1a (5 mM), 2a (1 mM) in phosphate buffer (50 mM, pH 6.5) or MES buffer (20 mM, pH 5.5), containing NaCl (150 mM) and DMF (8% v/v) in a total volume of 300 μL, continuously inverted for 16 h at 8 °C. Reduction was performed with NaBH_4_ (60 μL, 20 mg mL^−1^ in 0.5% w/v NaOH) to yield alcohol 3a. Reaction extracts were analysed by HPLC or SFC. Unless otherwise specified, entries are based on at least three experiments, using two or more independently produced batches of protein. Errors are the standard deviation of the results.

b Analytical yields determined based on a calibration curve of 3a, using 1*H*-indole-3-propanol as internal standard.

c ee determined by HPLC or SFC.

d ee is assigned relative to the enantiomer obtained with V15aY, in which the “–” symbol represents the opposite enantiomer.

e Results are the average of technical duplicates. n.d. = not determined.

### Directed evolution

**Fig. 1:**
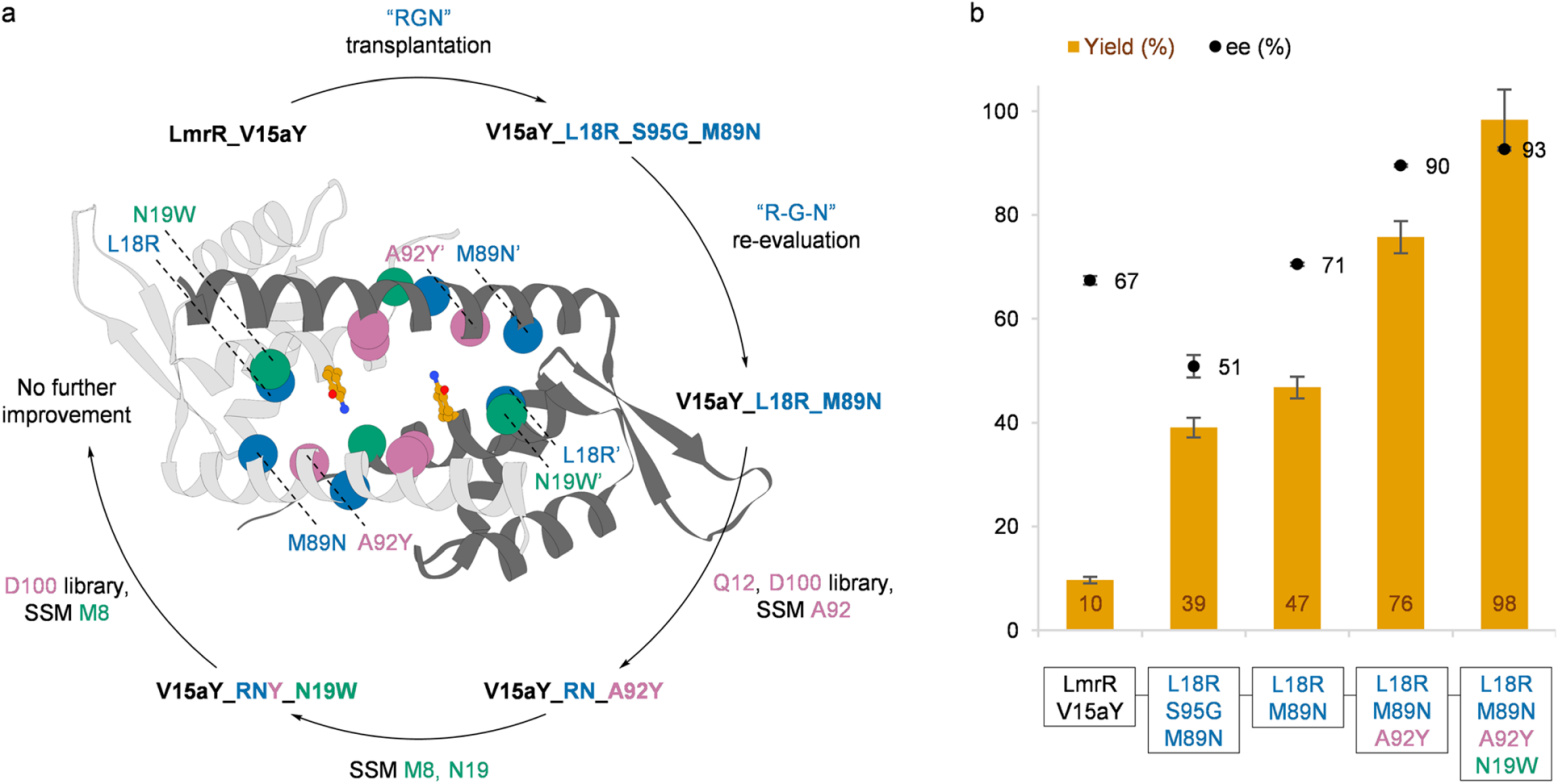
Directed evolution of V15aY for the enantioselective vinylogous FC-alkylation of 2a with 1a. (a) Trajectory of the directed evolution campaign. Positions targeted in each round are displayed as correspondingly coloured spheres in the crystal structure of LmrR_V15aY_RNYW (PDB 9H88), with the aY side chain displayed in orange. (b) Analytical yield and ee of the FC-alkylation reaction between 1a and 2a to give 3a for the different variants obtained during directed evolution, using 60 μM protein at pH 5.5 (see Table 1 for reaction conditions). Results are based on at least three experiments, using two or more independently produced batches of protein. Errors are the standard deviation of the results.

We sought to further improve the efficiency of the newly created aY-based designer enzyme *via* directed evolution. Choosing V15aY_RGN as our starting point for optimization (Fig. 1a), we first re-evaluated the L18R, S95G and M89N mutations, testing all the possible single and double mutants (Table 1, entries 11–16). The single mutations, V15aY_R/G/N, did not lead to a major increase in performance compared to V15aY. In fact, V15aY_G performed worse than the parent enzyme. Double mutants V15aY_RG and V15aY_GN gave a small increase in yield, but with a decreased ee. Interestingly, the combination of L18R and M89N showed an epistatic effect, giving rise to a 4.7-fold increase in yield compared to V15aY, which is higher than what was obtained with the triple mutant V15aY_RGN (Fig. 1b). As also observed from the single and double mutants containing S95G, it appears that this mutation is not beneficial to the activity of V15aY.

Using V15aY_RN as a parent, an alanine/leucine scan of twelve positions in proximity of the aY residue within the hydrophobic pocket of LmrR was performed to identify target residues for further mutagenesis. Most of the mutations had a significant negative effect on catalysis (Fig. S5†), indicating the functional or structural importance of the residues at these positions. Mutants Q12A, N19A, A92L, W96A and D100A gave rise to the largest declines in activity and/or enantioselectivity. Interestingly, W96A reverted the enantioselectivity, albeit at a significant loss in yield. Positions Q12, D100 and A92 were selected for mutagenesis (Fig. 1a). As observed in crystal structures of LmrR,^14,31^ Q12 forms a hydrogen bond with S95′ (the apostrophe denotes that the residue resides in the dimer related subunit) and potentially also plays a role in positioning W96′ (Fig. S6†). We prepared and tested a small, focused Q12 library consisting of enzymes with one of the following mutations at this position: N; E; K; H; L; S. All these mutations had a significant negative effect on the ee (Table S1†), suggesting that the specific size and polar nature of Q12 are important for enantioselectivity. Since D100A showed a significant decrease in both yield and ee, the same approach was followed for D100, which is located close to the surface at the centre of the dimeric interface (Fig. S6†). A focused D100 library (E; N; Q; R; L; H) was therefore also tested. An acidic residue at position D100 appeared to be important as all mutants except D100E exhibited severely diminished activity and enantioselectivity (Table S1†). Next, a site-saturation mutagenesis (SSM) library was prepared for position A92. This SSM library was screened for FC-alkylation activity in cell lysates obtained from cultures grown in 24-deep-well plates (Fig. S7 and S8†). Placing large aromatic residues, and in particular tyrosine, at position A92 lead to significantly improved yields and activities compared to the V15aY_RN parent. These results were confirmed using purified V15aY_RN_A92Y (V15aY_RNY), which gave 76% yield and 90% ee (Fig. 1b and Table S1†). In a following round of evolution, SSM libraries of two additional positions in the neighbourhood of the ncAA, *i.e.* M8 and N19, were prepared using V15aY_RNY as template (Fig. 1a and S7, S8†). Several variants from both SSM libraries performed better than the parent, of which mutant V15aY_RNY_N19W (RNYW) showed the largest improvements, giving 98% yield and 93% ee when used as purified protein (Fig. 1b and Table S1†). Subsequent screening of the focused D100 library and SSM at position M8, now using V15aY_RNYW as parent, did not lead to any further improvement (Fig. 1a, S7, S8 and Table S1†).

Good yields and high enantioselectivities could also be obtained with our final evolved mutant, V15aY_RNYW, when using lower catalyst loading (3 mol%), shorter reaction times (3 or 6 h) and higher reaction temperature (25 °C) (Table S2†). Controls using V15Y_RNYW or denatured V15aY_RNYW did not show enantioselective catalysis (Table S3†). Interestingly, the beneficial effects of the RNYW mutations were found to not be transferable to V15pAF as transplanting the RNYW mutations into V15pAF did not lead to any significant catalytic improvement (Table S3†).

### Kinetic and structural characterization

The catalytic performance of V15aY and the evolved mutant V15aY_RNYW were assessed by measuring initial reaction rates at varying concentrations of substrate 2a. Kinetic parameters *k*_cat, app_ and *K*_M-__2a__, app_ were determined according to the Michaelis–Menten equation (Fig. S9†). The results showed that directed evolution led to a 3.4-fold increase in apparent catalytic efficiency, with (*k*_cat_/*K*_M-__2a_)_app_ values of 3.3 M^−1^ s^−1^ and 11.2 M^−1^ s^−1^ for V15aY and V15aY_RNYW, respectively. While the introduced mutations did not have a significant effect on the *K*_M-__2a__, app_ of the enzyme, they resulted in a substantial increase in *k*_cat, app_, from 0.24 min^−1^ for V15aY to 0.76 min^−1^ for V15aY_RNYW. Hence, the observed improvement of catalytic efficiency for the evolved mutant is exclusively due to an improvement in *k*_cat_.

**Fig. 2:**
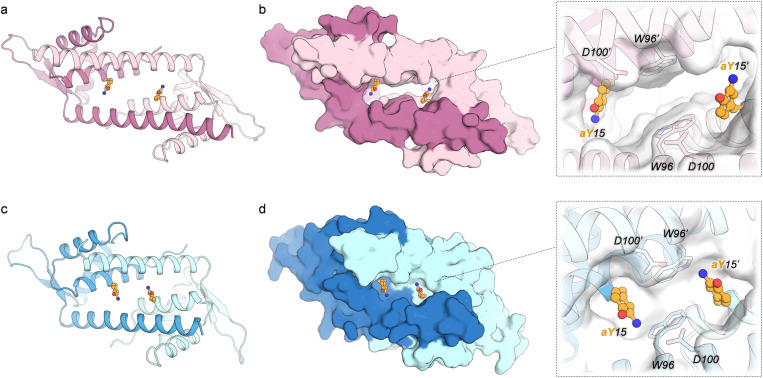
Crystal structures of LmrR_V15aY (PDB 9H87, depicted in pink, panel a and b) and LmrR_V15aY_RNYW_KK (PDB 9H88, depicted in blue, panel c and d), displayed as cartoon representations (a and c), or surface representations showing zoomed-in views of the dimeric interfaces (b and d). The two chains of the LmrR dimer are shown in different colour shades and the aY residues at positions 15 and 15′ (aY15 and aY15′) are shown as orange ball and sticks.

**Fig. 3:**
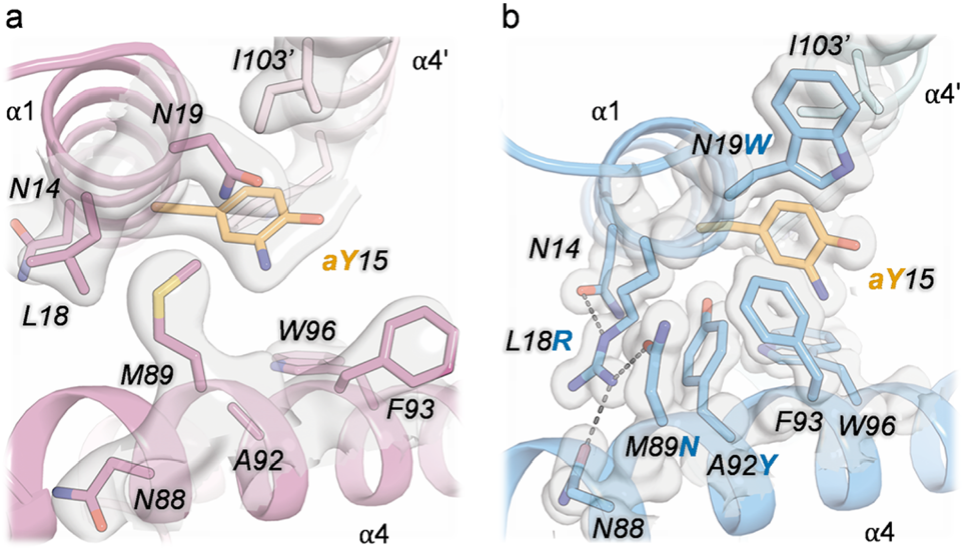
Close-up views of the crystal structures of LmrR_V15aY (a) and LmrR_V15aY_RNYW_KK (b), highlighting 2Fo-Fc electron densities for selected residues (contoured at 1σ). Hydrogen bonds are displayed as dotted black lines. The mutations L18R, M89N, A92Y and N19W in the evolved variant result in an increased close packing of side chains of helices α1 and α4, achieved *via* the formation of hydrophobic contacts, aromatic stacking interactions and hydrogens bonds.

To investigate the structural consequences of the RNYW mutations, the crystal structures of V15aY (PDB 9H87) and V15aY_RNYW (PDB 9H88) were solved at a resolution of 2.15 Å and 1.20 Å, respectively (Fig. 2 and Table S4†). For the latter, residues originally involved in DNA-binding of LmrR, K55 and K59 (KK),^40^ were reinstated to facilitate the formation of crystals with sufficient X-ray diffracting quality. Reintroduction of the KK-mutations had minimal impact on catalysis (Table S3†). Remarkably, while V15aY exhibits the characteristic LmrR structure, forming an open pore at its dimeric interface of similar shape and dimensions as observed in other LmrR structures,^14,31^ the V15aY_RNYW_KK structure appears to be more closed and exhibits a narrowed cavity. Furthermore, a significant difference in the rotameric state of the aY side chain is observed (Fig. 2b, d and S10, S11†). In the parent structure, the –NH_2_ group of aY is oriented towards the side of the pore, away from the central W96 and W96′ residues. In contrast, in the evolved variant it is placed closer to the pore centre, directed more towards the central tryptophans. The narrowing of the dimeric interface in the evolved variant can be directly linked to the RNYW mutations, which enhance side chain packing between the α1 and α4 helices (Fig. 3). The enhanced packing is stabilized through a combination of hydrophobic interactions, aromatic stacking, and hydrogen bonds. Notably, L18R forms hydrogen bonds with M89N, N88 and N14 in the evolved variant, which may explain the epistatic effect observed in catalysis when the L18R and M89N mutations were combined. Interestingly, the same hydrogen-bonds have previously been observed in the crystal structure of LmrR_V15pAF_RGN, which also exhibits a smaller pore size compared to its parent.^41^ Hence, this suggests that the RN mutations are key to forming the observed narrowed dimeric interfaces that appear to be beneficial in FC-alkylations, while the introduced Y and W residues most likely further fine-tune the structure and the interactions responsible for achieving efficient and enantioselective catalysis. Moreover, the close proximity of D100 to the aY residue (Fig. 2b and d) suggests a potential interaction between the carboxylic residue and the substrates, similar to what has been proposed for V15pAF_RGN.^41^ This is in agreement with the observed detrimental effect on catalysis of mutation of D100 during the evolution campaign. However, an in-depth mechanistic and structural study will be required to identify the exact roles of the introduced mutations and the residues in the vicinity of the catalytic residue.

### Substrate scope

**Scheme 2:**
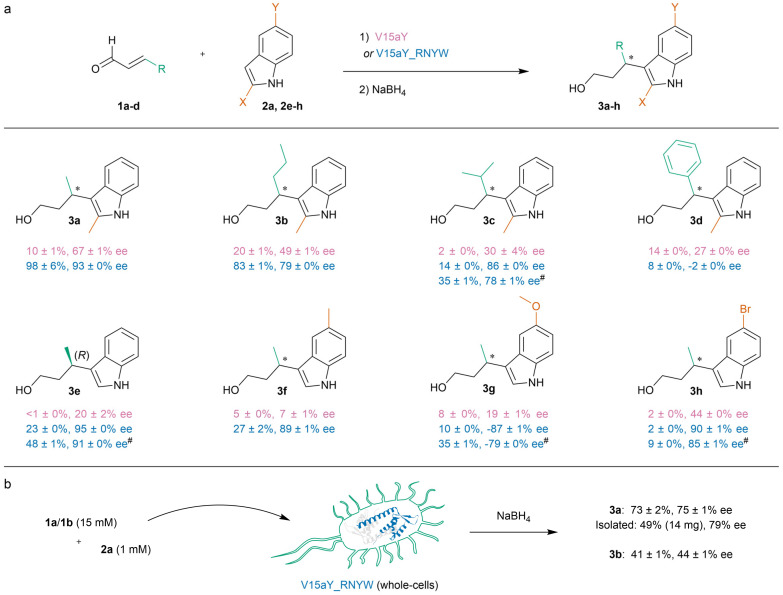
(a) Substrate scope of the enantioselective vinylogous FC-alkylation reaction catalysed by V15aY and V15aY_RNYW. Reaction conditions (unless otherwise noted): V15aY variant (60 μM dimer concentration), 1a–d (5 mM), 2a, 2e–h (1 mM) in MES buffer (20 mM, pH 5.5), containing NaCl (150 mM) and DMF (8% v/v) in a total volume of 300 μL, continuously inverted for 16 h at 8 °C. For 3a–d and 3f–h, ee is assigned relative to the enantiomer obtained with V15aY, in which the “–” symbol represents the opposite enantiomer. For 3e, absolute configuration is assigned by comparison of order of elution on HPLC with the literature and previous work.^16,42^^#^Performed using 25 mM 1, see Table S5† for results of reactions performed at 25 °C. (b) Whole-cell catalysis and application on millimolar scale. Reaction conditions: washed whole-cells expressing V15aY_RNYW (6 OD_600_ units), 1a/1b (15 mM), 2a (1 mM) in MES buffer (20 mM, pH 5.5), containing NaCl (150 mM) and DMSO (8% v/v) in a total volume of 300 μL, continuously inverted for 21 h at 8 °C. Results are based on at least three experiments, using two or more independently produced batches of protein, or three independent cultures of whole-cells. Errors are the standard deviation of the results. See ESI† sections 9 and 11 for more details.

The substrate scope of V15aY and V15aY_RNYW was explored by testing several β-substituted-α,β-unsaturated aldehydes and indoles (Scheme 2a). V15aY_RNYW outperformed V15aY in nearly all examples, achieving high enantioselectivities (up to 95% ee). A longer alkyl substituent on the α,β-unsaturated aldehyde was well tolerated (3b). However, bulkier isopropyl and phenyl substituents lead to significantly lower yields (3c, 3d). Interestingly, when using cinnamaldehyde (3d), parent V15aY exhibited both higher yield and ee compared to V15aY_RNYW, indicating divergent substrate selectivities for the different variants that could be potential targets for further optimization. Moreover, V15aY gave a higher yield and ee than obtained previously with V15pAF in the same reaction.^16^ Using unsubstituted indole, or 5-substituted indole substrates (3e–h), also resulted in high enantioselectivities, albeit with lower yields. Reaction yields could be improved with minimal loss in enantioselectivity by using 25 mM of aldehyde substrate (Scheme 2a) or by increasing the reaction temperature to 25 °C as demonstrated for selected scope examples (Table S5†). The absolute configuration of product 3e, obtained in excellent enantioselectivity (95%), was determined to be (*R*), whereas V15pAF_RGN produces the (*S*) enantiomer of 3e in excess.^16,42^ V15aY had a small preference for the opposite enantiomer of 3g compared to V15aY_RNYW, once again indicating divergent selectivities for different mutants. With exception of 3d, in all cases the opposite enantiomers of the products were obtained compared to V15pAF_RGN (3a–e).^16^ Hence, V15aY_RNYW and V15pAF_RGN can be considered enantiocomplementary artificial FC-alkylases.

### Whole-cell catalysis

The straightforward incorporation of aY into proteins, without the need for any post-translational modification, is an appealing feature that suggests the potential of aY-based enzymes in whole-cell or *in vivo* applications, something that was not readably feasible with V15pAF_RGN. Hence, we investigated if we could apply whole *E. coli* cells expressing V15aY_RNYW to perform the FC-alkylation between 1a and 2a without cell lysis or purification step. To our delight, we observed the formation of product 3a in good yields and enantioselectivities under standard reaction conditions, reaching up to 84% yield and 83% ee when using high whole-cell loading (16 OD_600_ units/300 μL) (Table S6†). Using DMSO instead of DMF as co-solvent, and performing the reaction with altered crotonaldehyde concentrations, reaction temperatures and/or reaction times were well tolerated (Table S6†). Controls using whole-cells expressing LmrR_WT showed some background reactivity, but without enantioselectivity. Following centrifugation after catalysis, most of the product was obtained from the supernatant (Table S6†). Encouraged by these results, we envisioned we could apply whole-cells expressing V15aY_RNYW in a preparative scale reaction. To facilitate this, we decided to use moderate whole-cell loading (6 OD_600_ units/300 μL) and DMSO as co-solvent, which on small scale retains high yield and enantioselectivity for 3a, and gives moderate yield and ee for 3b (Scheme 2b). The preparative scale reaction (0.14 mmol) lead to the isolation of product 3a in 49% yield and 79% ee after purification by column chromatography. Overall, these results demonstrate the applicability of aY-based artificial enzymes and lay the foundation for further whole-cell biocatalysis applications.

## Conclusions

Genetic code expansion has provided a new platform for enzyme design, making it possible to genetically encode amino acids harbouring noncanonical side chains and apply them as catalytic residues in chemical transformations not generally observed in nature.^10,11^ In this work, we created and evolved a designer enzyme employing, for the first time, genetically incorporated 3-aminotyrosine as catalytic residue for iminium activation. Our findings revealed that the nature of the ncAA significantly influences the catalysed vinylogous Friedel–Crafts alkylation: a subtle shift of the catalytic amine group from *para* to *meta* position on the aromatic side chain when using aY instead of pAF in the same protein at the same position gives rise to opposite enantioselectivity.^16^ Our aY-based enzyme was evolved to a more active and selective variant containing four additional mutations, achieving high enantioselectivities (up to 95% ee). Hence, combined with LmrR_V15pAF_RGN, this new aY-dependent enzyme forms a set of enantiocomplementary FC-alkylases. Finally, the straightforward incorporation of aY, without the need for any post-translational modification, made it possible to apply our evolved FC-alkylase in a whole-cell fashion. This is an attractive feature that facilitates potential synthetic applications of aY-based artificial enzymes.

## Data availability

Crystallographic data for LmrR_V15aY and LmrR_V15aY_RNYW_KK have been deposited at the Protein Data Bank (PDB) under deposition numbers 9H87 and 9H88, respectively, and can be obtained from https://www.rcsb.org/. The data supporting this article have been uploaded as part of the ESI.†

## Author contributions

B. B. and G. R. conceived the project. B. B. produced, evolved and characterized the designer enzymes, and optimized and performed catalysis experiments. F. D.-F. synthesized and purified reference products and performed the semi-preparative scale reaction. B. B. performed protein crystallizations. A.-M. W. H. T. analysed X-ray data. G. R. supervised the project. All authors discussed the results and B. B. wrote the manuscript. B. B., F. D.-F., A.-M. W. H. T. and G. R. reviewed and edited the manuscript.

## Conflicts of interest

There are no conflicts to declare.

## Supplementary Material

SC-016-D5SC01055A-s001
