# Supplementary material for: Genetically encoded 3-aminotyrosine as catalytic residue in a designer Friedel–Crafts alkylase
Source: Chem Sci. 2025 Mar 31;16(20):8721–8. doi: 10.1039/d5sc01055a (PMC12015630; doi:10.1039/d5sc01055a)
Supplement: SC-016-D5SC01055A-s001 [file SC-016-D5SC01055A-s001.pdf]

Supplementary Information (SI) for Chemical Science.
This journal is © The Royal Society of Chemistry 2025

Supporting Information

Genetically Encoded 3-Aminotyrosine as Catalytic Residue in a Designer Friedel-Crafts Alkylase

Bart Brouwer,[a] Franco Della-Felice,[a] Andy-Mark W.H. Thunnissen,[b] and Gerard Roelfes[a]*

[a]Stratingh Institute for Chemistry, University of Groningen, Nijenborgh 3, 9747 AG Groningen, The Netherlands. [b]Groningen Biomolecular Sciences and Biotechnology Institute, University of Groningen, Nijenborgh 3, 9747 AG Groningen, The Netherlands.

*Corresponding author e-mail address: j.g.roelfes@rug.nl.

Table of Contents

1. Supporting Figures S2
2. Supporting Tables S13
3. General Considerations S18
4. Molecular Biology S19
5. Determination ε_{280} 3-Aminotyrosine S23
6. Protein Production and Purification S24
7. Protein Mass Spectrometry S26
8. Protein Crystallography S34
9. Catalysis and Workup Procedures S35
10. Preparation and Characterization of Reference Products S37
11. Calibration Curves and HPLC / SFC chromatograms S39
12. References S50

1. Supporting Figures

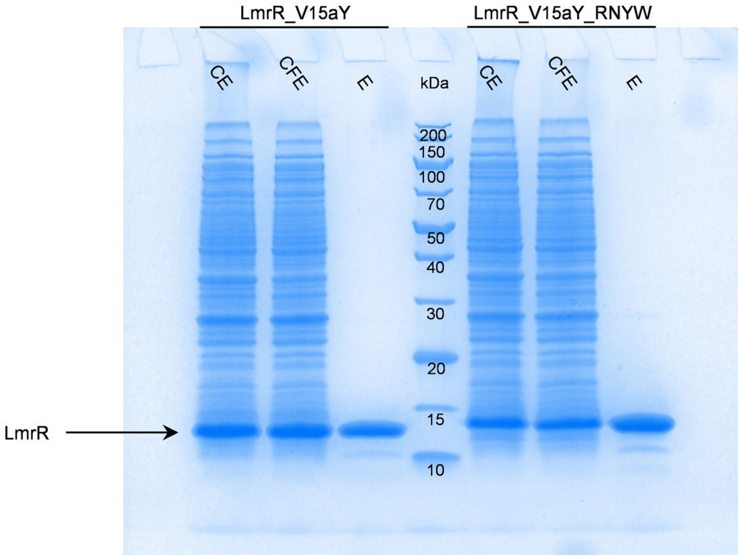

Figure S1. SDS-PAGE (12% acrylamide, Bis-Tris) of LmrR_V15aY and LmrR_V15aY_RNYW. CE = Cell Extract; CFE = Cell-Free Extract; E = Eluted protein, purified by affinity chromatography (Strep-Tactin Superflow high capacity). Ladder = Thermo Scientific PageRuler unstained broad-range protein ladder. For the CE and CFE, ≈ 0.1 OD_{600} units were loaded per well. For the purified protein, 7 µL of a sample containing 15 µM LmrR variant (dimer concentration) was loaded.

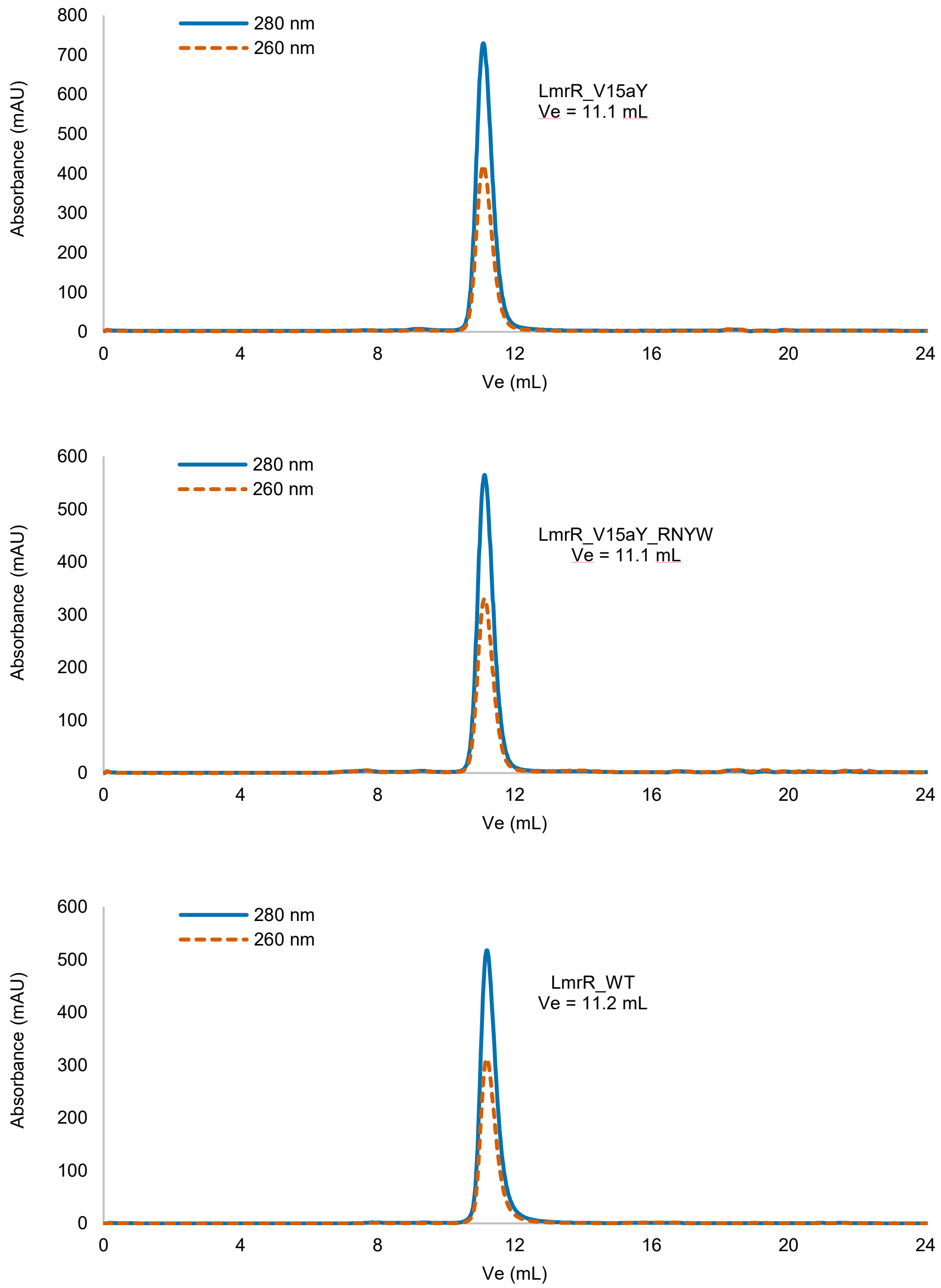

Figure S2. Analytical size exclusion chromatography of purified LmrR_V15aY, LmrR_V15aY_RNYW and LmrR_WT. Ve = elution volume. The observed elution volumes of V15aY and V15aY_RNYW correspond well to the expected homodimeric state (≈ 30 kDa), and are consistent with the observed elution volume of LmrR_WT. Size exclusion chromatography was performed using a Superdex 75 increase 10/300 GL column (Cytiva) mounted on an Äkta Purifier.

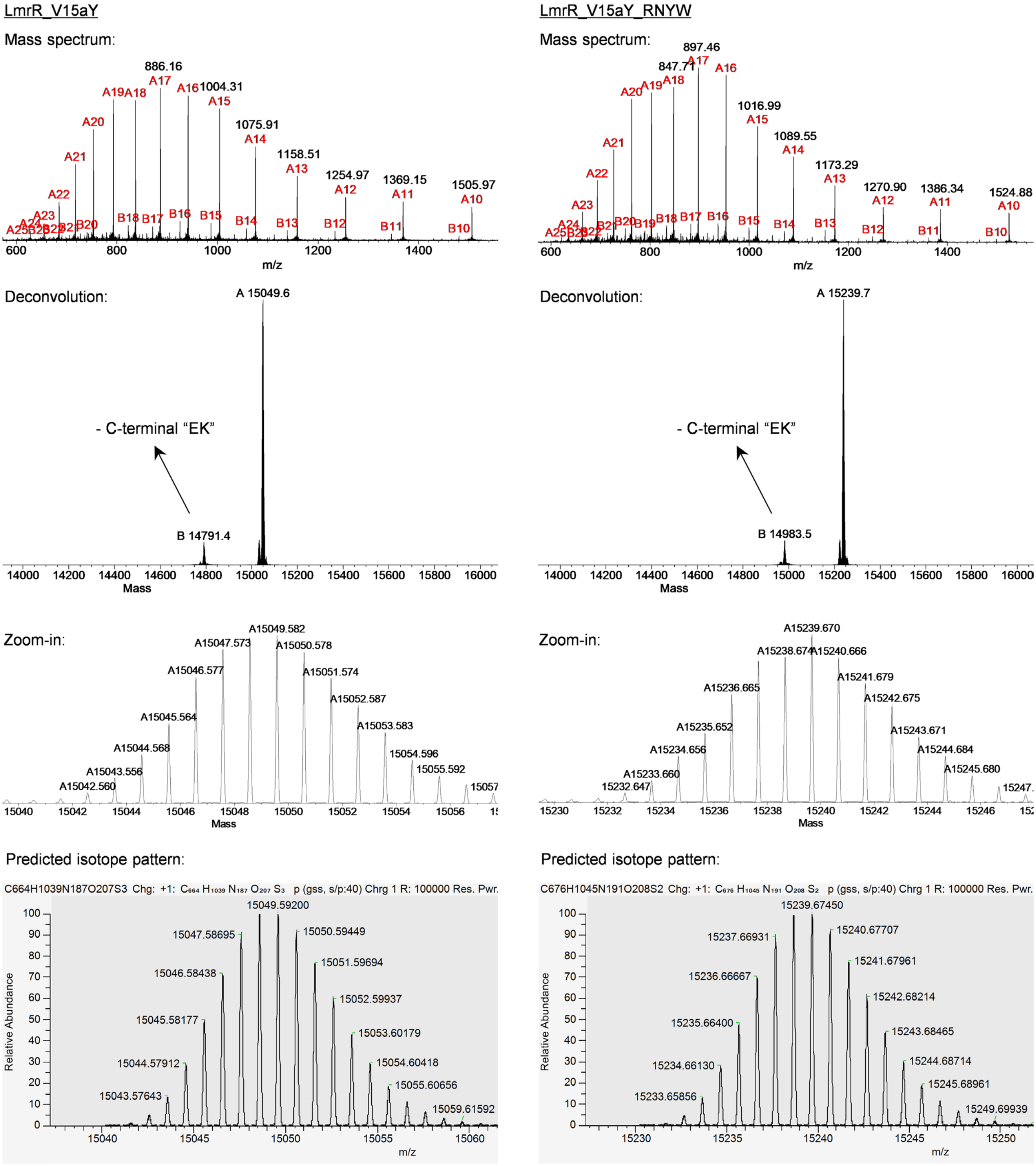

Figure S3. Protein HRMS (Orbitrap Exploris 480) of purified LmrR_V15aY (left) and LmrR_V15aY_RNYW (right). Major species correspond to the expected masses of V15aY and V15aY_RNYW. Minor species correspond to cleavage of two C-terminal StrepTag residues (EK). This cleavage is however not expected to have an effect on structural or functional integrity of the protein. Zoomed-in views of the major species show that the isotope patterns correspond well to the predicted isotope patterns for V15aY and V15aY_RNYW, confirming efficient incorporation of aY. Deconvolution was performed with MagTran. Predicted Isotope patterns were predicted using Xcalibur Freestyle 1.8 using a profile resolution of 100000. For more details, see ESI section 7.

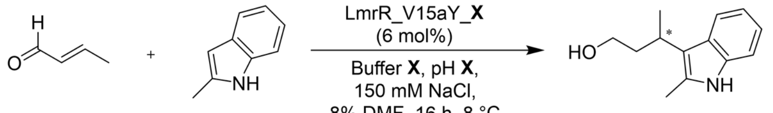

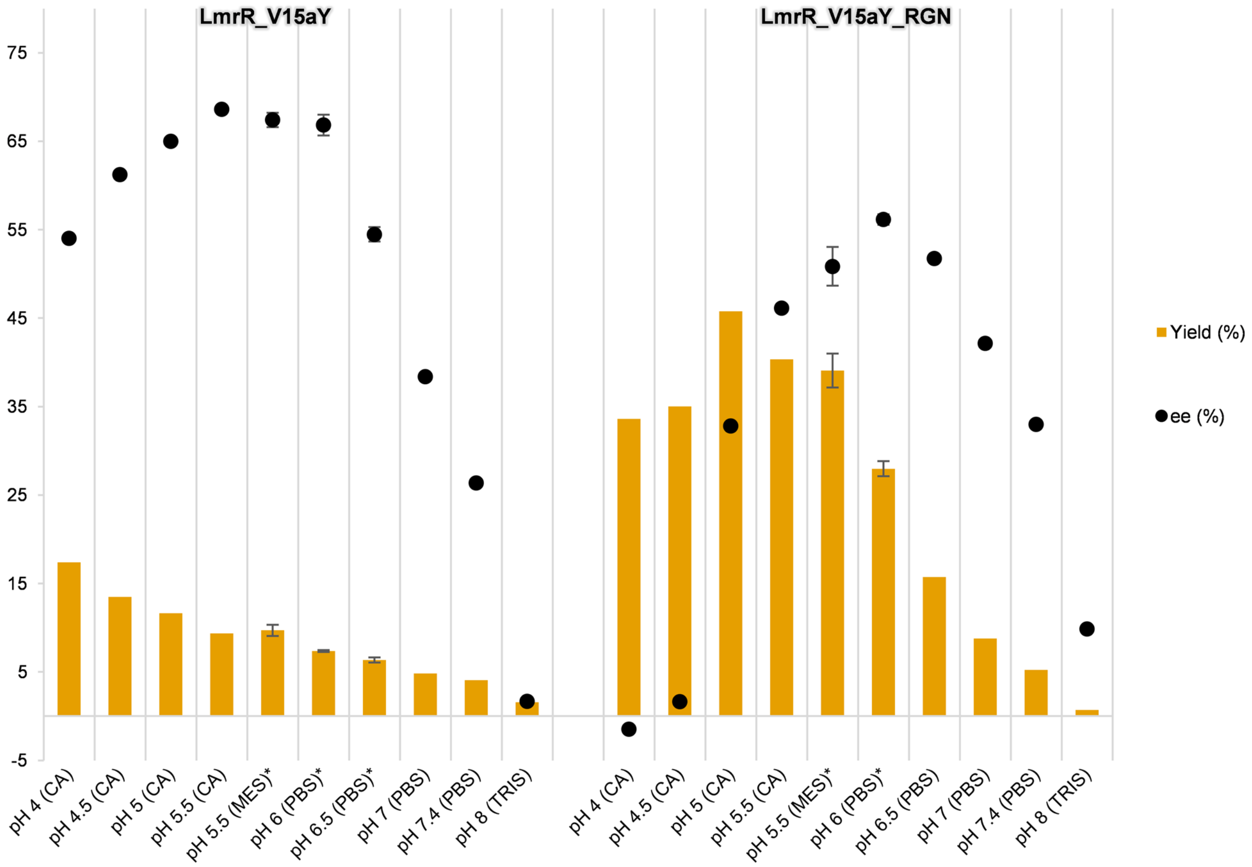

Figure S4. Buffer and pH screening of the V15aY and V15aY_RGN catalysed Friedel-Crafts (FC) alkylation reaction between **1a** and **2a**. A lower pH has a positive effect on yield, whereas there is an optimum for ee around pH 5.5-6. For reactions performed with V15aY_RGN at pH 4.5 or lower, protein precipitation was observed, resulting in a significant loss of ee. This can be attributed to the unfolding of the protein, and thus loss of the chiral environment for catalysis. Results are based on one experiment unless otherwise noted. *Average of at least two experiments, using independently produced batches of protein. Errors are shown as standard deviations. CA = 50 mM citric acid/sodium citrate; MES = 20 mM 2-(N-morpholino)ethanesulfonic acid; PBS = phosphate-buffered saline prepared with 50 mM Na_2HPO_4 and 150 mM NaCl; TRIS = 20 mM tris(hydroxymethyl)aminomethane.

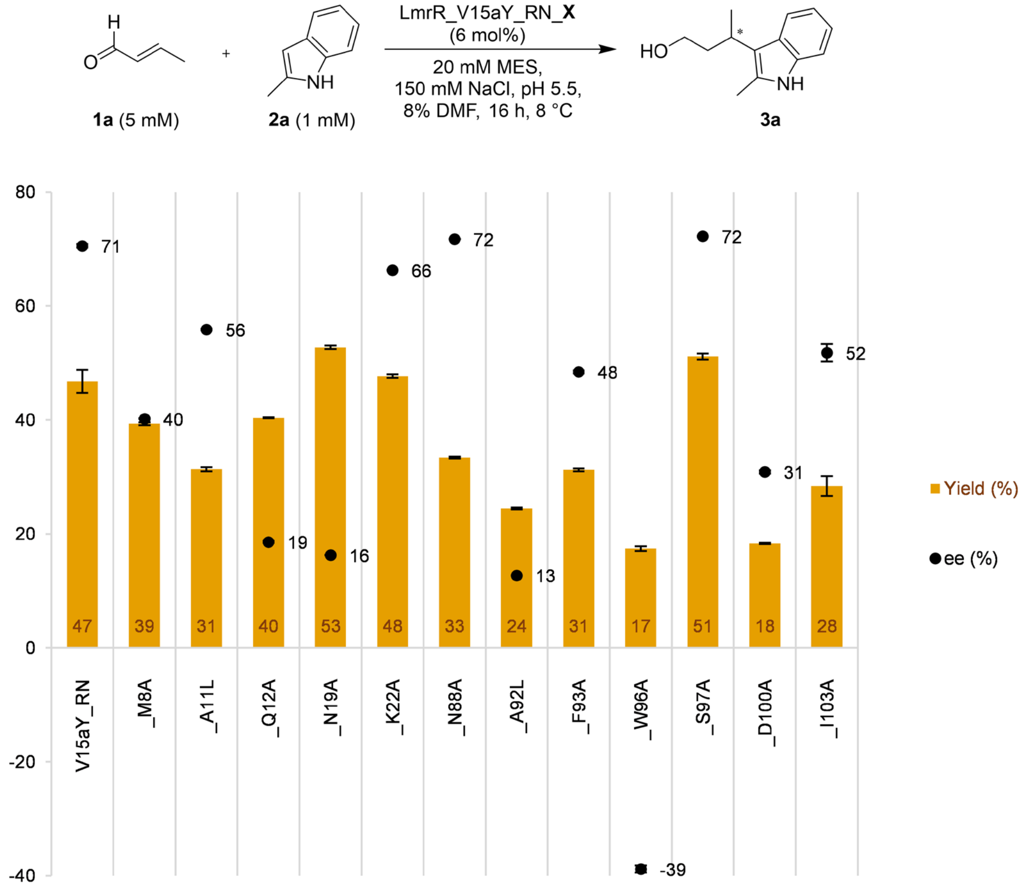

Figure S5. FC-alkylation between **1a** and **2a**, using alanine or leucine mutants of V15aY_RN. Results are an average of at least duplicate experiments. Errors are shown as standard deviations. ee is assigned relative to the enantiomer obtained with V15aY, in which the "–" symbol represents the opposite enantiomer.

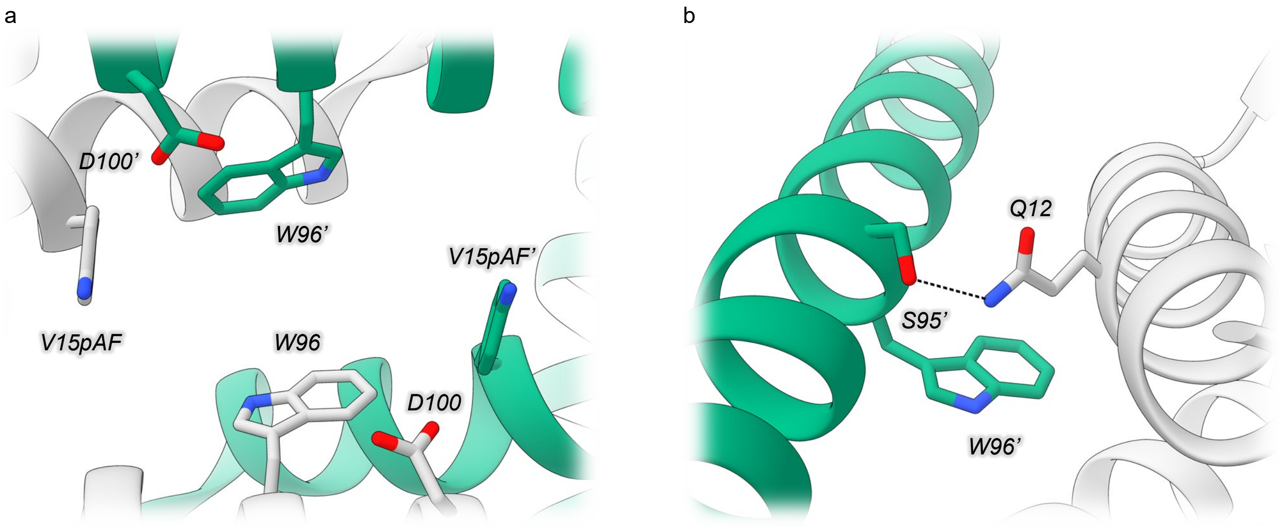

Figure S6. Crystal structure of LmrR_V15pAF (PDB 6I8N)[1]. (a) Close-up view showing the location of D100 at the dimeric interface. The two polypeptide chains of the LmrR dimer are coloured in grey and green (b) Close-up view showing the hydrogen bond between Q12 and S95' and its position relative to W96'.

a

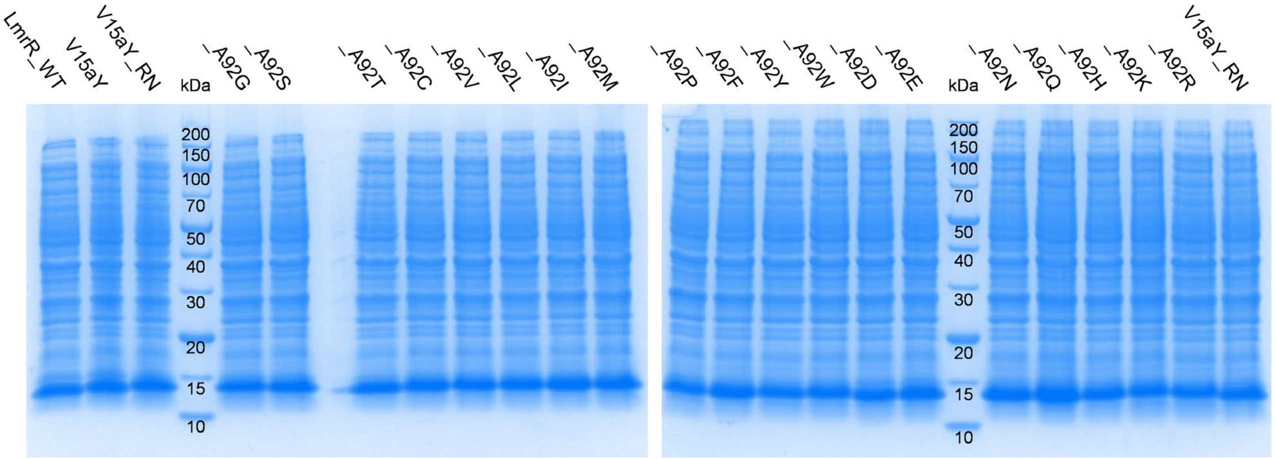
LmrR_WT
V15aY
V15aY_RN
kDa
_A92G
_A92S
_A92T
_A92C
_A92V
_A92L
_A92I
_A92M
_A92P
_A92F
_A92Y
_A92W
_A92D
_A92E
_A92N
_A92Q
_A92H
_A92K
_A92R
V15aY_RN
200
150
100
70
50
40
30
20
15
10

b

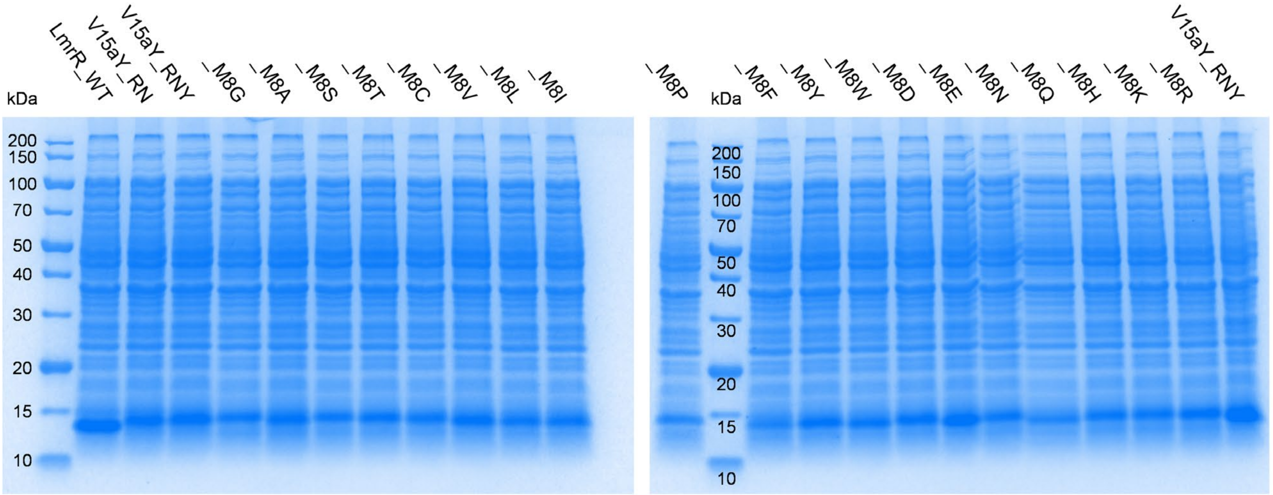
kDa
LmrR_WT
V15aY_RN
V15aY_RNY
_M8G
_M8A
_M8S
_M8T
_M8C
_M8V
_M8L
_M8I
_M8P
_M8F
_M8Y
_M8W
_M8D
_M8E
_M8N
_M8Q
_M8H
_M8K
_M8R
V15aY_RNY
200
150
100
70
50
40
30
20
15
10

c

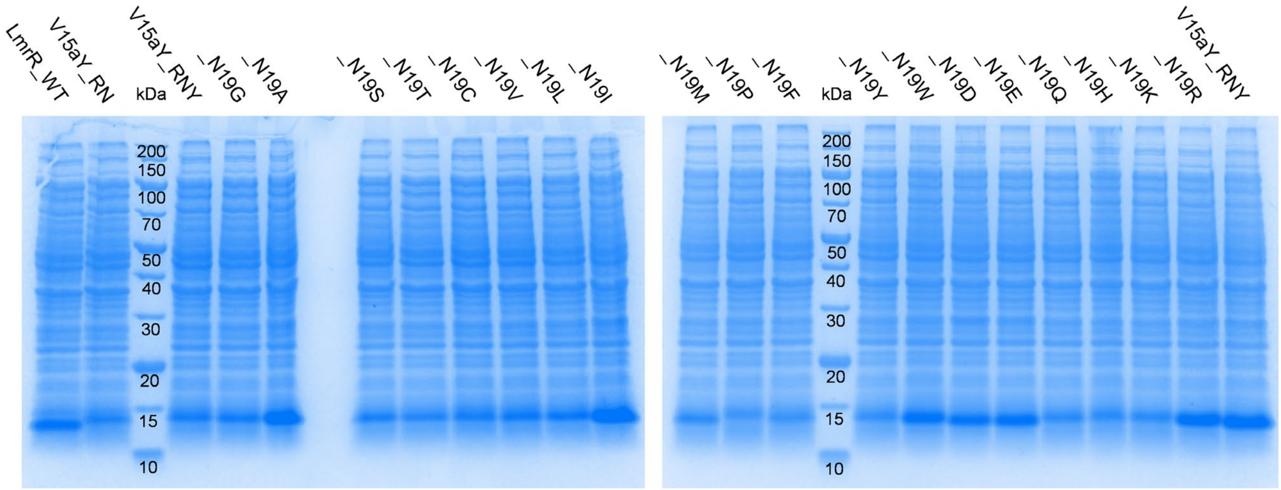
LmrR_WT
V15aY_RN
V15aY_RNY
kDa
_N19G
_N19A
_N19S
_N19T
_N19C
_N19V
_N19L
_N19I
_N19M
_N19P
_N19F
_N19Y
_N19W
_N19D
_N19E
_N19Q
_N19H
_N19K
_N19R
V15aY_RNY
200
150
100
70
50
40
30
20
15
10

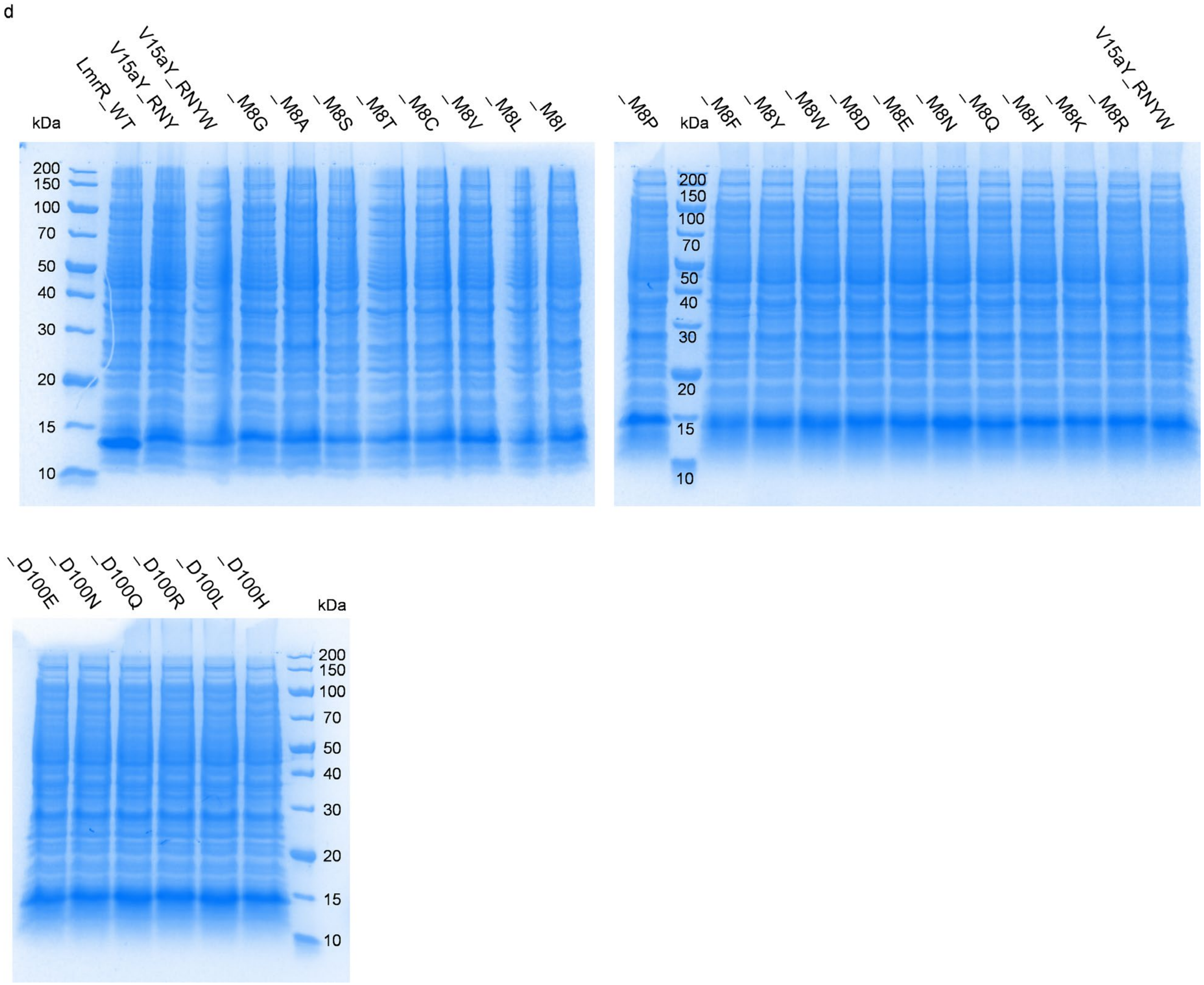

Figure S7. SDS-PAGE of unlysed cells grown in 24-well deep well plates that were subsequently used for FC-alkylation in cell lysates (see **Figure S8**). (a) V15aY_RN_A92 library. (b) V15aY_RNY_M8 library. (c) V15aY_RNY_N19 library. (d) V15aY_RNYW_M8-D100 library. SDS-PAGE samples were prepared using one of the three biological deep well plate replicates per library, see ESI section 6 for experimental details. To prepare SDS-PAGE samples, 50 µL of cells were spun down in a microcentrifuge tube and resuspended in a total volume of 14 µL (Bugbuster primary amine-free (Millipore) + 1X SDS buffer), of which 7 µL (≈ 0.1 OD_{600} units) was loaded per well on freshly made 12% polyacrylamide Bis-Tris gels. The expected mass of each LmrR variant is around 15 kDa.

a - RN_A92 site saturation (276 µL CFE)

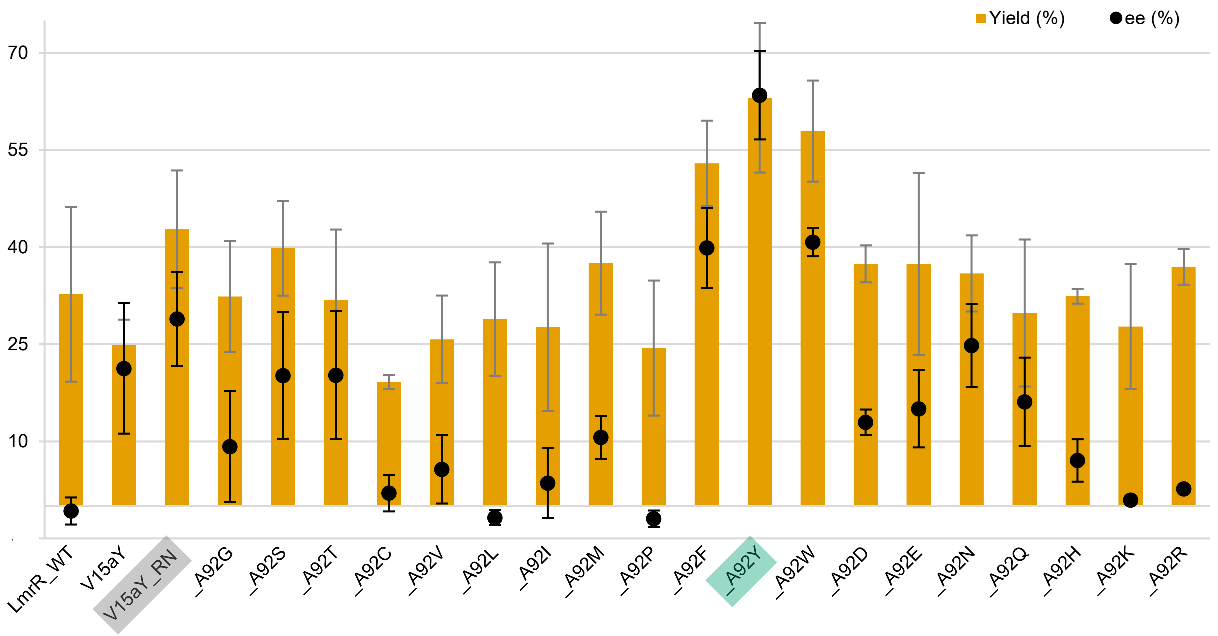

b - RNY_M8 site saturation (180 µL CFE)

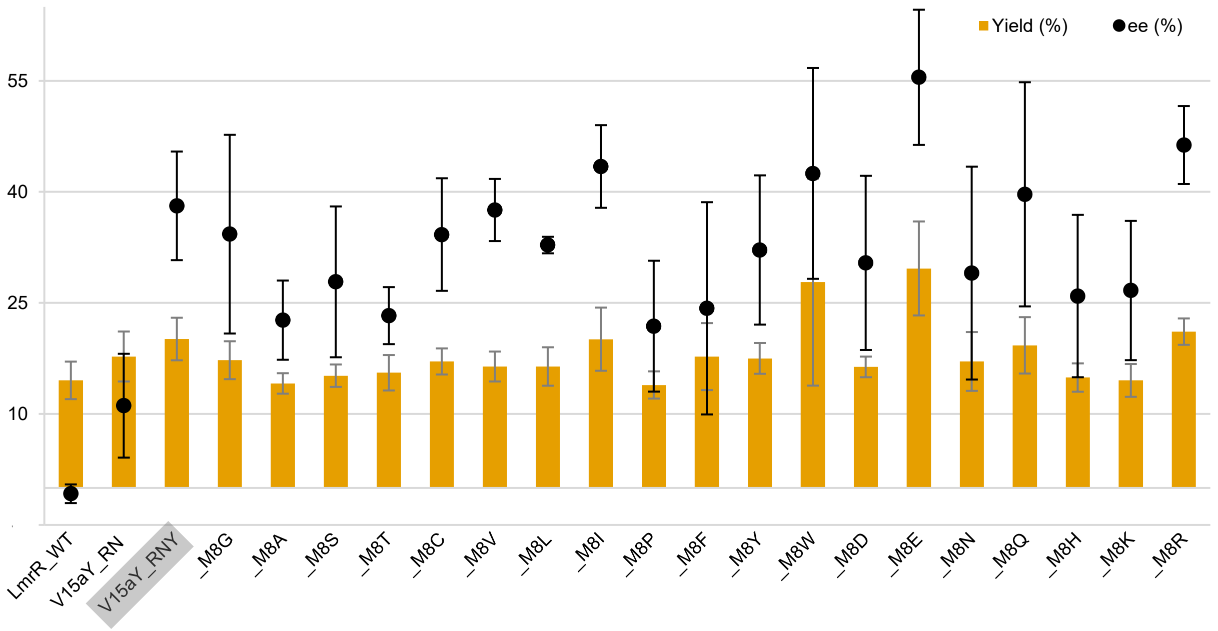

c - RNY_N19 site saturation (276 µL CFE)

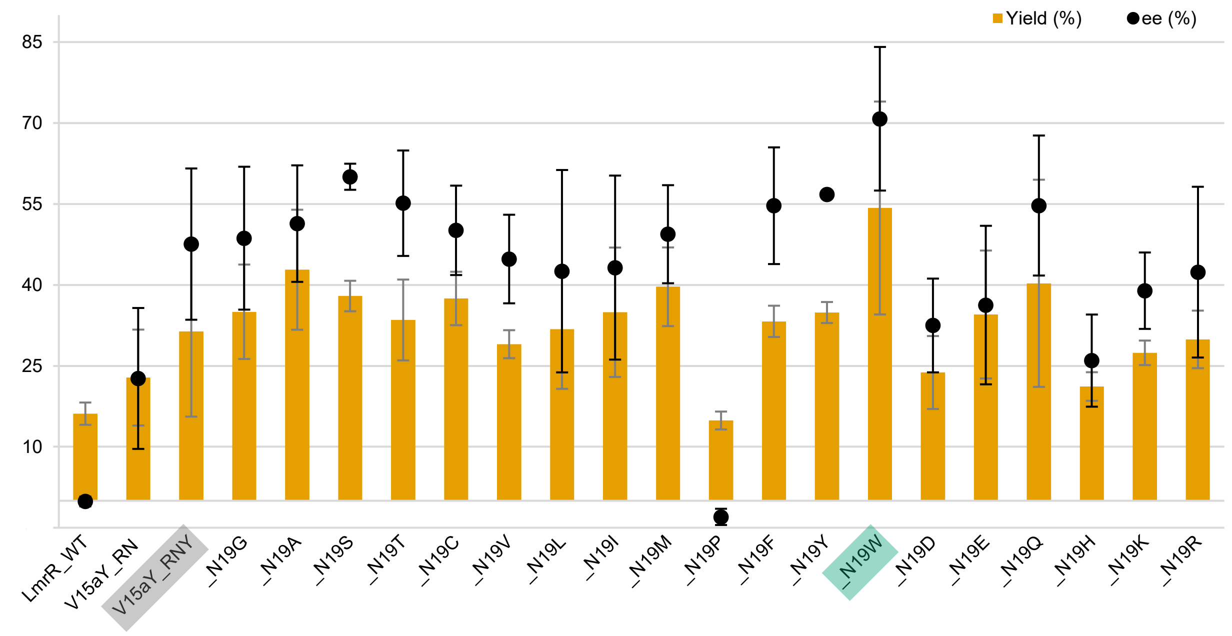

d - RNYW_M8 site saturation and D100 library (150 µL CFE)

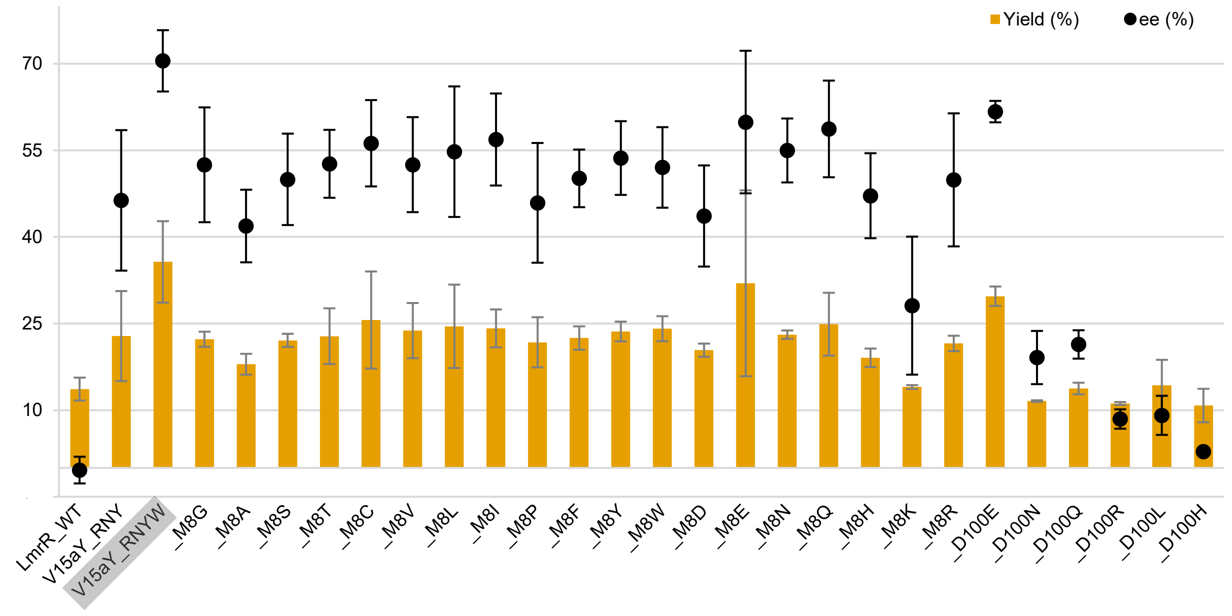

Figure S8. FC-alkylation between **1a** and **2a**, using mutant library cell-lysates obtained from 24-well deep well plate cultures. (a) V15aY_RN_A92 library. (b) V15aY_RNY_M8 library. (c) V15aY_RNY_N19 library. (d) V15aY_RNYW_M8-D100 library. For each screening, the parent is highlighted in grey. Mutants chosen as improved variants are highlighted in green. In every screen, LmrR_WT and a previous V15aY variant were also performed as controls. Reaction conditions: cell lysate (up to 276 µL, amounts used for catalysis are shown for each screening), **1a** (15 mM), **2a** (1 mM) in MES buffer (20 mM, pH 5.5), containing NaCl (150 mM) and DMF (8% v/v) in a total volume of 300 µL, continuously inverted for 16 h at 8 °C. Results are based on three biological replicates. Errors are the standard deviation of the results. For more details on preparation of cell-lysates, see ESI section 6. For FC-alkylation results of purified hits see **Table S1**.

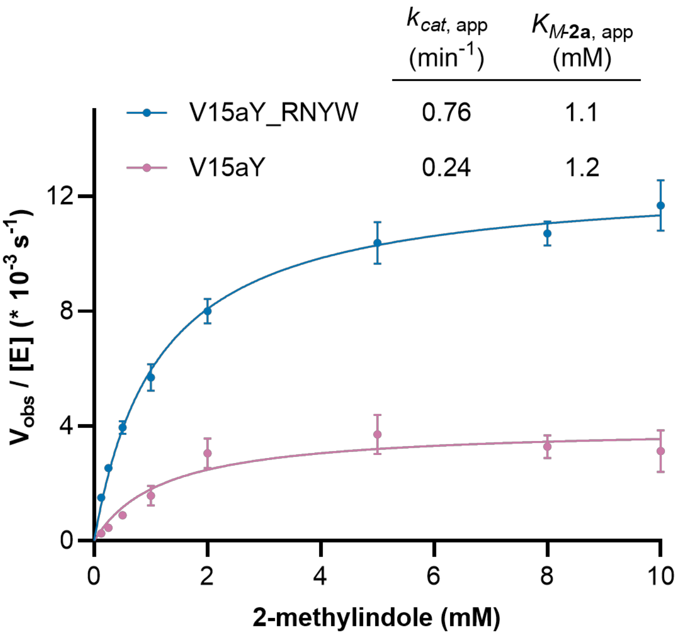

**Catalyst**	**$k_{cat,\,app}$ (95% CI)* (* 10^{-3} s^{-1})**	**$K_{M\text{-}2a,\,app}$ (95% CI)* (mM)**	**$(k_{cat}$ / $K_{M\text{-}2a})_{app}$ (M^{-1} s^{-1})**	**R^2 of fit**
V15aY	3.977 (3.446 - 4.614)	1.213 (0.7445 - 1.988)	3.279	0.8629
V15aY_RNYW	12.61 (12.06 - 13.18)	1.123 (0.9571 - 1.319)	11.23	0.9843

Figure S9. Apparent Michaelis-Menten kinetics for the FC-alkylation reaction between **1a** and **2a**, catalysed by V15aY or V15aY_RNYW. Reaction conditions: V15aY (30 µM) or V15aY_RNYW (10 µM), **1a** (25 mM), **2a** (0.125-10 mM) in MES buffer (20 mM, pH 5.5), containing NaCl (150 mM) and DMF (8% v/v) in a total volume of 300 µL. Reactions were incubated at 25 °C and quenched by addition of $NaBH_4$ (60 µL, 20 mg mL^{-1} in 0.5% w/v NaOH) at three different time points between 5-60 min. Results are based on at least three experiments, using two or more independently produced batches of protein. Errors are the standard deviation of the results. Plotted initial rates are corrected for background activity, determined in the same way, but in the absence of protein. *Apparent kinetic parameters are reported as 95% confidence intervals. For experimental details, see ESI section 9.

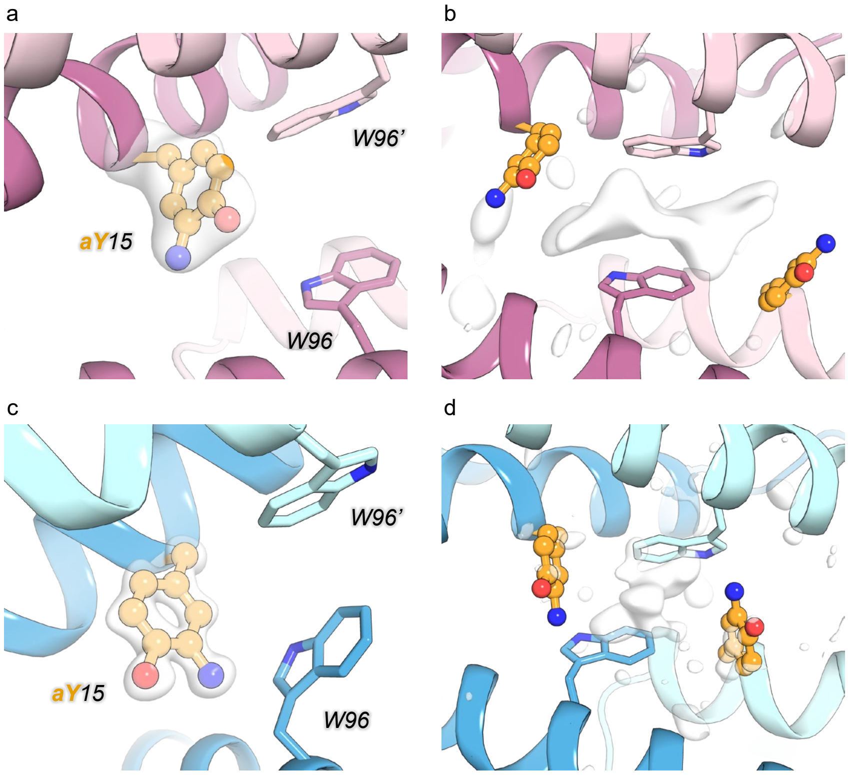

Figure S10. Zoomed-in views of the LmrR_V15aY structures highlighting difference electron density maps. (a) Close-up of 3-aminotyrosine at position 15 in the LmrR_V15aY parent structure, showing its F_o-F_c omit map electron density. (b) View of the dimeric interface in the LmrR_V15aY parent, highlighting residual F_o-F_c electron density in between the W96/W96' residues. This density suggests the presence of a bound ligand, although its identity could not be definitively determined. (c) Close-up of 3-aminotyrosine at position 15 in LmrR_V15aY_RNYW_KK, showing its F_o-F_c omit map electron density. (d) The F_o-F_c electron density map shown at the dimeric interface of LmrR_V15aY_RNYW_KK also reveals residual density between the W96/W96' residues, indicating the presence of a bound ligand. However, also in this case, its identity could not be definitively determined. The F_o-F_c electron density maps are contoured at 2.5σ.

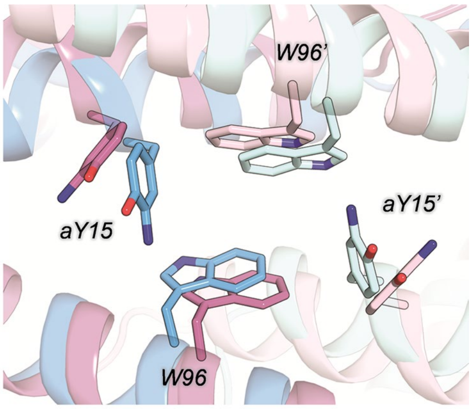

Figure S11. Comparison of LmrR_V15aY (pink) and LmrR_V15aY_RNYW_KK (blue), showing the aY15, aY15' and the central W96, W96' residues.

2. Supporting Tables

Table S1. FC-alkylation of **2a** with **1a**, using purified proteins of focused Q12 or D100 libraries, and hits from cell-lysate screenings of A92, M8 or N19 SSM libraries.

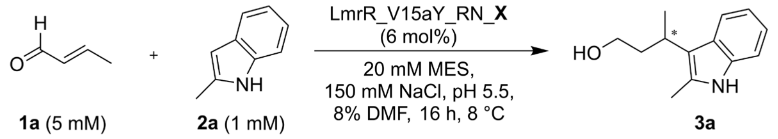

Entry	Catalyst	Yield (%)	ee (%)
1*	LmrR_V15aY_RN (RN)	47 ± 2	71 ± 0
2	RN_Q12N	54 ± 0	– 2 ± 0
3	RN_Q12E	48 ± 1	30 ± 0
4	RN_Q12K	32 ± 0	17 ± 0
5	RN_Q12H	8 ± 0	8 ± 0
6	RN_Q12L	30 ± 2	12 ± 0
7	RN_Q12S	39 ± 0	– 2 ± 0
8	RN_D100E	31 ± 0	46 ± 0
9	RN_D100N	12 ± 0	10 ± 0
10	RN_D100Q	13 ± 0	18 ± 0
11	RN_D100R	7 ± 0	6 ± 0
12	RN_D100L	11 ± 0	6 ± 0
13	RN_D100H	5 ± 0	2 ± 0
14	RN_A92F	79 ± 5	62 ± 0
15*	RN_A92Y (RNY)	76 ± 3	90 ± 0
16	RN_A92W	85 ± 2	63 ± 0
17	RNY_M8I	70 ± 1	90 ± 0
18	RNY_M8W	68 ± 1	89 ± 0
19	RNY_M8E	81 ± 0	90 ± 0
20	RNY_M8R	78 ± 1	92 ± 0
21	RNY_N19A	81 ± 1	83 ± 0
22	RNY_N19S	97 ± 2	87 ± 0
23	RNY_N19Y	93 ± 1	91 ± 0
24*	RNY_N19W (RNYW)	98 ± 6	93 ± 0
25	RNY_N19Q	91 ± 1	88 ± 0
26	RNYW_M8E	82 ± 1	91 ± 0
27	RNYW_D100E	91 ± 0	88 ± 0

Reaction Conditions: V15aY_RN variant (60 µM), **1a** (5 mM), **2a** (1 mM) in MES buffer (20 mM, pH 5.5), containing NaCl (150 mM) and DMF (8% v/v) in a total volume of 300 µL, continuously inverted for 16 h at 8 °C. Unless otherwise noted, results are the average of technical duplicates. *Entries are based on at least three experiments, using two or more independently produced batches of protein. Errors are the standard deviation of the results. ee is assigned relative to the enantiomer obtained with V15aY, in which the "–" symbol represents the opposite enantiomer.

Table S2. FC-alkylation of **2a** with **1a**, using purified LmrR_V15aY_RNYW with varying reaction conditions.

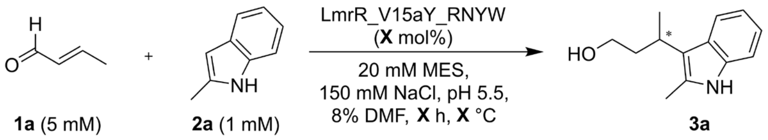

Entry	Catalyst loading	Reaction time	Reaction temperature	Yield (%)	ee (%)
1*	6 mol%	16 h	8 °C	98 ± 6	93 ± 0
2	6 mol%	6 h	8 °C	67	92
3*	3 mol%	16 h	8 °C	80 ± 2	92 ± 0
4	3 mol%	6 h	8 °C	39	91
5	6 mol%	6 h	25 °C	86	92
6	6 mol%	3 h	25 °C	64	93
7	3 mol%	6 h	25 °C	65	91
8	3 mol%	3 h	25 °C	39	90

Reaction conditions: V15aY_RNYW: (30 or 60 µM), **1a** (5 mM), **2a** (1 mM) in MES buffer (20 mM, pH 5.5), containing NaCl (150 mM) and DMF (8% v/v) in a total volume of 300 µL, continuously inverted for the specified amount of time at the specified temperature. Unless otherwise noted, results are based on one experiment. *Entries are based on at least three experiments, using two or more independently produced batches of protein. Errors are the standard deviation of the results.

Table S3. FC-alkylation controls with purified proteins, including LmrR_V15Y/V15pAF_RNYW.

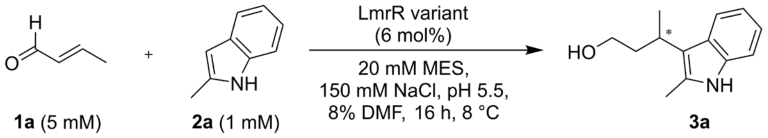

Entry	Catalyst	Yield (%)	ee (%)
1*	LmrR_WT	5 ± 0	6 ± 1
2	LmrR_V15Y_RNYW	4 ± 0	– 2 ± 0
3[†]	V15aY_RNYW (denatured)	17 ± 0	< 1
4[#]	V15aY_RNYW_D55K_Q59K	88	92
5	LmrR_V15pAF	59 ± 0	– 34 ± 0
6	LmrR_V15pAF_RNYW	67 ± 0	– 36 ± 0

Reaction conditions: LmrR variant (60 µM), **1a** (5 mM), **2a** (1 mM) in MES buffer (20 mM, pH 5.5), containing NaCl (150 mM) and DMF (8% v/v) in a total volume of 300 µL, continuously inverted for 16 h at 8 °C. Unless otherwise noted, results are the average of technical duplicates. Errors are the standard deviation of the results. *Based on at least three experiments, using two or more independently produced batches of protein. [†]Experiment was performed with V15aY_RNYW that was heat incubated for 10 min at 99 °C prior to setting up catalysis. [#]Results based on one experiment. ee is assigned relative to the enantiomer obtained with V15aY, in which the "–" symbol represents the opposite enantiomer.

Table S4. Summary of the crystallographic statistics.

	LmrR_V15aY	LmrR_V15aY_RNYW_KK
<u>**Data collection**</u>		
Wavelength (Å)	0.96546	0.96546
Resolution range (Å)	61.26 – 2.15 (2.21 – 2.15)	74.00 – 1.20 (1.22 – 1.20)
Space group	$P\ 3_2\ 1\ 2$	$P2_12_12_1$
Unit cell, a, b, c (Å)	70.7, 70.7, 59.8	45.7, 58.5, 74.0
R_{merge}	0.050 (1.760)	0.062 (2.060)
R_{meas}	0.053 (1.951)	0.065 (2.360)
R_{pim}	0.016 (0.603)	0.018 (0.803)
Number of observations	96934 (7881)	770092 (23880)
Number unique	17381 (813)	62694 (2937)
Mean(I/σI)	17.4 (1.0)	17.4 (1.0)
$CC_{1/2}$	1.000 (0.705)	1.000 (0.409)
Completeness (%)	100.0 (100.0)	99.8 (96.0)
Multiplicity	10.2 (10.3)	12.3 (8.1)
<u>**Refinement**</u>		
Resolution (Å)	42.84 – 2.15	45.95 – 1.20
R_{work} / R_{free}	0.221 / 0.268	0.174 / 0.213
Content AU,	113 protein residues (1 chain), 5 waters	219 protein residues (2 chains), 3 nitrate, 208 waters
nr of non-H atoms, protein, waters, other	915, 5, 13	1865, 208, 12
Average B-factors (Å^2) protein, water, other	77.1, 66.3, 63.1	20.2, 31.0, 21.7
RMSD bond lengths (Å), angles (°)	0.010, 0.8	0.010, 1.7
Ramachandran favored, outliers (%)	100.0, 0.0	100.0, 0.0
Rotamers, outliers (%)	2.0	0.0
Molprobity-Clashscore	3.26	2.64
PDB entry	9H87	9H88

Values in parentheses refer to the highest resolution shell. AU, asymmetric unit

Table S5. Selected scope of FC-alkylation reaction performed at 25 °C.

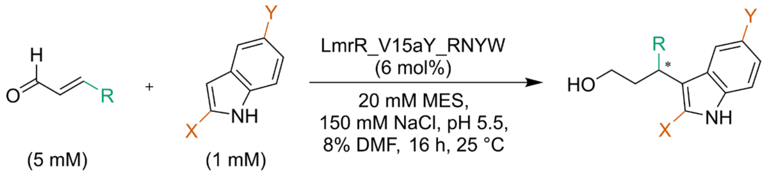

Entry	Product	Yield (%)	ee (%)
1	**3c**	17 ± 0	75 ± 1
2	**3e**	31 ± 2	94 ± 0
3	**3g**	17 ± 1	– 84 ± 1
4	**3h**	5 ± 1	88 ± 1

Reaction conditions: V15aY_RNYW (60 µM), β-substituted-α,β-unsaturated aldehyde (5 mM), indole substrate (1 mM) in MES buffer (20 mM, pH 5.5), containing NaCl (150 mM) and DMF (8% v/v) in a total volume of 300 µL, continuously inverted for 16 h at 25 °C. Entries are based on at least three experiments, using two or more independently produced batches of protein. Errors are the standard deviation of the results. ee is assigned relative to the enantiomer obtained with V15aY, in which the "–" symbol represents the opposite enantiomer.

Table S6. FC-alkylation of **2a** with **1a** using whole-cells expressing an LmrR variant, conditions screening and controls.

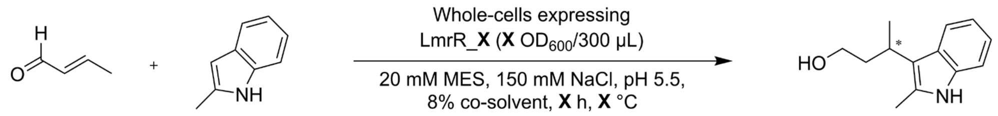

1a (**X** mM) **2a** (1 mM) **3a**

Entry	Expressed protein	Whole-cell loading (OD_{600} units /300 µL reaction)	[1a]	Co-solvent (8%)	Reaction temperature	Reaction time	Yield (%)	ee (%)
1	V15aY	2	15 mM	DMF	8 °C	24 h	19	15
2	V15aY_RNYW	2	15 mM	DMF	8 °C	24 h	53	66
3	V15aY	8	15 mM	DMF	8 °C	24 h	22	24
4	V15aY_RNYW	8	15 mM	DMF	8 °C	24 h	84	79
5	V15aY	16	15 mM	DMF	8 °C	24 h	20	31
6	V15aY_RNYW	16	15 mM	DMF	8 °C	24 h	84	83
7	LmrR_WT	6	15 mM	DMF	8 °C	21 h	25	<1
8	V15aY_RNYW	6	15 mM	DMF	8 °C	21 h	75	76
9	LmrR_WT	6	15 mM	DMSO	8 °C	21 h	27	– 1
10*	V15aY_RNYW	6	15 mM	DMSO	8 °C	21 h	73 ± 2	75 ± 1
11#	V15aY_RNYW (Supernatant)	6	15 mM	DMSO	8 °C	21 h	69	76
12#	V15aY_RNYW (Pellet)	6	15 mM	DMSO	8 °C	21 h	2	77
13	LmrR_WT	6	5 mM	DMSO	8 °C	21 h	11	– 1
14	V15aY_RNYW	6	5 mM	DMSO	8 °C	21 h	53	81
15	LmrR_WT	6	15 mM	DMF	25 °C	21 h	31	<1
16	V15aY_RNYW	6	15 mM	DMF	25 °C	21 h	64	71
17	LmrR_WT	6	15 mM	DMSO	25 °C	21 h	31	<1
18	V15aY_RNYW	6	15 mM	DMSO	25 °C	21 h	60	70
19	LmrR_WT	6	5 mM	DMSO	25 °C	21 h	16	– 2
20	V15aY_RNYW	6	5 mM	DMSO	25 °C	21 h	52	76
21	LmrR_WT	6	15 mM	DMF	25 °C	4 h	14	<1
22	V15aY_RNYW	6	15 mM	DMF	25 °C	4 h	58	73
23	LmrR_WT	6	15 mM	DMSO	25 °C	4 h	17	<1
24	V15aY_RNYW	6	15 mM	DMSO	25 °C	4 h	57	71
25	LmrR_WT	6	5 mM	DMSO	25 °C	4 h	6	– 2
26	V15aY_RNYW	6	5 mM	DMSO	25 °C	4 h	34	78

Reaction conditions: washed whole-cells (BL21(DE3) C41) expressing LmrR variant (specified OD_{600} units), **1a** (specified mM), **2a** (1 mM) in MES buffer (20 mM, pH 5.5), containing NaCl (150 mM) and co-solvent (8% v/v) in a total volume of 300 µL, continuously inverted for the specified amount of time and temperature. Unless otherwise noted, results are based on one experiment. *Based on at least three experiments, using two or more independently produced batches of protein. Errors are the standard deviation of the results. #Results from separate extraction of supernatant or of the pellet of spun down reaction mixture.

3. General Considerations

All commercial reagents were used as received from vendors without further purification, unless otherwise specified. aY was purchased from Merck (3-amino-*L*-tyrosine dihydrochloride monohydrate, 98%) and pAzF was purchased from Iris-Biotech (4-Azido-*L*-phenylalanine hydrochloride, 99%). 2-methylindole (>99%) was purchased from TCI and crotonaldehyde was purchased from Merck (crotonaldehyde, mixture of *cis* and *trans* ~1:20, >99.5%). Plasmid pEvol_*Mj*aYRS was obtained from Addgene (plasmid #153557), and was a gift from Huiwang Ai.[2] Plasmid pEvol_pAzFRS.2.t1 was obtained from Addgene (plasmid #73546), and was a gift from Farren Isaacs.[3] An Eppendorf Mastercycle Nexus 2 was used to perform PCRs and primers were synthesized by Eurofins Genomics GmbH and/or Merck. Plasmid purification (QIAprep Spin Miniprep) and PCR purification (QIAquick) kits were purchased from QIAGEN. DNA sequencing was carried out by Eurofins Genomics GmbH. A standard recipe for Luria-Bertani (LB) medium was used (10 g/L bacto tryptone, 5 g/L yeast extract, 10 g/L NaCl and 15 g/L agar for LB-Agar plates). 1000X Antibiotic and inducer stock solutions were used and prepared as follows: ampicillin (100 mg/mL in MilliQ); chloramphenicol (34 mg/mL in 96% EtOH); *L*-arabinose (0.2 g/mL in MilliQ); isopropyl *β*-D-1-thiogalactopyranoside (IPTG) (1 M in MilliQ), and were sterilized through filtration using a 0.2 µm syringe filter (Whatman). Cultures were cultivated in a New Brunswick Innova 42 orbital shaker at a speed of 180 rpm (1 inch stroke). Deep well plates were cultivated in a Heidolph Titramax 1000. Eppendorf 5810R / 5920R benchtop centrifuges were used for spinning down cultures, tubes and plates. Sonication was performed with a Qsonica Q125 sonicator equipped with a 1/4" (6.4mm) probe. SDS-PAGE gels were stained using InstantBlue (abcam). Strep-Tactin resin (Strep-Tactin Superflow high capacity) and Desthiobiotin were purchased from IBA-Lifesciences. Unless otherwise specified, phosphate-buffered saline (PBS) was prepared with 50 mM Na_2HPO_4 and 150 mM NaCl. DNA and protein concentrations were determined based on A_{260} and A_{280} values measured using a Thermo Scientific Nanodrop 2000 UV-Vis spectrophotometer. Concentrations of LmrR throughout the manuscript are reported as dimeric concentrations. Vivaspin 20 10 kDa MWCO (Sartorius) and Amicon Ultra 0.5 mL 10 kDa MWCO (Millipore) centrifugal units were used for concentrating proteins. Buffer exchange was performed with PD-10 or PD-Minitrap Sephadex G-25 M desalting columns (Cytiva). Analytical thin-layer chromatography (TLC) was carried out using Supelco silica gel 60 F_{254} plates. Visualization was accomplished with UV light followed by dipping in potassium permanganate or vanillin stain solution and then heating. Purification of reactions was carried out by flash chromatography under positive pressure using Supelco silica gel (60 Å, 230-400 mesh, 40-63 µm). Nuclear magnetic resonance spectra (^1H and ^{13}C NMR experiments) were recorded on a Varian 400 MHz or a Bruker 600 MHz. Chemical shifts (δ) for proton and carbon are reported in parts per million (ppm) downfield from tetramethylsilane and are referenced to the proton or carbon resonance of residual $CHCl_3$ (δ_H = 7.26 ppm, δ_C = 77.0 ppm). NMR data are represented as follows: chemical shift (ppm), multiplicity (s = singlet, d = doublet, t = triplet, q = quartet, hept = heptet, m = multiplet), coupling constant in Hertz (Hz), and integration. HPLC analysis was performed using a Shimadzu Prominence LC-20AD equipped with a Shimadzu SPD-M20A diode array detector. SFC analysis was performed using a Waters Acquity UPC2 system. Routine protein mass spectrometry measurements were performed using a Waters Acquity H-class UPLC coupled to a Waters Xevo G2 QTOF. High-Resolution Mass Spectrometry (HRMS) measurements for small molecules and proteins were performed using a Thermo Scientific Vanquish LC connected to an Orbitrap Exploris 480. FPLC was performed using an Äkta Purifier system equipped with a UV-900 Detector. UV-Vis absorption spectra were recorded at 25 °C on a Jasco V-660 spectrophotometer.

4. Molecular Biology

Site-directed mutagenesis

pET17b_LmrR_WT, pET17b_LmrR_V15X ("X" represents the amber stop codon, TAG), pET17b_LmrR_V15X_R / G / RG / RGN, pET17b_LmrR_V15X_RMH and pET17b_LmrR_V15Y_ RGN / RMH, all including mutations for decreased DNA-binding (K55D_K59Q), were available from previous works.[1,4,5] Plasmids pET17b_LmrR_V15X and pET17b_LmrR_V15X_R / G were used as initial templates for site-directed mutagenesis. PCRs were performed with Phusion HF polymerase (1U/50 µL) (New England Biolabs) using Phusion HF buffer (1X), dNTPs (200 µM), forward primer (0.5 µM), reverse primer (0.5 µM), template plasmid DNA (1-10 ng), DMSO (3% v/v), and sterilized MilliQ water in a total reaction volume of 20-50 µL, or with Phusion Flash HF Master mix (1X) (Thermo Scientific) using forward primer (0.5 µM), reverse primer (0.5 µM), template plasmid DNA (1-10 ng) and sterilized MilliQ water in a total reaction volume of 20-50 µL. In general, the following PCR-protocol was used: (1) initial denaturation at 98 °C for 2 min, (2) 16 cycles of denaturation at 98 °C for 10 s, annealing at T_m - 5 °C for 30 s (T_m of used primers calculated using the oligo analysis tool from Eurofins Genomics), and extension at 72 °C for 2.5 min, (3) final extension at 72 °C for 10 min. Annealing temperatures used for successful PCRs ranged from 55 to 67 °C. For some mutants, a touchdown PCR protocol was followed: 1) initial denaturation at 98 °C for 5 min, (2) 20 cycles of denaturation at 98 °C for 30 s, annealing for 30 s from 65 °C to 45 °C with steps of -1 °C/cycle, and extension at 72 °C for 2 min and 10 s, (3) 10 cycles of denaturation at 98 °C for 30 s, annealing at 45 °C for 30 s, and extension at 72 °C for 2 min and 10 s, (4) final extension at 72 °C for 5 min. PCR products were treated with 0.5-1 µL restriction enzyme *DpnI* (New England Biolabs) for 1-2 h at 37 °C and subsequently purified using a QIAquick PCR purification kit. Transformation was performed by addition of 1-5 µL of PCR product to 50 µL chemically competent *E.coli* NEB10β (prepared according to the Inoue method)[6], followed by 30 min incubation on ice, a 45 s heat shock at 42 °C and another 2 min incubation on ice directly after. Cells were then recovered in 0.7 mL SOC medium at 37 °C for ≈ 1 h in a thermomixer (750 rpm). The transformation mixture was subsequently spun down at 3000 xg and cells resuspended in 100-200 µL of residual volume. 20-100% of the cells was then plated on LB-agar plates containing ampicillin (100 µg/mL) and incubated at 37 °C overnight. Single colonies were inoculated into 5 mL LB medium containing ampicillin (100 µg/mL) and incubated at 37 °C in an orbital shaker overnight. Glycerol stocks were prepared by mixing 650 µL of cell culture with 350 µL of sterile 50% (v/v) glycerol and stored at -70 °C. Plasmid DNA was isolated using a QIAprep Spin Miniprep kit and sent for sanger sequencing using T7 or T7-term primer to confirm correct mutation of the target gene. Upon confirmation of successful mutation, 1-5 µL of isolated plasmid was transformed as described above into chemically competent *E. coli* BL21(DE3) C41 harbouring pEvol_*Mj*aYRS (prepared according to the Inoue method)[6], plated 50-100% on LB-agar plates containing ampicillin (100 µg/mL) and chloramphenicol (34 µg/mL), and incubated at 37 °C overnight. Single colonies were inoculated into 5 mL LB medium containing ampicillin (100 µg/mL) and chloramphenicol (34 µg/mL) and incubated at 37 °C in an orbital shaker overnight. Glycerol stocks were prepared by mixing 650 µL of cell culture with 350 µL of sterile 50% (v/v) glycerol and stored at -70 °C.

Gene sequences (DNA / protein) LmrR_V15aY and LmrR_V15aY_RNYW

The amber stop codon and the ncAA are depicted in pink, the RNYW mutations in blue and the C-terminal StrepTag for affinity purification in green. The N-terminal methionine of LmrR is generally cleaved off during protein production in *E. coli*. Note that, compared to the sequence of native LmrR (Uniprot A2RI36), the LmrR gene used in this work contains an additional glycine directly after methionine 1. To keep amino acid numbering consistent with native LmrR, the N-terminal methionine that is cleaved off is not considered in amino acid numbering.

LmrR_V15aY:

ATGGGTGCCGAAATCCCGAAAGAAATGCTGCGTGCTCAAACCAATTAGATCCTGCTGAATGTCCTGAAACAAGGCGATAACTATGTGTATGGCATTATCAAACAG
GTGAAAGAAGCGAGCAACGGTGAAATGGAACTGAATGAAGCCACCCTGTATACGATTTTTGATCGTCTGGAACAGGACGGCATTATCAGCTCTTACTGGGGTGA
TGAAAGTCAAGGCGGTCGTCGCAAATATTACCGTCTGACCGAAATCGGCCATGAAAACATGCGCCTGGCGTTCGAATCCTGGAGTCGTGTGGACAAAATCATTG
AAAATCTGGAAGCAAACAAAAAATCTGAAGCGATCAAATCTAGAGGTGGCAGCGGTGGCTGGAGCCACCCGCAGTTCGAAAAATAA

~~M~~GAEIPKEMLRAQTNaYILLNVLKQGDNYVYGIIKQVKEASNGEMELNEATLYTIFDRLEQDGIISSYWGDESQGGRRKYYRLTEIGHENMRLAFESWSRVDKIIENLEAN
KKSEAIKSRGGSGGWSHPQFEK*

LmrR_V15aY_RNYW:

ATGGGTGCCGAAATCCCGAAAGAAATGCTGCGTGCTCAAACCAATTAGATCCTGCGTTGGGTCCTGAAACAAGGCGATAACTATGTGTATGGCATTATCAAACAG
GTGAAAGAAGCGAGCAACGGTGAAATGGAACTGAATGAAGCCACCCTGTATACGATTTTTGATCGTCTGGAACAGGACGGCATTATCAGCTCTTACTGGGGTGA
TGAAAGTCAAGGCGGTCGTCGCAAATATTACCGTCTGACCGAAATCGGCCATGAAAACAACCGCCTGTATTTCGAATCCTGGAGTCGTGTGGACAAAATCATTGA
AAATCTGGAAGCAAACAAAAAATCTGAAGCGATCAAATCTAGAGGTGGCAGCGGTGGCTGGAGCCACCCGCAGTTCGAAAAATAA

~~M~~GAEIPKEMLRAQTNaYILRWVLKQGDNYVYGIIKQVKEASNGEMELNEATLYTIFDRLEQDGIISSYWGDESQGGRRKYYRLTEIGHENNRLYFESWSRVDKIIENLEAN
KKSEAIKSRGGSGGWSHPQFEK*

Primer list (5' to 3')

Primer name	Forward primer sequence	Primer name	Reverse primer sequence	Template Gene(s) (LmrR_)
M89N_fw	GGCCATGAAAACAACCGCCTGGCGTTCGAATC	M89N_rev	GATTCGAACGCCAGGCGGTTGTTTTCATGGCC	V15X, V15X_R
M89N_fw_2	GGCCATGAAAACAACCGCCTGGCGTTCG	M89N_rev_2	CGAACGCCAGGCGGTTGTTTTCATGGCC	V15X_G
M8A_fw	GAAATCCCGAAAGAAGCGCTGCGTGCTC	M8A_rev	GAGCACGCAGCGCTTCTTTCGGGATTTC	V15X_RN, V15X_RNY, V15X_RNYW
A11L_fw	CGAAAGAAATGCTGCGTCTGCAAACCAATTAGATC	A11_rev	GATCTAATTGGTTTGCAGACGCAGCATTTCTTTCG	V15X_RN
Q12A_fw	GAAATGCTGCGTGCTGCGACCAATTAGATCC	Q12_rev	GGATCTAATTGGTCGCAGCACGCAGCATTTC	V15X_RN
N19A_fw	GATCCTGCGTGCGGTCCTGAAACAAGG	N19_rev	CCTTGTTTCAGGACCGCACGCAGGATC	V15X_RN, V15X_RNY
K22A_fw	GCGTAATGTCCTGGCGCAAGGCGATAAC	K22_rev	GTTATCGCCTTGCGCCAGGACATTACGC	V15X_RN
N88A_fw	GAAATCGGCCATGAAGCGAACCGCCTG	N88_rev	CAGGCGGTTCGCTTCATGGCCGATTTC	V15X_RN
A92L_fw	CAACCGCCTGCTGTTCGAATCCTGGAG	A92_rev	CTCCAGGATTCGAACAGCAGGCGGTTG	V15X_RN
F93A_fw	CCTGGCGGCGGAATCCTGGAGTC	F93_rev	GACTCCAGGATTCCGCCGCCAGG	V15X_RN
W96A_fw	CGTTCGAATCCGCGAGTCGTGTGGAC	W96_rev	GTCCACACGACTCGCGGATTCGAACG	V15X_RN
S97A_fw	GTTCGAATCCTGGGCGCGTGTGGAC	S97_rev	GTCCACACGCGCCCAGGATTCGAAC	V15X_RN
D100A_fw	CCTGGAGTCGTGTGGCGAAAATCATTGAAAATCTG	D100_rev	CAGATTTTCAATGATTTTCGCCACACGACTCCAGG	V15X_RN
I103A_fw	CGTGTGGACAAAATCGCGGAAAATCTGGAAGC	I103_rev	GCTTCCAGATTTTCCGCGATTTTGTCCACACG	V15X_RN
Q12N_fw	GCGTGCTAACACCAATTAGATCCTGCG	Q12N_rev	CTAATTGGTGTTAGCACGCAGCATTTCTTTCG	V15X_RN
Q12E_fw	GCGTGCTGAAACCAATTAGATCCTGCG	Q12E_rev	CTAATTGGTTTCAGCACGCAGCATTTCTTTCG	V15X_RN
Q12K_fw	GCGTGCTAAAACCAATTAGATCCTGCG	Q12K_rev	CTAATTGGTTTTAGCACGCAGCATTTCTTTCG	V15X_RN
Q12H_fw	GCGTGCTCATACCAATTAGATCCTGCG	Q12H_rev	CTAATTGGTATGAGCACGCAGCATTTCTTTCG	V15X_RN
Q12L_fw	GCGTGCTCTGACCAATTAGATCCTGCG	Q12L_rev	CTAATTGGTCAGAGCACGCAGCATTTCTTTCG	V15X_RN
Q12S_fw	GAAATGCTGCGTGCTAGCACCAATTAGATCC	Q12S_rev	GGATCTAATTGGTGCTAGCACGCAGCATTTC	V15X_RN
D100E_fw	CGTGTGGAAAAAATCATTGAAAATCTGGAAGC	D100E_rev	CAATGATTTTTTCCACACGACTCCAGG	V15X_RN, V15X_RNYW
D100N_fw	CGTGTGAACAAAATCATTGAAAATCTGGAAGC	D100N_rev	CAATGATTTTGTTCACACGACTCCAGG	V15X_RN, V15X_RNYW
D100Q_fw	CGTGTGCAGAAAATCATTGAAAATCTGGAAGC	D100Q_rev	CAATGATTTTCTGCACACGACTCCAGG	V15X_RN, V15X_RNYW

D100R_fw	CCTGGAGTCGTGTGCGTAAAATCATTGAAAATCTG	D100R_rev	CAGATTTTCAATGATTTTACGCACACGACTCCAGG	V15X_RN
D100L_fw	CGTGTGCTGAAAATCATTGAAAATCTGGAAGC	D100L_rev	CAATGATTTTCAGCACACGACTCCAGG	V15X_RN, V15X_RNYW
D100H_fw	CGTGTGCATAAAATCATTGAAAATCTGGAAGC	D100H_rev	CAATGATTTTATGCACACGACTCCAGG	V15X_RN, V15X_RNYW
A92G_fw	CAACCGCCTGGGCTTCGAATCCTGG	A92G_rev	CCAGGATTCGAAGCCCAGGCGGTTG	V15X_RN
A92S_fw	CAACCGCCTGAGCTTCGAATCCTGG	A92S_rev	CCAGGATTCGAAGCTCAGGCGGTTG	V15X_RN
A92T_fw	CAACCGCCTGACCTTCGAATCCTGG	A92T_rev	CCAGGATTCGAAGGTCAGGCGGTTG	V15X_RN
A92C_fw	CAACCGCCTGTGCTTCGAATCCTGG	A92C_rev	CCAGGATTCGAAGCACAGGCGGTTG	V15X_RN
A92V_fw	CAACCGCCTGGTGTTCGAATCCTGG	A92V_rev	CCAGGATTCGAACACCAGGCGGTTG	V15X_RN
A92I_fw	CAACCGCCTGATTTTCGAATCCTGG	A92I_rev	CCAGGATTCGAAAATCAGGCGGTTG	V15X_RN
A92M_fw	CAACCGCCTGATGTTCGAATCCTGG	A92M_rev	CCAGGATTCGAACATCAGGCGGTTG	V15X_RN
A92P_fw	CAACCGCCTGCCGTTCGAATCCTGG	A92P_rev	CCAGGATTCGAACGGCAGGCGGTTG	V15X_RN
A92F_fw	CGCCTGTTTTTCGAATCCTGGAGTC	A92F_rev	GATTCGAAAAACAGGCGGTTGTTTTCATG	V15X_RN
A92Y_fw	CGCCTGTATTTCGAATCCTGGAGTC	A92Y_rev	GATTCGAAATACAGGCGGTTGTTTTCATG	V15X_RN
A92W_fw	CAACCGCCTGTGGTTCGAATCCTGG	A92W_rev	CCAGGATTCGAACCACAGGCGGTTG	V15X_RN
A92D_fw	CAACCGCCTGGATTTCGAATCCTGG	A92D_rev	CCAGGATTCGAAATCCAGGCGGTTG	V15X_RN
A92E_fw	CAACCGCCTGGAATTCGAATCCTGG	A92E_rev	CCAGGATTCGAATTCCAGGCGGTTG	V15X_RN
A92N_fw	CAACCGCCTGAACTTCGAATCCTGG	A92N_rev	CCAGGATTCGAAGTTCAGGCGGTTG	V15X_RN
A92Q_fw	CAACCGCCTGCAGTTCGAATCCTGG	A92Q_rev	CCAGGATTCGAACTGCAGGCGGTTG	V15X_RN
A92H_fw	CGCCTGCATTTCGAATCCTGGAGTC	A92H_rev	GATTCGAAATGCAGGCGGTTGTTTTCATG	V15X_RN
A92K_fw	CGCCTGAAATTCGAATCCTGGAGTC	A92K_rev	GATTCGAATTTCAGGCGGTTGTTTTCATG	V15X_RN
A92R_fw	CAACCGCCTGCGTTTCGAATCCTGG	A92R_rev	CCAGGATTCGAAACGCAGGCGGTTG	V15X_RN
M8G_fw	GAAATCCCGAAAGAAGGCCTGCGTGCTC	M8G_rev	GAGCACGCAGGCCTTCTTTCGGGATTTC	V15X_RNY, V15X_RNYW
M8S_fw	GAAATCCCGAAAGAAAGCCTGCGTGCTC	M8S_rev	GAGCACGCAGGCTTTCTTTCGGGATTTC	V15X_RNY, V15X_RNYW
M8T_fw	GAAATCCCGAAAGAAACCCTGCGTGCTC	M8T_rev	GAGCACGCAGGGTTTCTTTCGGGATTTC	V15X_RNY, V15X_RNYW
M8C_fw	GAAATCCCGAAAGAATGCCTGCGTGCTC	M8C_rev	GAGCACGCAGGCATTCTTTCGGGATTTC	V15X_RNY, V15X_RNYW
M8V_fw	GAAATCCCGAAAGAAGTGCTGCGTGCTC	M8V_rev	GAGCACGCAGCACTTCTTTCGGGATTTC	V15X_RNY, V15X_RNYW
M8L_fw	GAAATCCCGAAAGAACTGCTGCGTGCTC	M8L_rev	GAGCACGCAGCAGTTCTTTCGGGATTTC	V15X_RNY, V15X_RNYW
M8I_fw	GAAATCCCGAAAGAAATTCTGCGTGCTC	M8I_rev	GAGCACGCAGAATTTCTTTCGGGATTTC	V15X_RNY, V15X_RNYW
M8P_fw	GAAATCCCGAAAGAACCGCTGCGTGCTC	M8P_rev	GAGCACGCAGCGGTTCTTTCGGGATTTC	V15X_RNY, V15X_RNYW
M8F_fw	GAAATCCCGAAAGAATTTCTGCGTGCTC	M8F_rev	GAGCACGCAGAAATTCTTTCGGGATTTC	V15X_RNY, V15X_RNYW
M8Y_fw	GAAATCCCGAAAGAATATCTGCGTGCTC	M8Y_rev	GAGCACGCAGATATTCTTTCGGGATTTC	V15X_RNY, V15X_RNYW
M8W_fw	GAAATCCCGAAAGAATGGCTGCGTGCTC	M8W_rev	GAGCACGCAGCCATTCTTTCGGGATTTC	V15X_RNY, V15X_RNYW
M8D_fw	GAAATCCCGAAAGAAGATCTGCGTGCTC	M8D_rev	GAGCACGCAGATCTTCTTTCGGGATTTC	V15X_RNY, V15X_RNYW

M8E_fw	GAAATCCCGAAAGAAGAACTGCGTGCTC	M8E_rev	GAGCACGCAGTTCTTCTTTCGGGATTTC	V15X_RNY, V15X_RNYW
M8N_fw	GAAATCCCGAAAGAAAACCTGCGTGCTC	M8N_rev	GAGCACGCAGGTTTTCTTTCGGGATTTC	V15X_RNY, V15X_RNYW
M8Q_fw	GAAATCCCGAAAGAACAGCTGCGTGCTC	M8Q_rev	GAGCACGCAGCTGTTCTTTCGGGATTTC	V15X_RNY, V15X_RNYW
M8H_fw	GAAATCCCGAAAGAACATCTGCGTGCTC	M8H_rev	GAGCACGCAGATGTTCTTTCGGGATTTC	V15X_RNY, V15X_RNYW
M8K_fw	GAAATCCCGAAAGAAAAACTGCGTGCTC	M8K_rev	GAGCACGCAGTTTTTCTTTCGGGATTTC	V15X_RNY, V15X_RNYW
M8R_fw	GAAATCCCGAAAGAACGTCTGCGTGCTC	M8R_rev	GAGCACGCAGACGTTCTTTCGGGATTTC	V15X_RNY, V15X_RNYW
N19G_fw	GATCCTGCGTGGCGTCCTGAAACAAGG	N19G_rev	CCTTGTTTCAGGACGCCACGCAGGATC	V15X_RNY
N19S_fw	GATCCTGCGTAGCGTCCTGAAACAAGG	N19S_rev	CCTTGTTTCAGGACGCTACGCAGGATC	V15X_RNY
N19T_fw	GATCCTGCGTACCGTCCTGAAACAAGG	N19T_rev	CCTTGTTTCAGGACGGTACGCAGGATC	V15X_RNY
N19C_fw	GATCCTGCGTTGCGTCCTGAAACAAGG	N19C_rev	CCTTGTTTCAGGACGCAACGCAGGATC	V15X_RNY
N19V_fw	GATCCTGCGTGTGGTCCTGAAACAAGG	N19V_rev	CCTTGTTTCAGGACCACACGCAGGATC	V15X_RNY
N19L_fw	GATCCTGCGTCTGGTCCTGAAACAAGG	N19L_rev	CCTTGTTTCAGGACCAGACGCAGGATC	V15X_RNY
N19I_fw	GATCCTGCGTATTGTCCTGAAACAAGG	N19I_rev	CCTTGTTTCAGGACAATACGCAGGATC	V15X_RNY
N19M_fw	GATCCTGCGTATGGTCCTGAAACAAGG	N19M_rev	CCTTGTTTCAGGACCATACGCAGGATC	V15X_RNY
N19P_fw	GATCCTGCGTCCGGTCCTGAAACAAGG	N19P_rev	CCTTGTTTCAGGACCGGACGCAGGATC	V15X_RNY
N19F_fw	GATCCTGCGTTTTGTCCTGAAACAAGG	N19F_rev	CCTTGTTTCAGGACAAAACGCAGGATC	V15X_RNY
N19Y_fw	GATCCTGCGTTATGTCCTGAAACAAGG	N19Y_rev	CCTTGTTTCAGGACATAACGCAGGATC	V15X_RNY
N19W_fw	GATCCTGCGTTGGGTCCTGAAACAAGG	N19W_rev	CCTTGTTTCAGGACCCAACGCAGGATC	V15X_RNY
N19D_fw	GATCCTGCGTGATGTCCTGAAACAAGG	N19D_rev	CCTTGTTTCAGGACATCACGCAGGATC	V15X_RNY
N19E_fw	GATCCTGCGTGAAGTCCTGAAACAAGG	N19E_rev	CCTTGTTTCAGGACTTCACGCAGGATC	V15X_RNY
N19Q_fw	GATCCTGCGTCAGGTCCTGAAACAAGG	N19Q_rev	CCTTGTTTCAGGACCTGACGCAGGATC	V15X_RNY
N19H_fw	GATCCTGCGTCATGTCCTGAAACAAGG	N19H_rev	CCTTGTTTCAGGACATGACGCAGGATC	V15X_RNY
N19K_fw	GATCCTGCGTAAAGTCCTGAAACAAGG	N19K_rev	CCTTGTTTCAGGACTTTACGCAGGATC	V15X_RNY
N19R_fw	GATCCTGCGTCGTGTCCTGAAACAAGG	N19R_rev	CCTTGTTTCAGGACACGACGCAGGATC	V15X_RNY
D100R_fw_2	CGTGTGCGTAAAATCATTGAAAATCTGGAAGC	D100R_rev_2	CAATGATTTTACGCACACGACTCCAGG	V15X_RNYW
V15Y_fw	GCTCAAACCAATTACATCCTGCGTTGGGTC	V15Y_rev	GACCCAACGCAGGATGTAATTGGTTTGAGC	V15X_RNYW
D55K_fw	CTGTATACGATTTTTAAGCGTCTGGAACAGGAC	D55K_rev	GTCCTGTTCCAGACGCTTAAAAATCGTATACAG	V15X_RNYW
Q59K_fw	GCGTCTGGAAAAGGACGGCATTATCAGC	Q59K_rev	GCTGATAATGCCGTCCTTTTCCAGACGC	V15X_RNYW _D55K

5. Determination ε_{280} 3-Aminotyrosine

The ε_{280} of free aY was determined by measuring UV-vis absorbance spectra at 280 nm. Different concentrations of aY were prepared by serial dilution in catalysis buffer (20 mM MES, 150 mM NaCl, pH 5.5) or in PBS pH 8.0. The ε_{280} of free aY under both conditions was found to be comparable (2342 M^{-1} cm^{-1} in MES pH 5.5, and 2341 M^{-1} cm^{-1} in PBS pH 8.0). The ε_{280} determined at pH 5.5 (2342 M^{-1} cm^{-1}) was used for the calculation of molar extinction coefficients for each LmrR_V15aY variant.

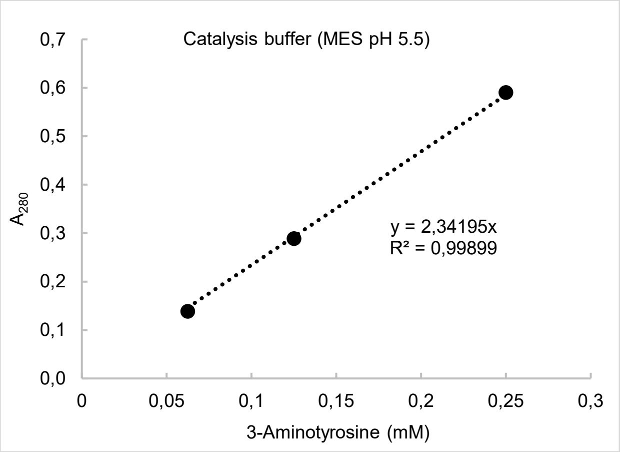

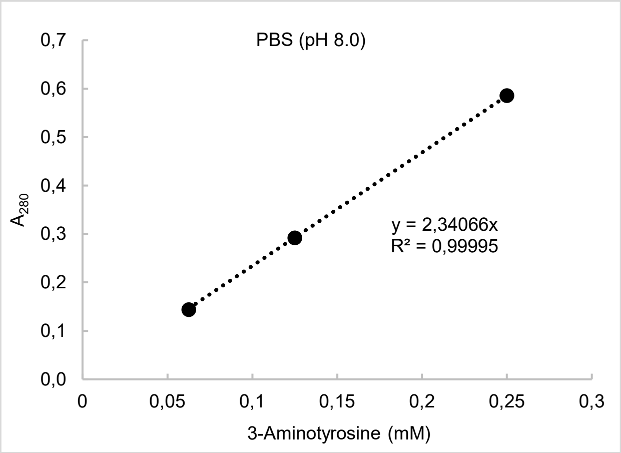

6. Protein Production and Purification

Protein expression

A tube with 5 mL LB containing ampicillin (100 µg/mL) and chloramphenicol (34 µg/mL) was inoculated from a glycerol stock of *E. coli* BL21(DE3) C41 cells harbouring a pET17b_LmrR_V15X variant and pEvol_*Mj*aYRs and incubated overnight at 37 °C in an orbital shaker. The dense pre-culture was 100-fold diluted in fresh LB containing the same antibiotics in a non-baffled Erlenmeyer flask five times the size in volume compared to the used LB (typically 100 mL of LB in a 500 mL Erlenmeyer flask). The culture was incubated at 37 °C in an orbital shaker with moderate shaking (180 rpm) until an OD_{600} of ≈ 0.8-1.0, at which point expression of LmrR and the orthogonal translation system was induced by addition of IPTG (1 mM), *L*-arabinose (0.02%) and aY (2 mM, as a solid). The culture was further incubated overnight (18-24 h) at 30 °C in an orbital shaker with moderate shaking (180 rpm). Note: cultures containing aY turned slightly brown-red overnight, and became darker brown-red coloured with longer incubation time (> 24 h). Although this was likely due to oxidation of free aY over-time, good protein yields were obtained nonetheless. After measuring the OD_{600} after expression, cells were collected by centrifugation in 50 mL centrifuge tubes (8000 xg, 10 min, 4 °C) or swing buckets (3428 xg, 45 min, 4 °C) and cell pellets frozen and stored at -21 °C. Similar procedures were followed for LmrR_V15pAF variants (using pEvol_pAzFRS.2.t1 and 1 mM of pAzF), and LmrR_WT and LmrR_V15Y variants (without pEvol plasmid, chloramphenicol and ncAA). LmrR_WT was produced in BL21(DE3) C43 and LmrR_V15Y_RGN and _RMH were produced in BL21(DE3).

Protein purification

Cell pellets were thawed and resuspended in 10-15 mL cold PBS (pH 8.0, sterilized), and EDTA (200 mM in PBS pH 8.0) was added to a final concentration of 1 mM. Resuspended cells were placed in an ice-bath and lysed by sonication (5-7 min, 5 sec on, 7 sec off, amplitude 70%). Cell extracts were centrifuged (18514 xg, 30-40 min, 4 °C) to obtain the cell-free extracts (cell lysate). Cell-free extract was passed through a 0.2 or 0.45 µm syringe filter and loaded onto a gravity column containing ≈ 3-4 mL Strep-Tactin Superflow high-capacity resin equilibrated in PBS pH 8.0. The resin was then washed with sterilized PBS pH 8.0 (4 x 5 mL) and the LmrR variant eluted with the same buffer containing 5 mM desthiobiotin (0.9 mL + 3 x 4 mL). Fractions containing protein were pooled and concentrated using a 10 kDa MWCO centrifugal filter. Purified protein was exchanged into catalysis buffer (20 mM MES, 150 mM NaCl, pH 5.5) using a PD-10 desalting column (Cytiva), frozen in liquid nitrogen and stored at -21 °C. For LmrR_V15pAF variants, prior to desalting, pAzF was reduced to pAF by adding TCEP (90 mM in PBS pH 8) to a final concentration of 10 mM, and subsequently incubated overnight at 8 °C on a turning platform. Protein concentrations were determined based on A_{280} values measured using a Nanodrop and molar extinction coefficients (ε_{280}) calculated for each LmrR variant. Extinction coefficients were approximated using the ProtParam Expasy web server https://web.expasy.org/protparam/, and corrected for the absorbance of noncanonical amino acids (ε_{280} aY = 2342 M^{-1} cm^{-1}, ε_{280} pAF = 1333 M^{-1} cm^{-1}).[4] The identity and purity of purified proteins were confirmed by MS (ESI-QTOF, see ESI section 7). For LmrR_V15aY and LmrR_V15aY_RNYW, cell extract (CE), cell-free extract (CFE) and eluted purified protein (E) were analysed by SDS-PAGE on a freshly made 12% polyacrylamide Bis-Tris gel (see **Fig. S1**).

Generally obtained yields after purification of main LmrR variants

Protein	Yield (mg/mL)
LmrR_WT*	50-70
LmrR_V15pAF	150-160
LmrR_V15aY	100-170
LmrR_V15aY_RMH	90-120
LmrR_V15aY_RGN	50-70
LmrR_V15aY_RN	80-100
LmrR_V15aY_RNY	80-100
LmrR_V15aY_RNYW	70-100

*****LmrR_WT was produced in BL21(DE3) C43. All others in BL21(DE3) C41.

Protein expression in deep well plates and preparation of cell lysates

A 24-well deep well plate with 4 mL LB containing ampicillin (100 µg/mL) and chloramphenicol (34 µg/mL) in each well was inoculated from glycerol stocks of *E. coli* BL21(DE3) C41 cells harbouring a pET17b_LmrR variant and pEvol_*Mj*aYRs, sealed with a breathable AeraSeal film (Excel Scientific), and incubated overnight at 37 °C in a plate shaker (1100 rpm). Typically one plate was inoculated with nineteen LmrR_V15X mutants, and five controls. Controls consisted of uninoculated LB, LmrR_WT (+ pEvol_*Mj*aYRs), an LmrR_V15X variant obtained in an earlier round of evolution than the current parent, and two times the parent of the current LmrR_V15X mutants. Three 24-well deep well plates containing 4.9 mL fresh LB per well with the same antibiotics were then inoculated with 200 µL dense pre-culture per well from this pre-culture plate. Locations of the different mutants and controls in the three inoculated deep well plates were altered among the three replicates to take positional variabilities into account. Deep well plates were covered with a breathable AeraSeal film and incubated at 37 °C in a plate shaker (1100 rpm). After 5-6 h of incubation ($OD_{600} \approx 3$), expression was induced by addition of 100 µL of a master mix containing IPTG, *L*-arabinose and aY in LB with antibiotics (final concentration of 1 mM, 0.02% and 2 mM, respectively), per well. Directly after induction, plates were covered with an adhesive SilverSeal foil (Greiner) and incubated overnight (16-20 h) at 30 °C in a plate shaker (1100 rpm). It was observed that cell cultures in deep well plates coloured dark brown-red after overnight incubation at 30 °C when a breathable AeraSeal was used, and that, in contrast to cell cultures in Erlenmeyer flasks, protein yields were low. Using a non-breathable aluminum SilverSeal foil to cover the plates after induction of the cells solved this problem as it prevented colouration of the growth medium and gave rise to improved protein yields, likely due to a lower degree of oxidation of the free aY. After expression, cultures of one of the three plate replicates were analysed by SDS-PAGE to assess protein expression (see **Fig**. **S7**). Plates were spun down (3220 xg, 20-30 min, 4 °C), the supernatant discarded, and cells resuspended in 4 mL of catalysis buffer (20 mM MES, 150 mM NaCl, pH 5.5). Resuspended cells were incubated for 10-20 min in a plate shaker at room temperature and subsequently spun down again. The same washing procedure was repeated one more time, followed by resuspension in 1.8 mL buffer and transfer to 2 mL microcentrifuge tubes. Cells in tubes were incubated another 10-20 minutes in a shaker at room temperature, spun down (4000 xg, 15 min, 4 °C) and the supernatant discarded. Cells were then lysed by resuspension in 350 µL lysis buffer containing protease inhibitor (Roche cOmplete EDTA-free, 1 pill / 50 mL), lysozyme (1 mg/mL). DNase I (0.1 mg/mL) and $MgCl_2$ (10 mM) in catalysis buffer (20 mM MES, 150 mM NaCl, pH 5.5), incubated for 2 h at 30 °C in tube racks in a plate shaker (1100 rpm), and subsequently frozen at -21 °C. Prior to catalysis, the cells in 2 mL microcentrifuge tubes were thawed and spun down (16000 xg, 15 min, 4 °C) to obtain the cell-free extracts (cell lysate). Cell lysates (150-276 µL) were transferred to clean 2 mL microcentrifuge tubes and used in catalysis immediately.

7. Protein Mass Spectrometry

Calculated protein masses

The protein molecular formula, obtained from the ProtParam Expasy web server, was corrected for the presence of a ncAA and used as input for an isotope simulation performed with Xcalibur Freestyle 1.8 using a profile resolution of 100000. The highest intensity isotope from this simulation was used as calculated mass (M_{calc}) for purified proteins.

Routine protein MS

Routine protein UPLC-MS (ESI-QTOF) measurements were performed for most purified proteins to verify identity and purity. Protein samples (0,05-0,15 mg/mL in 50:50 PBS pH 8.0 or catalysis buffer:MilliQ) were injected (5 µL) on an Acquity UPLC Protein BEH C4, 300 Å, 1.7 µm, 2.1 mm x 150 mm column using water (A) and acetonitrile (B) with 0.1% (v/v) formic acid as mobile phase at a flow rate of 0.3 mL/min using the following method: 90% A for 2 min, linear gradient to 50% A in 8 min, linear gradient to 5% A in 1 min, 5% A for 2 min, linear gradient back to 90% A in 0.1 min followed by 3.9 min of re-equilibration at 90% A. The total ion chromatograms obtained from mass spectrometry measurements were extracted to get the m/z spectra, which were subsequently deconvoluted using MagTran 1.0.[7] In some cases, a minor protein species was observed that corresponded to the cleavage of two C-terminal StrepTag residues (EK). This cleavage however, was not expected to have an effect on structural or functional integrity of the protein. Accuracy of the ESI-QTOF instrument varied, at times leading to higher differences between the M_{calc} and observed mass (M_{obs}). Generally, ΔM_{calc}-M_{obs} varied between 0-5 Da, which was accurate enough to confirm the identity of the different protein variants. For higher accuracy, orbitrap HRMS was performed for a selection of variants (see below, protein HRMS).

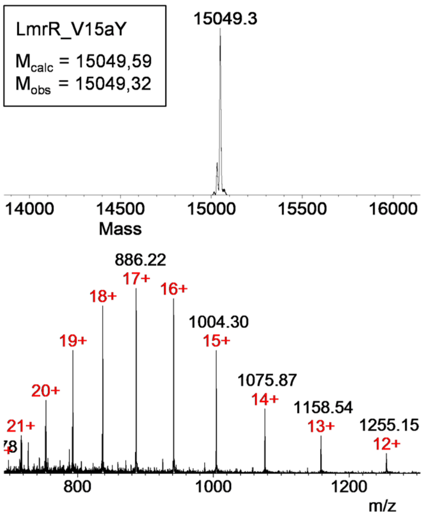

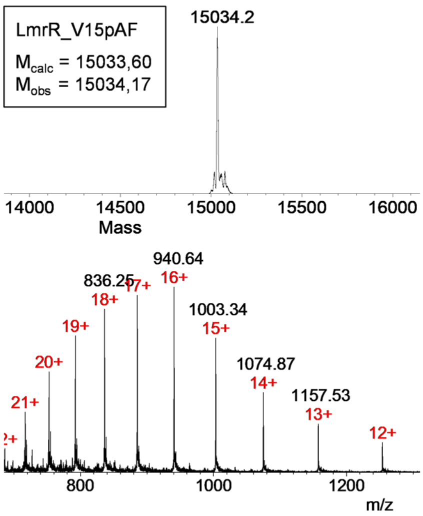

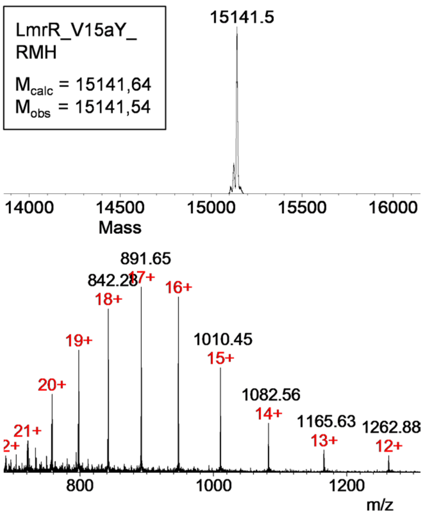

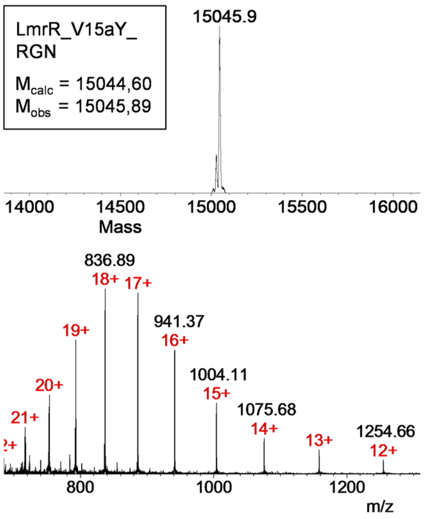

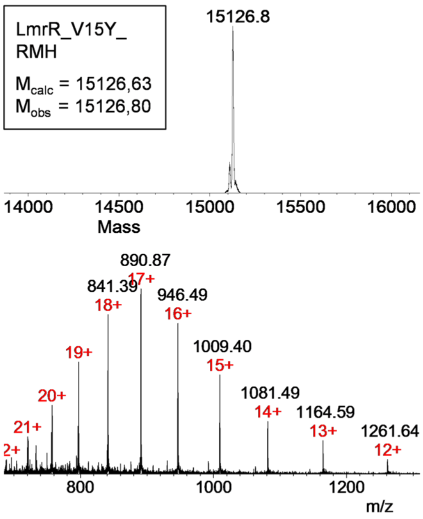

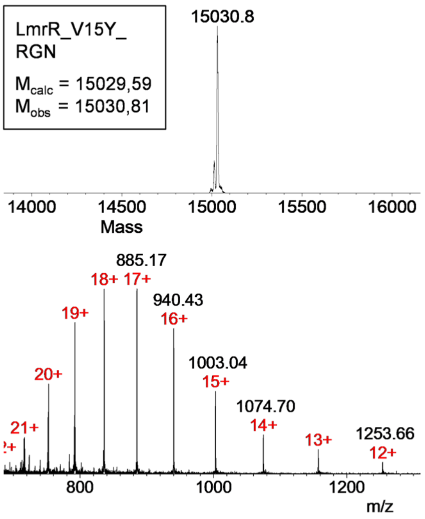

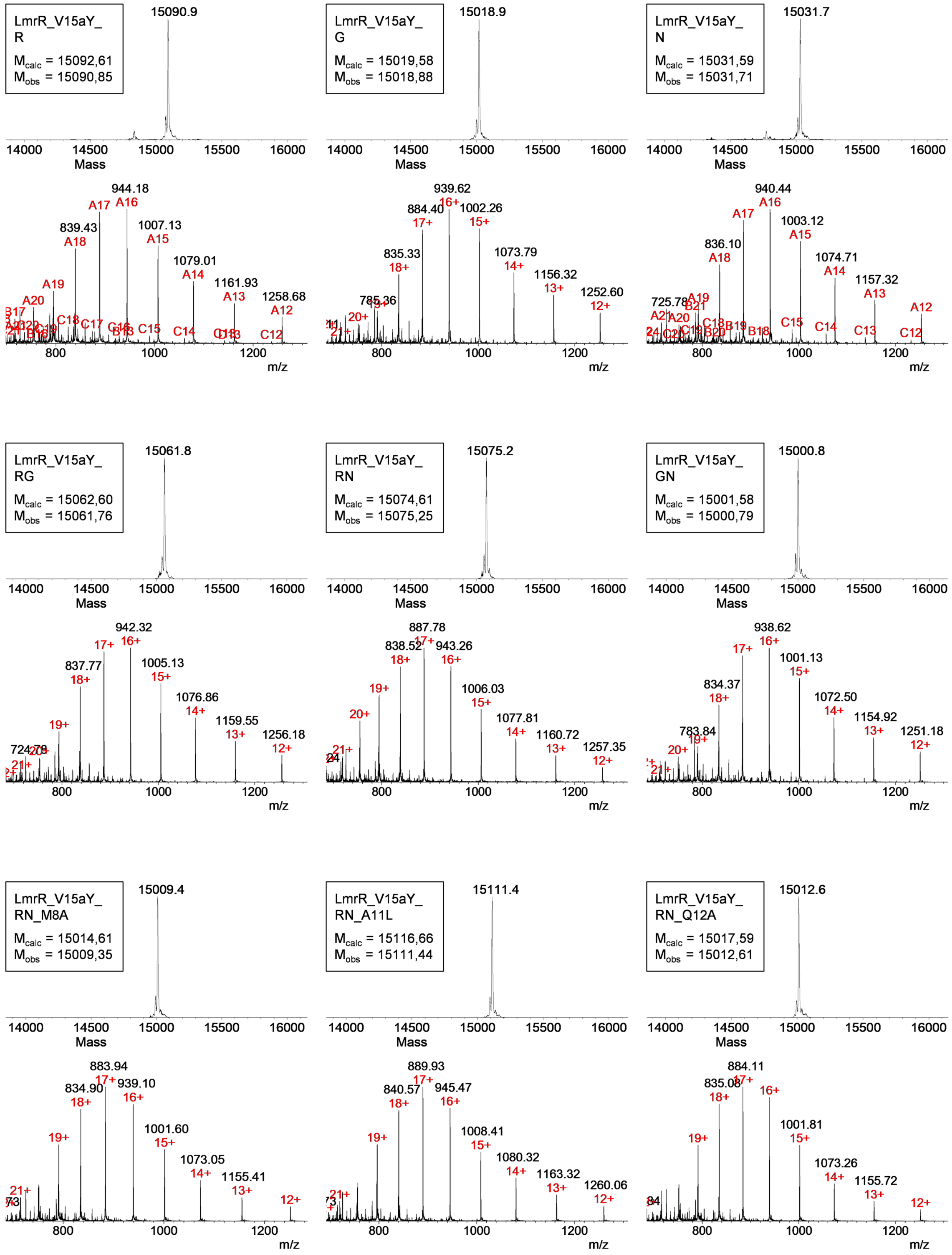
LmrR_V15aY_ R
Mcalc = 15092,61
Mobs = 15090,85
15090.9
Mass
944.18
839.43
1007.13
1079.01
1161.93
1258.68
m/z
LmrR_V15aY_ G
Mcalc = 15019,58
Mobs = 15018,88
15018.9
939.62
884.40
1002.26
835.33
1073.79
1156.32
1252.60
LmrR_V15aY_ N
Mcalc = 15031,59
Mobs = 15031,71
15031.7
940.44
1003.12
836.10
1074.71
1157.32
LmrR_V15aY_ RG
Mcalc = 15062,60
Mobs = 15061,76
15061.8
942.32
837.77
1005.13
1076.86
1159.55
1256.18
LmrR_V15aY_ RN
Mcalc = 15074,61
Mobs = 15075,25
15075.2
887.78
838.52
943.26
1006.03
1077.81
1160.72
1257.35
LmrR_V15aY_ GN
Mcalc = 15001,58
Mobs = 15000,79
15000.8
938.62
1001.13
834.37
1072.50
1154.92
1251.18
LmrR_V15aY_ RN_M8A
Mcalc = 15014,61
Mobs = 15009,35
15009.4
883.94
834.90
939.10
1001.60
1073.05
1155.41
LmrR_V15aY_ RN_A11L
Mcalc = 15116,66
Mobs = 15111,44
15111.4
889.93
840.57
945.47
1008.41
1080.32
1163.32
1260.06
LmrR_V15aY_ RN_Q12A
Mcalc = 15017,59
Mobs = 15012,61
15012.6
884.11
835.08
1001.81
1073.26
1155.72

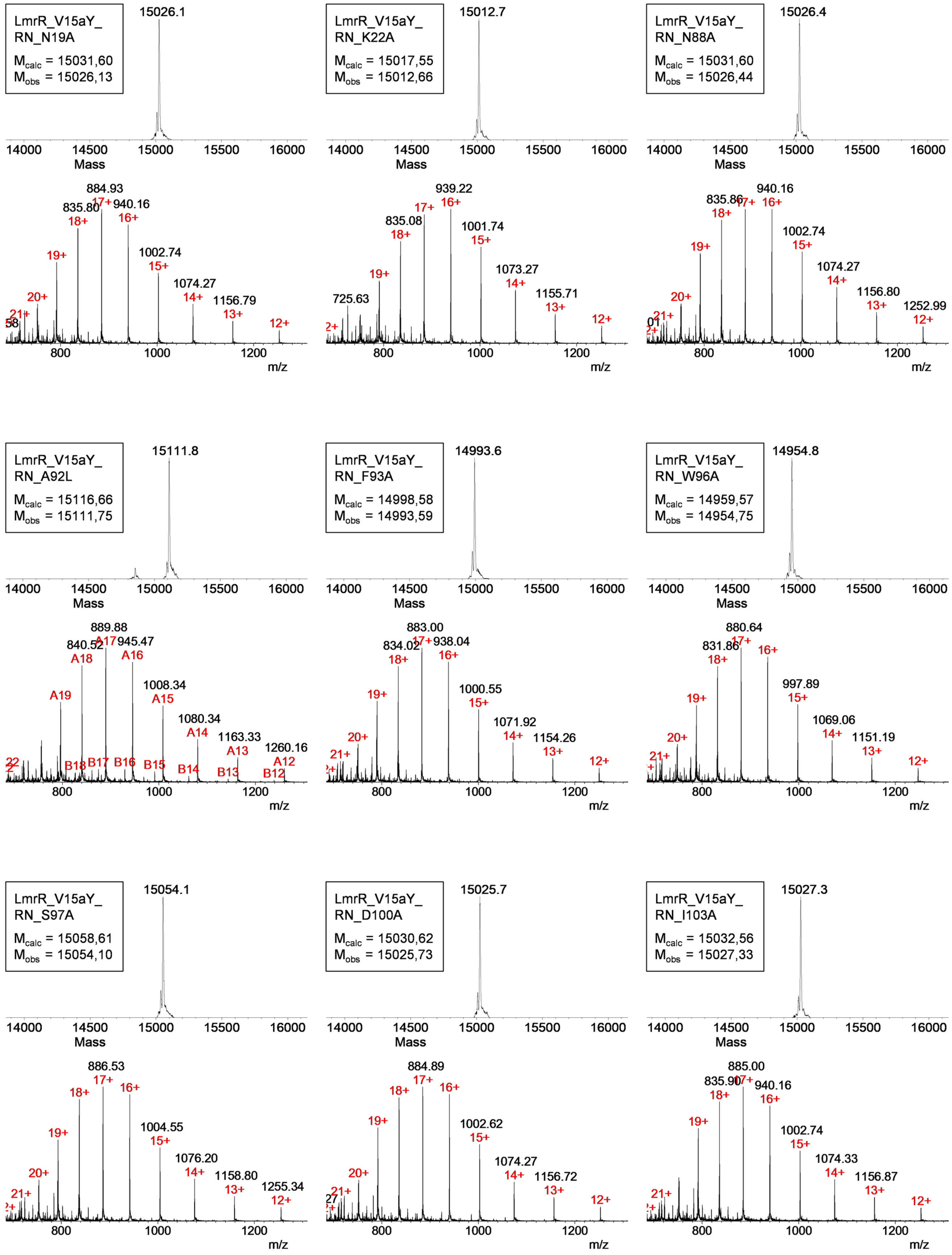
LmrR_V15aY_ RN_N19A
M_{calc} = 15031,60
M_{obs} = 15026,13
15026.1
14000 14500 15000 15500 16000
Mass
884.93 17+
835.80 18+
940.16 16+
19+
20+
21+
1002.74 15+
1074.27 14+
1156.79 13+
12+
800 1000 1200
m/z
LmrR_V15aY_ RN_K22A
M_{calc} = 15017,55
M_{obs} = 15012,66
15012.7
14000 14500 15000 15500 16000
Mass
939.22 16+
17+
835.08 18+
19+
725.63
1001.74 15+
1073.27 14+
1155.71 13+
12+
800 1000 1200
m/z
LmrR_V15aY_ RN_N88A
M_{calc} = 15031,60
M_{obs} = 15026,44
15026.4
14000 14500 15000 15500 16000
Mass
940.16 16+
835.86 18+
19+
20+
21+
1002.74 15+
1074.27 14+
1156.80 13+
1252.99 12+
800 1000 1200
m/z
LmrR_V15aY_ RN_A92L
M_{calc} = 15116,66
M_{obs} = 15111,75
15111.8
14000 14500 15000 15500 16000
Mass
889.88 A17
840.52 A18
945.47 A16
A19
1008.34 A15
1080.34 A14
1163.33 A13
1260.16 A12
B18 B17 B16 B15 B14 B13 B12
800 1000 1200
m/z
LmrR_V15aY_ RN_F93A
M_{calc} = 14998,58
M_{obs} = 14993,59
14993.6
14000 14500 15000 15500 16000
Mass
883.00 17+
834.02 18+
938.04 16+
19+
20+
21+
1000.55 15+
1071.92 14+
1154.26 13+
12+
800 1000 1200
m/z
LmrR_V15aY_ RN_W96A
M_{calc} = 14959,57
M_{obs} = 14954,75
14954.8
14000 14500 15000 15500 16000
Mass
880.64 17+
831.86 18+
16+
19+
20+
21+
997.89 15+
1069.06 14+
1151.19 13+
12+
800 1000 1200
m/z
LmrR_V15aY_ RN_S97A
M_{calc} = 15058,61
M_{obs} = 15054,10
15054.1
14000 14500 15000 15500 16000
Mass
886.53 17+
18+
16+
19+
20+
21+
1004.55 15+
1076.20 14+
1158.80 13+
1255.34 12+
800 1000 1200
m/z
LmrR_V15aY_ RN_D100A
M_{calc} = 15030,62
M_{obs} = 15025,73
15025.7
14000 14500 15000 15500 16000
Mass
884.89 17+
18+
16+
19+
20+
21+
1002.62 15+
1074.27 14+
1156.72 13+
12+
800 1000 1200
m/z
LmrR_V15aY_ RN_I103A
M_{calc} = 15032,56
M_{obs} = 15027,33
15027.3
14000 14500 15000 15500 16000
Mass
885.00 17+
835.90 18+
940.16 16+
19+
21+
1002.74 15+
1074.33 14+
1156.87 13+
12+
800 1000 1200
m/z

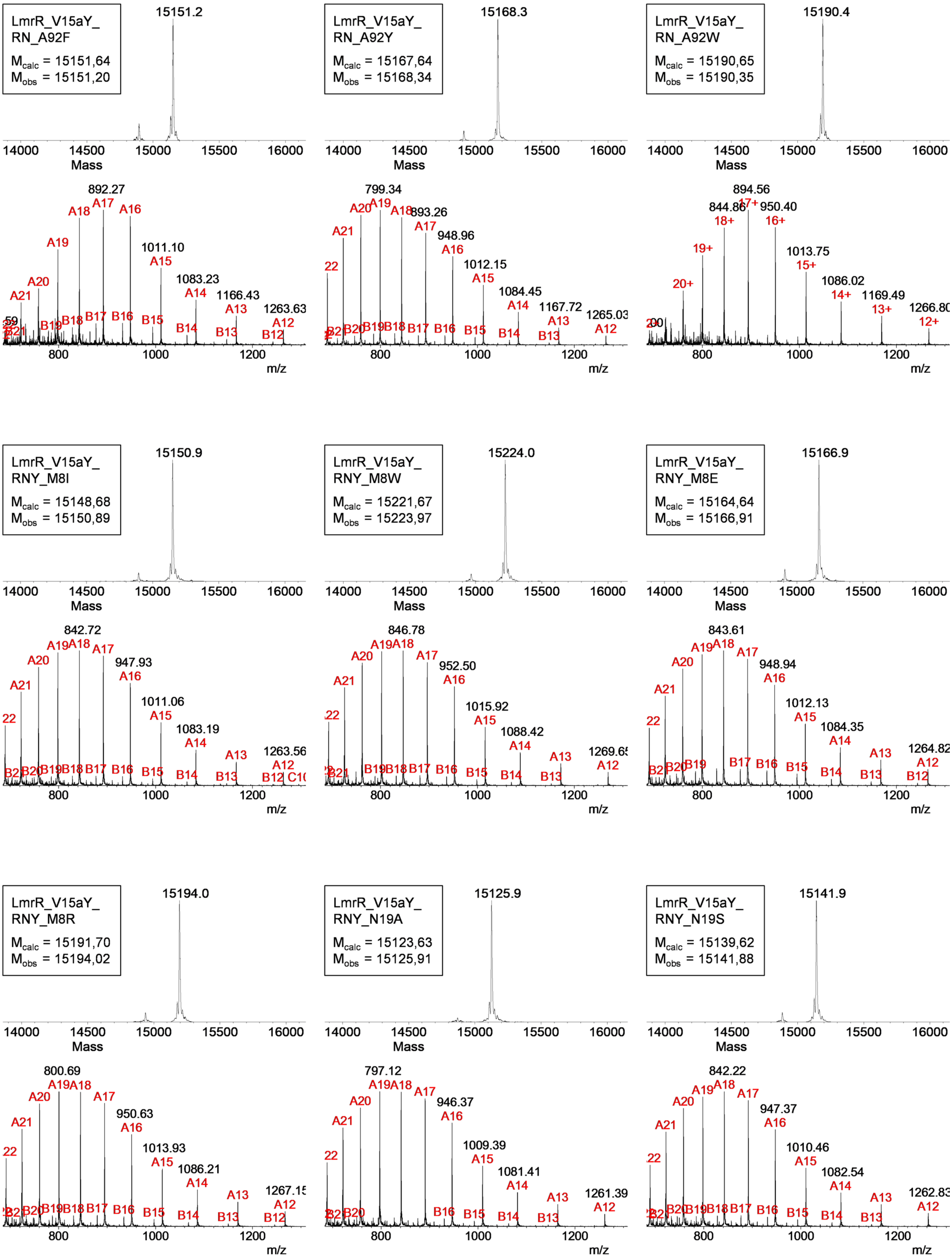
LmrR_V15aY_ RN_A92F
M_{calc} = 15151,64
M_{obs} = 15151,20
15151.2
LmrR_V15aY_ RN_A92Y
M_{calc} = 15167,64
M_{obs} = 15168,34
15168.3
LmrR_V15aY_ RN_A92W
M_{calc} = 15190,65
M_{obs} = 15190,35
15190.4
LmrR_V15aY_ RNY_M8I
M_{calc} = 15148,68
M_{obs} = 15150,89
15150.9
LmrR_V15aY_ RNY_M8W
M_{calc} = 15221,67
M_{obs} = 15223,97
15224.0
LmrR_V15aY_ RNY_M8E
M_{calc} = 15164,64
M_{obs} = 15166,91
15166.9
LmrR_V15aY_ RNY_M8R
M_{calc} = 15191,70
M_{obs} = 15194,02
15194.0
LmrR_V15aY_ RNY_N19A
M_{calc} = 15123,63
M_{obs} = 15125,91
15125.9
LmrR_V15aY_ RNY_N19S
M_{calc} = 15139,62
M_{obs} = 15141,88
15141.9
Mass
m/z

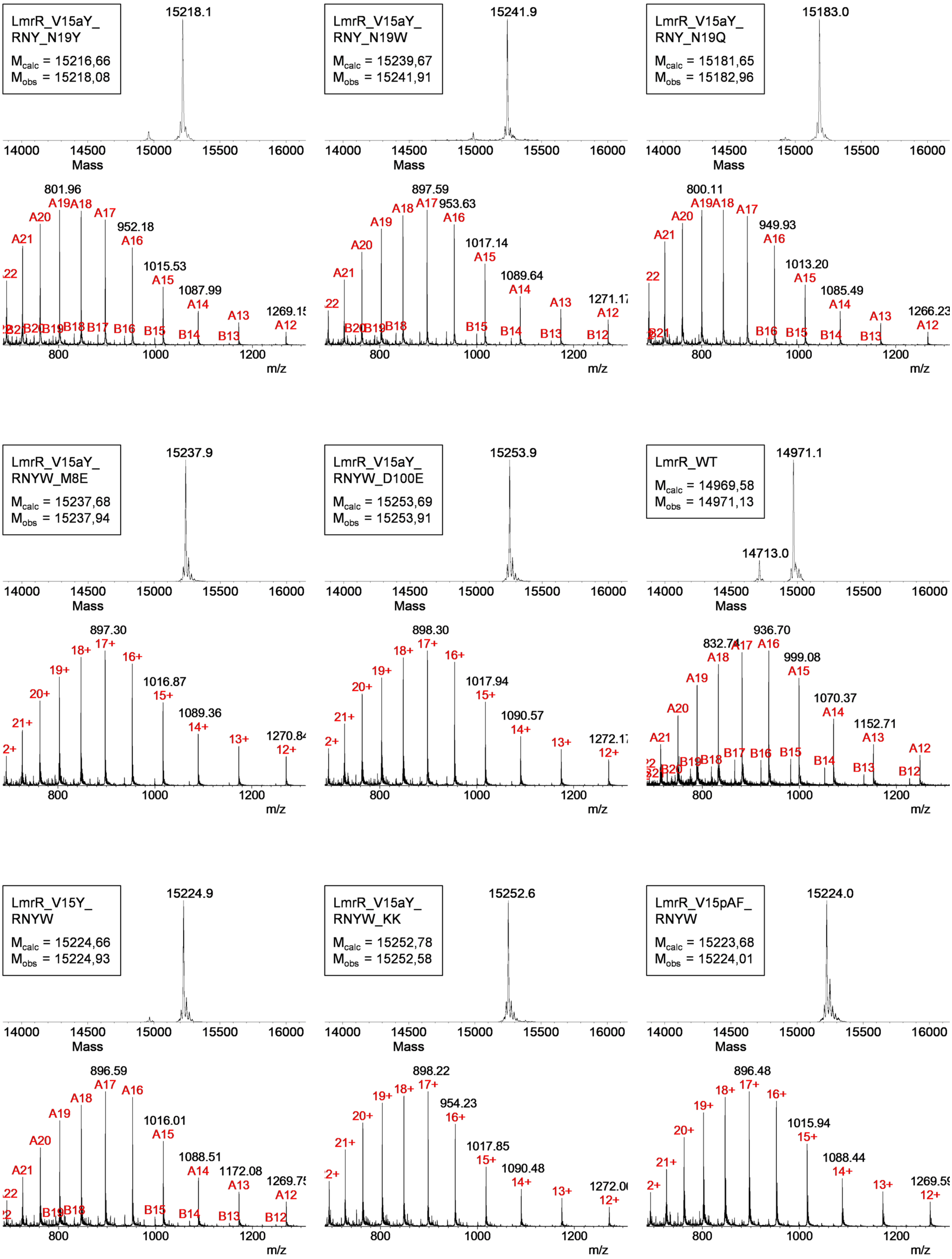
LmrR_V15aY_ RNY_N19Y
Mcalc = 15216,66
Mobs = 15218,08
15218.1
Mass
801.96
952.18
1015.53
1087.99
m/z
LmrR_V15aY_ RNY_N19W
Mcalc = 15239,67
Mobs = 15241,91
15241.9
Mass
897.59
953.63
1017.14
1089.64
m/z
LmrR_V15aY_ RNY_N19Q
Mcalc = 15181,65
Mobs = 15182,96
15183.0
Mass
800.11
949.93
1013.20
1085.49
1266.23
m/z
LmrR_V15aY_ RNYW_M8E
Mcalc = 15237,68
Mobs = 15237,94
15237.9
Mass
897.30
1016.87
1089.36
m/z
LmrR_V15aY_ RNYW_D100E
Mcalc = 15253,69
Mobs = 15253,91
15253.9
Mass
898.30
1017.94
1090.57
m/z
LmrR_WT
Mcalc = 14969,58
Mobs = 14971,13
14971.1
14713.0
Mass
936.70
999.08
1070.37
1152.71
m/z
LmrR_V15Y_ RNYW
Mcalc = 15224,66
Mobs = 15224,93
15224.9
Mass
896.59
1016.01
1088.51
1172.08
m/z
LmrR_V15aY_ RNYW_KK
Mcalc = 15252,78
Mobs = 15252,58
15252.6
Mass
898.22
954.23
1017.85
1090.48
m/z
LmrR_V15pAF_ RNYW
Mcalc = 15223,68
Mobs = 15224,01
15224.0
Mass
896.48
1015.94
1088.44
m/z

Protein HRMS selected variants

HRMS (Orbitrap Exploris 480) was performed on a selection of LmrR variants to further confirm the direct incorporation of aY *via* amber stop codon suppression with higher accuracy than compared to routine MS performed with ESI-QTOF. Protein samples (0,15 mg/mL in catalysis buffer, 5 µL injection) were separated with a bioZen 3.6 µm Intact XB-C8 column using water (A) and acetonitrile (B) with 0.1% (v/v) formic acid as mobile phase at a flow rate of 0.3 mL/min using the following method: 95% A for 2 min, linear gradient to 5% A in 10 min, 5% A for 2 min, linear gradient back to 95% A in 0.1 min. MS spectra were recorded with a scan parameter of 480000 and a scan range of 200-2000. The total ion chromatograms obtained from mass spectrometry measurements were extracted to get the m/z spectra, which were subsequently deconvoluted using MagTran 1.0.[7] Similar as observed with ESI-QTOF, in some cases, a minor protein species was observed that corresponded to the cleavage of two C-terminal StrepTag residues (EK). Zoomed-in views of the major species showed that the isotope patterns corresponded well to the predicted isotope patterns, confirming incorporation of aY. For LmrR_V15aY and LmrR_V15aY_RNYW, see **Fig. S3**.

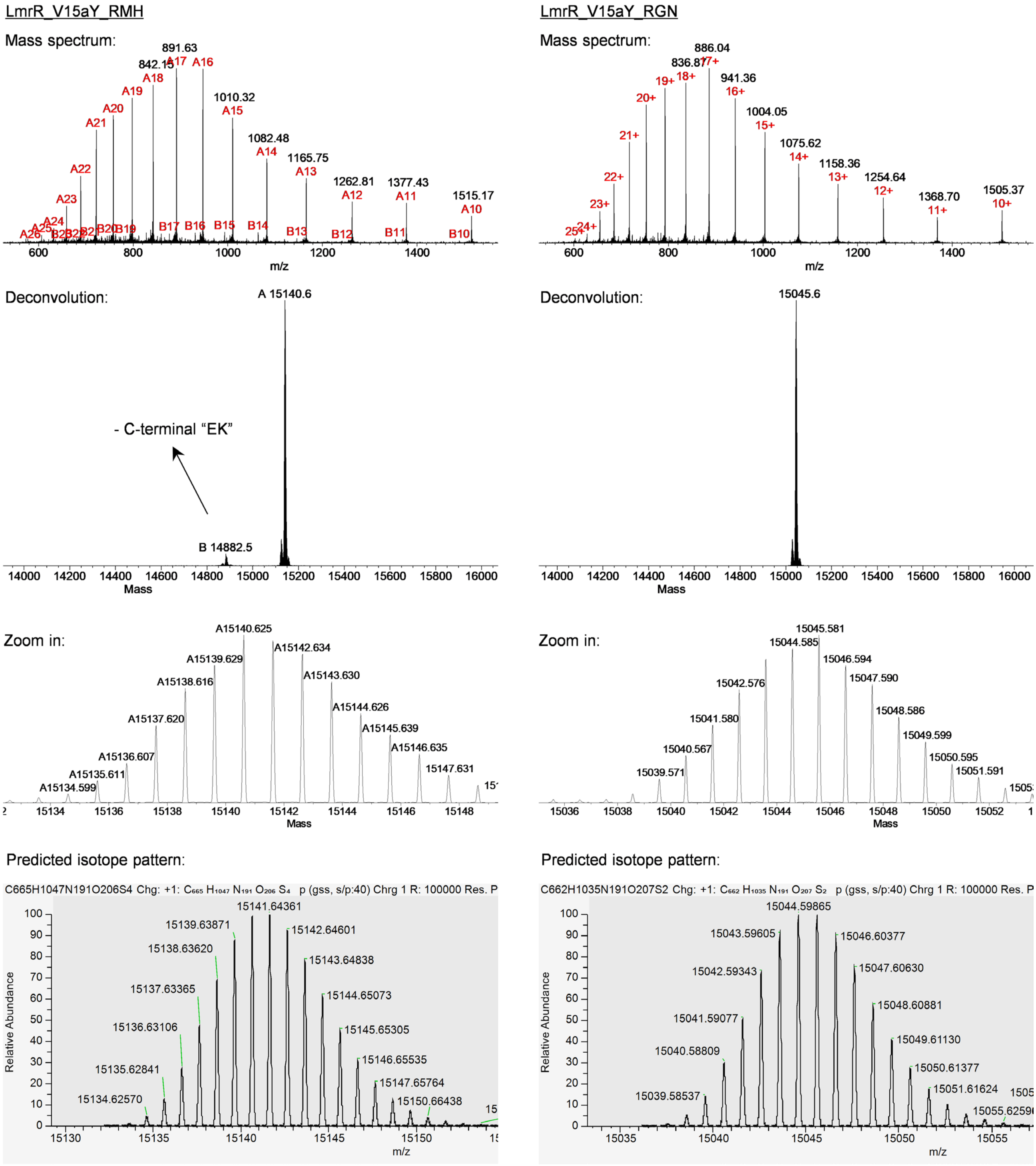

LmrR_V15aY_RN

Mass spectrum:

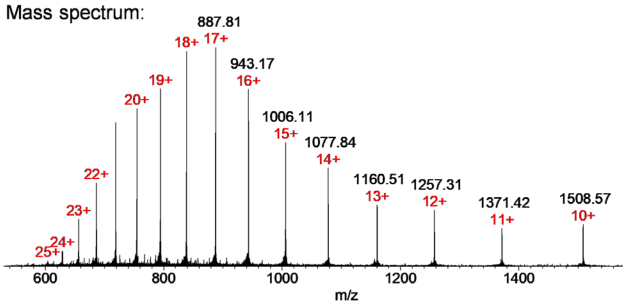

Deconvolution:

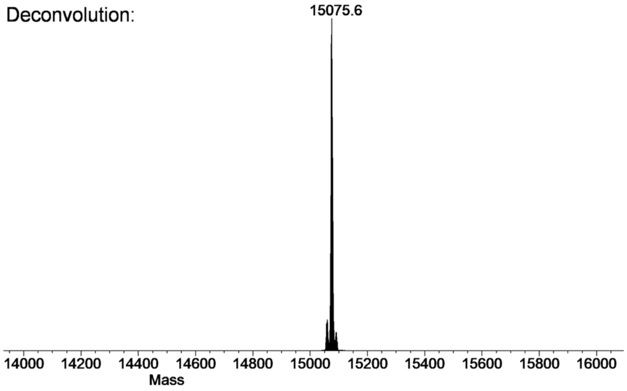

Zoom in:

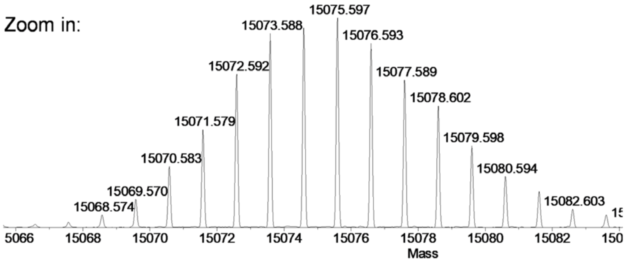

Predicted isotope pattern:

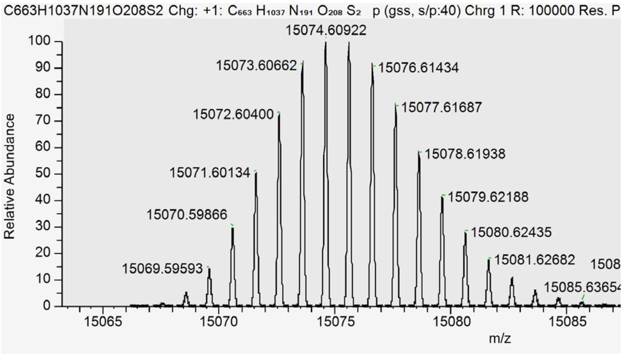

LmrR_V15aY_RNY

Mass spectrum:

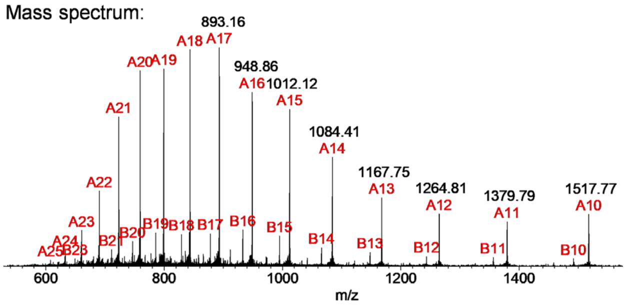

Deconvolution:

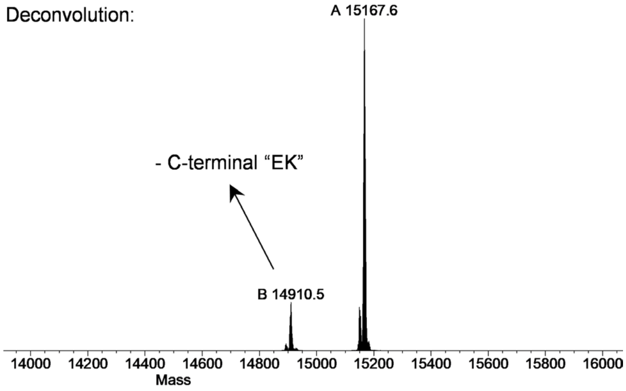

Zoom in:

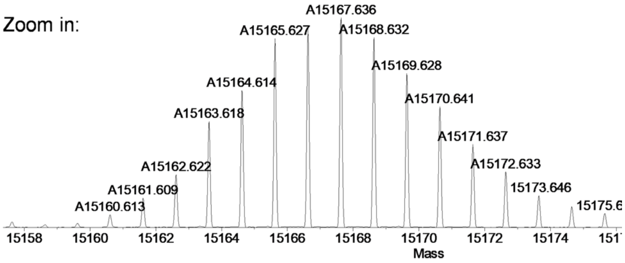

Predicted isotope pattern:

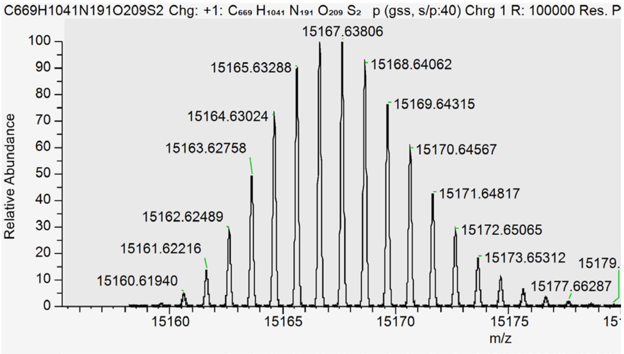

LmrR_V15Y_RNYW

Mass spectrum:

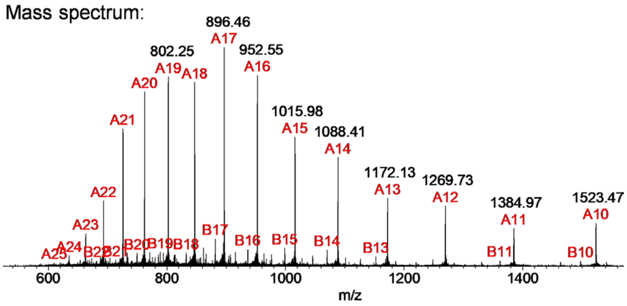

Deconvolution:

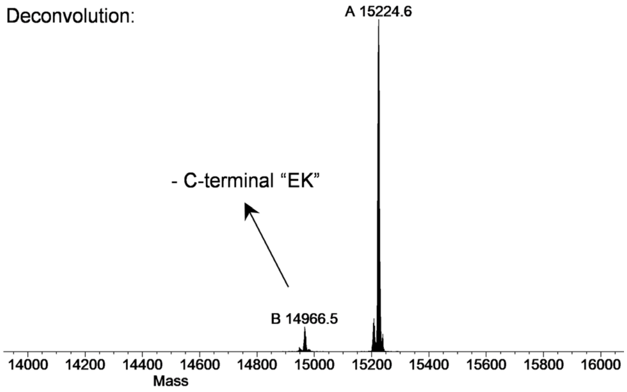

Zoom in:

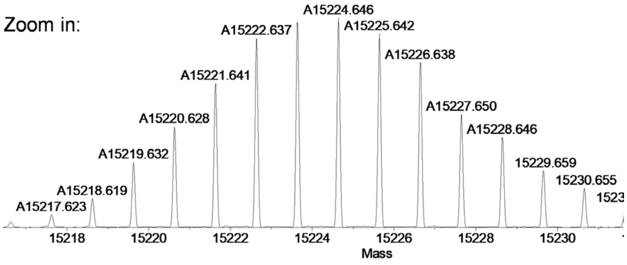

Predicted isotope pattern:

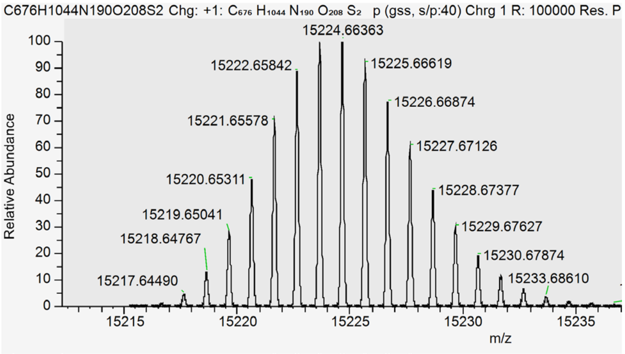

8. Protein Crystallography

Prior to crystallization, LmrR_V15aY was further purified by size exclusion chromatography on a Superdex 75 increase 10/300 GL (Cytiva) using 20 mM HEPES, 280 mM NaCl, pH 7.0 as eluent. For LmrR_V15aY_RNYW, well diffracting crystals could only be obtained by reintroducing mutations D55K and Q59K in the DNA-binding site to obtain LmrR_V15aY_RNYW_KK. For this variant, affinity chromatography was performed using buffers without NaCl, followed by further purification using a 5 mL Heparin column (Cytiva) to remove DNA, eluting the protein with 50 mM Na_2HPO_4, 1 M NaCl, pH 7.0. Size exclusion chromatography was subsequently performed as described above. Crystallization was performed with the PACT premier HT-96 and JCGS plus HT-96 screens (Molecular Dimensions) using the sitting-drop vapor diffusion method at 21 °C. Drops of 200 nL were dispensed using a Mosquito robot (SPTLabTech), mixing protein solution (7.7-16.1 mg/mL in 20 mM HEPES, 280 mM NaCl, pH 7.0) with reservoir solutions at two volume ratios (75:125 and 125:75) in MRC 96-well 2-drop crystallization plates. Crystals appeared at multiple conditions of the PACT premier suite. Crystals of LmrR_V15aY and LmrR_V15aY_RNYW_KK obtained from various conditions were cryo-protected by brief transfer to a solution similar to the respective crystallization condition with added 25% (v/v) PEG 400 or glycerol, and subsequently flash cooled in liquid nitrogen. for LmrR_V15aY, the crystal that gave the best quality X-ray diffraction data was obtained with a crystal grown at condition F4 of the PACT premier suite (0.2 M potassium thiocyanate, 0.1 M Bis-Tris propane pH 6.5, 20% (w/v) PEG 3350), using 15.8 mg/mL protein. Cryo-protection was performed with a solution containing 0.2 M potassium thiocyanate, 0.1 M Bis-Tris propane pH 7.5, 22% (w/v) PEG 3350 and 25% (v/v) glycerol. For LmrR_V15aY_RNYW_KK, the best diffracting crystal grew at condition H5 of the PACT premier suite (0.2 M sodium nitrate, 0.1 M Bis-Tris propane pH 8.5, 20% (w/v) PEG 3350), using 16.1 mg/mL protein, and was cryo-protected using a solution containing 0.1 M Bis-Tris propane pH 8.5, 280 mM NaCl, 27% (w/v) PEG 1500 and 25% (v/v) PEG 400.

X-ray diffraction data were collected at the MASSIF-1 beamline of the ESRF synchrotron, Grenoble. Data indexing and integration were initially performed using Xia2/DIALS[8,9] or XDS[10], with final scaling and merging completed *via* Aimless[11] from the CCP4 software suite[12]. Initial phases and structural models were determined by molecular replacement with PHASER[13], using a single subunit from a previously published LmrR structure (PDB: 6I8N)[1] as a search model. Structures were subsequently refined through iterative rounds of manual model building in Coot[14], interspersed with restrained refinement in REFMAC5[15]. Geometry restraints for 3-aminotyrosine (residue code TY2) were obtained from the CCP4 monomer library. The LmrR_V15aY structure (2.15 Å resolution) was refined with isotropic B-factors including TLS parameterisation, while the LmrR_V15aY_RNYW_KK structure (1.20 Å resolution) employed anisotropic B-factor refinement. Final structure validations were conducted with MolProbity[16] and the wwPDB Validation Server (https://validate.wwpdb.org). Data collection and refinement statistics are summarized in **Table S4**. Structure factors and coordinates of the refined models have been deposited in the Protein Data Bank with the accession codes 9H87 and 9H88 for LmrR_V15aY and LmrR_V15aY_RNYW_KK, respectively.

9. Catalysis and Workup Procedures

Friedel-Crafts alkylation

Reactions were performed in a total volume of 300 µL in a 2 mL microcentrifuge tube. Purified protein (desalted into catalysis buffer, generally 20 mM MES, 150 mM NaCl, pH 5.5) was added to give the specified final concentration and topped up to 276 µL with catalysis buffer. In the case of cell lysate reactions, 150-276 µL cell-free extract was used instead of purified protein. Tubes were stored on ice and 12 µL of freshly prepared β-substituted-α,β-unsaturated aldehyde stock (125 mM in DMF when using purified protein, 375 mM in DMF when using cell-free extract and 625 mM in DMF for selected scope examples, final concentrations of 5 mM, 15 mM and 25 mM, respectively) and 12 µL of freshly prepared indole substrate stock (25 mM in DMF, final concentration 1 mM) were added. Microcentrifuge tubes were then continuously inverted for the specified time at 8 °C in a cold room. Reactions performed at 25 °C were incubated in a thermomixer set at 750 rpm. Afterwards, 12 µL of internal standard stock solution (1H-indole-3-propanol, 5 mM in DMF) and 60 µL of a freshly prepared reduction solution ($NaBH_4$, 20 mg/mL in 0.5% w/v NaOH) were added and the tubes continuously inverted for another 30 min at 8 °C.

For HPLC analysis (products **3a** - **3h**), reaction mixtures were extracted with ethyl acetate (1 mL). For extraction, tubes were heavily mixed (> 1 min) using a vortex equipped with an adapter for multiple microcentrifuge tubes. Mixtures were subsequently spun down (1 min, 17000 xg) and the organic layer (900 µL) pipetted into a new microcentrifuge tube containing a small spatula tip of Na_2SO_4. Tubes were then moderately mixed on a vortex (> 1 min) and insoluble material spun down (5 min, 17000 xg). The supernatant (700 µL) was pipetted into a new microcentrifuge tube and the solvent removed in vacuo using an Eppendorf concentrator plus (45 min, 45 °C, V-HV). The obtained residue was redissolved in 100 µL of an *n*-heptane:isopropanol mixture (4:1, HPLC grade) by vortex mixing and analysed by normal-phase HPLC (20 µL injection) to determine yield and ee.

As a faster alternative to HPLC analysis, SFC analysis was performed for reactions forming product **3a**. In that case, reaction mixtures were extracted with *n*-butanol (400 µL). For extraction, tubes were heavily mixed (> 1 min) using a vortex equipped with an adapter for multiple microcentrifuge tubes. Mixtures were subsequently spun down (5 min, 17000 xg) and the organic layer (300 µL) pipetted into a new microcentrifuge tube containing a small spoon tip of Na_2SO_4. Tubes were then moderately mixed on a vortex (> 1 min) and insoluble material spun down (5 min, 17000 xg). The supernatant (150 µL) was then analysed by SFC (10 µL injection) to determine yield and ee.

Friedel-Crafts alkylation – kinetic characterization

Catalytic performance was assessed by measuring initial rates at varying concentrations of 2-methylindole (0.125-10 mM) and a constant concentration of crotonaldehyde (25 mM) using V15aY (30 µM) or V15aY_RNYW (10 µM). Saturation could not be reached when using a constant concentration of 2-methylindole (1 mM) and varying concentrations of crotonaldehyde (up to 125 mM). Reactions were setup in a total volume of 950 µL "master-mix". Purified protein (desalted into catalysis buffer, 20 mM MES, 150 mM NaCl, pH 5.5) and catalysis buffer were mixed in a total volume of 874 µL in a 2 mL microcentrifuge tube and pre-heated at 25 °C (5-10 min), after which 38 µL of a freshly prepared crotonaldehyde stock (625 mM in DMF, final concentration of 25 mM) was added. For kinetics experiments, crotonaldehyde was rapidly distilled using a Hickman distillation head prior to preparation of the stock solution in DMF. The reaction was subsequently started by addition of 38 µL of a freshly prepared 2-methylindole stock (3.125-6.25-12.5-25-50-125-200-250 mM in DMF, final concentrations of 0.125-0.25-0.5-1-2-5-8-10 mM) and the resulting "master mix" quickly mixed with a 1 mL pipette before aliquoting 3 x 300 µL in pre-heated 2 mL microcentrifuge tubes in a thermomixer (25 °C, 750 rpm). The three aliquots were quenched at different time points (between 5-60 min, depending on the 2-methylindole concentration) by addition of 60 µL of a freshly prepared reduction solution ($NaBH_4$, 20 mg/mL in 0.5% w/v NaOH) and 12 µL of internal standard stock solution (1H-indole-3-propanol, 5 mM in DMF) and the tubes subsequently continuously inverted for at least 30 min at 8 °C. Reactions were then extracted using *n*-butanol as described above and analysed by SFC. For reactions with 2-methylindole concentrations lower than 1 mM, 200-250 µL instead of 400 µL of *n*-butanol was used for extraction to increase the analyte concentration in the final SFC sample. Each reaction was conducted in triplicate using at least two independently produced batches of protein. Control reactions without protein were also performed in triplicate for each 2-methylindole concentration. Initial rates were determined using two or three of the measured time points per reaction, staying in the linear region and below 15% yield. Obtained protein-catalysed initial rates were corrected for background activity by subtraction of the average initial rate obtained from the three control replicates. Background corrected rates were fitted to the Michaelis-Menten equation using Graphpad Prism 9 to obtain the apparent catalytic parameters and are reported as a 95% confidence interval (see **Fig**. **S9**).

Friedel-Crafts alkylation using whole-cells

Unless otherwise specified, 1.2-1.6 mL (6 OD_{600} units) of cells expressing the LmrR variant (generally obtained from a 100 mL LB culture, see ESI section 6) was transferred to a 2 mL microcentrifuge tube and spun down for 5 min at 3500 xg. Cell pellets were then resuspended in catalysis buffer (20 mM MES, 150 mM NaCl, pH 5.5) and incubated slowly mixing in a shaker for 5-10 min at room temperature and then spun down again. The same washing procedure was repeated two more times and cells finally resuspended in a total volume of 276 µL catalysis buffer, and tubes stored on ice. Unless otherwise specified, 12 µL of freshly prepared β-substituted-α,β-unsaturated aldehyde stock (375 mM in DMSO, final concentration 15 mM) and 12 µL of freshly prepared indole substrate stock (25 mM in DMSO, final concentration 1 mM) were added, reaching a final concentration of whole-cells of 6 OD_{600} units/300 µL. Microcentrifuge tubes were subsequently continuously inverted for 21 h at 8 °C in a cold room. Reactions performed at 25 °C were incubated in a thermomixer set at 750 rpm. Workup and analysis of whole-cell reactions were performed the same as described above.

Upscale Friedel-Crafts alkylation using whole-cells

1000 mL of cells (OD_{600} of 2.75/mL) expressing V15aY_RNYW (obtained from 5 x 200 mL LB cultures, see ESI section 6) were spun down in two swing buckets (3428 xg, 45 min, 4 °C) and the obtained cell pellets resuspended in 450 mL catalysis buffer (20 mM MES, 150 mM NaCl, pH 5.5) each. Cells were incubated 10 min at 24 °C at 150 rpm in an orbital shaker and subsequently spun down again. This washing procedure was repeated two more times and the combined cell pellets finally resuspended in filter-sterilized catalysis buffer (126.5 mL) in a 500 mL schott bottle and cooled down in an ice-bath. A magnetic stirrer, 25 mM 2-methylindole (5.5 mL, 0.138 mmol, 1 equiv; DMSO) and 375 mM crotonaldehyde (5.5 mL, 2.063 mmol, 15 equiv; DMSO) were added sequentially, reaching a final concentration of whole cells of 6 OD_{600} units/300 µL, and the mixture left stirring (150 rpm, IKA stirrer) in a cold room (8 °C). After 16 h, the reaction mixture was filtered through a Celite plug (3 cm tall, ø = 3 cm) and washed with EtOAc (50 mL). The aqueous phase was separated and extracted with EtOAc (3x50 mL). The combined organic phases were washed with brine (2x25 mL), dried over Na_2SO_4, and concentrated under reduced pressure. The resultant yellow oil was dissolved in MeOH (1.5 mL) and $NaBH_4$ (19.5 mg, 0.516 mmol, 3.75 eq) was added at 0 °C. After 1 h at this temperature, a second portion of $NaBH_4$ (19.5 mg, 0.516 mmol, 3.75 eq) was added. After a total of 2.5 h at 0 °C, aqueous saturated $NaHCO_3$ (3 mL) was added and the reaction mixture was left under stirring at room temperature. After 30 min, the aqueous phase was separated and extracted with EtOAc (3x5 mL). The combined organic phases were washed with brine (1x5 mL), dried over Na_2SO_4, and concentrated under reduced pressure. The crude mixture was purified by flash column chromatography (SiO_2, 0 to 10% DCM/pentane, 0.1% Et_3N) to yield **3a** (13.8 mg, 67.9 µmol; 49% yield) as a thick, yellow oil.

10. Preparation and Characterization of Reference Products

General procedure for the synthesis of reference products

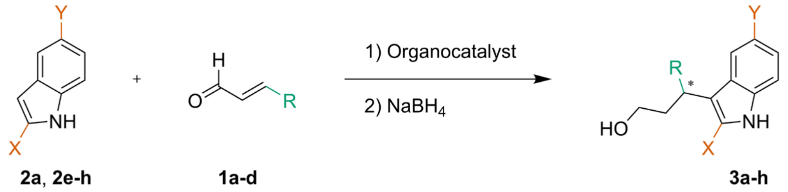

To a stirred solution of indole substrate (**2a**, **2e-h**) (1.0 equiv), (+)-2-*tert*-butyl-3-methyl-5-benzyl-4-imidazolidinone (0.05 equiv) and (−)-2-*tert*-butyl-3-methyl-5-benzyl-4-imidazolidinone (0.05 equiv) in MeOH (0.4 M) in a resealable pressure tube was added β-substituted-α,β-unsaturated aldehyde substrate (**1a-d)** (1.5-3.0 equiv) at room temperature. The reaction vessel was sealed and put at 50 °C in a fitted aluminium heating block. After 12-16 h, $NaBH_4$ (5.0 equiv) was added portion wise at 0 °C. After stirring 30 min at room temperature, the reaction mixture was quenched by addition of aq. sat. $NaHCO_3$ solution, extracted with EtOAc, dried over Na_2SO_4 and filtered. The organic solvents were removed under reduced pressure. The resultant residue was purified by flash column chromatography (SiO_2, 2 to 10% EtOAc/DCM, 0.1% Et_3N) to furnish compounds **3a**-**3h**. Preparative TLCs were performed when necessary to yield the respective compounds in higher purity. *Observation*: the products are acid labile and can show colouration and degradation when in contact with SiO_2 in the absence of Et_3N, or when using $CDCl_3$ without previous basic treatment.

Reference product 3a (3-(2-methyl-1H-indol-3-yl)butan-1-ol)

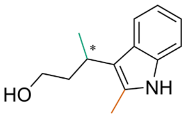

Compound **3a** (44 mg, 0.22 mmol; 54% yield) was obtained as a yellow gum from 2-methylindole (**2a**, 53 mg, 0.40 mmol, 1.0 equiv) and crotonaldehyde (**1a**, 99 µL, 1.2 mmol, 3.0 equiv; mixture of *cis* and *trans* ~1:20) following the general procedure. The spectroscopic data were consistent with those reported in the literature.[17] **^{1}H NMR** (400 MHz, $CDCl_3$) δ 7.72 (br. s., 1 H), 7.62 (d, *J* = 7.9 Hz, 1 H), 7.30 - 7.26 (m, 1 H), 7.10 (br. t, *J* = 7.5 Hz, 1 H), 7.05 (br. t, *J* = 7.2 Hz, 1 H), 3.59 (td, *J* = 5.8, 10.2 Hz, 1 H), 3.51 (td, *J* = 6.5, 11.0 Hz, 1 H), 3.22 - 3.14 (m, 1 H), 2.40 (s, 3 H), 2.20 - 2.09 (m, 1 H), 2.07 - 1.95 (m, 1 H), 1.44 (d, *J* = 7.2 Hz, 3 H). **^{13}C NMR** (101 MHz, $CDCl_3$) δ 135.5, 130.3, 127.2, 120.7, 119.2, 118.9, 115.3, 110.3, 62.0, 39.4, 27.8, 21.4, 12.1. **HRMS** (ESI-MS) m/z: $[M+H]^+$ calcd for $C_{13}H_{18}NO$ 204.1383, found 204.1382. **HPLC** Chiralpak AS-H (*n*-heptane:iPrOH 90:10, 0.5 mL/min), t_R = 12.7-13.0 min; 14.0-14.4 min. **SFC** Chiracel OJ-3 ($scCO_2$ (A) and MeOH (B) as mobile phase, linear gradient from 3% B to 50% B in 4.5 min, followed by 50% B for 2 min, 1.8 mL/min), t_R = 2.7 min; 2.9 min.

Reference product 3b (3-(2-methyl-1H-indol-3-yl)hexan-1-ol)

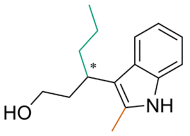

Compound **3b** (54 mg, 0.23 mmol; 58% yield) was obtained as a yellow gum from **2a** (53 mg, 0.40 mmol, 1.0 equiv) and (*E*)-2-hexenal (**1b**, 0.14 mL, 1.20 mmol, 3.0 equiv) following the general procedure. The spectroscopic data were consistent with those reported in the literature.[5] **^{1}H NMR** (400 MHz, $CDCl_3$) δ 7.73 (br. s., 1 H), 7.60 (d, *J* = 7.9 Hz, 1 H), 7.29 - 7.27 (m, 1 H), 7.10 (t, *J* = 7.5 Hz, 1 H), 7.03 (t, *J* = 7.5 Hz, 1 H), 3.56 (td, *J* = 5.8, 10.6 Hz, 1 H), 3.51 - 3.43 (m, 1 H), 2.99 (tt, *J* = 5.0, 10.2 Hz, 1 H), 2.39 (s, 3 H), 2.20 - 2.09 (m, 1 H), 2.07 - 1.97 (m, 1 H), 1.96 - 1.86 (m, 1 H), 1.76 - 1.65 (m, 1 H), 1.25 - 1.15 (m, 2 H), 0.85 (t, *J* = 7.4 Hz, 3 H). **^{13}C NMR** (151 MHz, $CDCl_3$) δ 135.6, 131.3, 127.4, 120.6, 119.3, 118.7, 113.6, 110.3, 61.9, 38.1, 37.8, 33.4, 21.2, 14.1, 12.1. **HRMS** (ESI-MS) m/z: $[M+H]^+$ calcd for $C_{15}H_{22}NO$ 232.1696, found 232.1697. **HPLC** Luxcellulose-3 (Chiralcel OJ-H) (80:20 *n*-heptane:iPrOH, 1 mL/min), t_R = 5.9 min; 6.7 min.

Reference product 3c (4-methyl-3-(2-methyl-1H-indol-3-yl)pentan-1-ol)

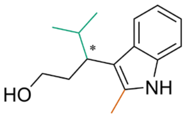

Compound **3c** (41 mg, 0.18 mmol; 59% yield) was obtained as a colourless gum from **2a** (39 mg, 0.30 mmol, 1.0 equiv) and (*E*)-4-methylpent-2-enal (**1c**, 52 µL, 0.45 mmol, 1.5 equiv) following the general procedure. The spectroscopic data were consistent with those reported in the literature.[5] **^{1}H NMR** (400 MHz, $CDCl_3$) δ 7.79 (br. s., 1 H), 7.59 (d, *J* = 7.9 Hz, 1 H), 7.27 (d, *J* = 7.9 Hz, 1 H), 7.11 (t, *J* = 7.3 Hz, 1 H), 7.04 (t, *J* = 7.5 Hz, 1 H), 3.49 (td, *J* = 6.1, 10.6 Hz, 1 H), 3.39 (td, *J* = 7.2, 10.6 Hz, 1 H), 2.58 (dt, *J* = 4.4, 10.2 Hz, 1 H), 2.36 (s, 3 H), 2.23 - 2.06 (m, 3 H), 1.10 (d, *J* = 6.5 Hz, 3 H), 0.74 (d, *J* = 6.5 Hz, 3 H). **^{13}C NMR** (101 MHz, $CDCl_3$) δ 135.5, 131.6, 127.6, 120.6, 119.5, 118.7, 113.2, 110.3, 62.3, 41.2, 34.7, 32.6, 21.8, 21.6, 12.2. **HRMS** (ESI-MS) m/z: $[M+H]^+$ calcd for $C_{15}H_{22}NO$ 232.1696, found 232.1695. **HPLC** Chiralpak AS-H (90:10 *n*-heptane:iPrOH, 0.5 mL/min), t_R = 11.1 min; 12.8 min.

Reference product 3d (3-(2-methyl-1H-indol-3-yl)-3-phenylpropan-1-ol)

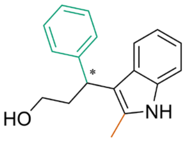

Compound **3d** (22 mg, 83 µmol; 17% yield) was obtained as a yellow gum from **2a** (66 mg, 0.50 mmol, 1.0 equiv) and (*E*)-cinnamaldehyde (**1d**, 94 µL, 0.75 mmol, 1.5 equiv) following the general procedure. The spectroscopic data were consistent with those reported in the literature.[5] **^{1}H NMR** (400 MHz, $CDCl_3$) δ 7.83 (br. s., 1 H), 7.52 (d, *J* = 7.9 Hz, 1 H), 7.40 - 7.36 (m, 2 H), 7.30 - 7.24 (m, 3 H), 7.16 (br. t, *J* = 7.3 Hz, 1 H), 7.10 (br. t, *J* = 7.8 Hz, 1 H), 7.01 (br. t, *J* = 7.5 Hz, 1 H), 4.44 (t, *J* = 8.0 Hz, 1 H), 3.68 (td, *J* = 6.1, 10.6 Hz, 1 H), 3.59 (td, *J* = 6.8, 10.6 Hz, 1 H), 2.57 - 2.50 (m, 2 H), 2.40 (s, 3 H). **^{13}C NMR** (101 MHz, $CDCl_3$) δ 145.0, 135.4, 131.6, 128.2, 127.7, 127.6, 125.7, 120.8, 119.3, 119.2, 113.3, 110.3, 61.6, 38.0, 36.8, 12.2. **HRMS** (ESI-MS) m/z: $[M+H]^-$ calcd for $C_{18}H_{18}NO$ 264.1394, found 264.1396. **HPLC** Luxcellulose-1 (Chiralcel OD-H) (85:15 *n*-heptane:iPrOH, 1 mL/min), t_R = 25.8-26.0 min; 28.3-28.9 min.

Reference product 3e (3-(1H-indol-3-yl)butan-1-ol)

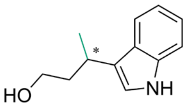

Compound **3e** (31 mg, 0.17 mmol; 55% yield) was obtained as a yellow gum from indole (**2e**, 35 mg, 0.30 mmol, 1.0 equiv) and **1a** (75 µL, 0.90 mmol, 3.0 equiv) following the general Procedure. The spectroscopic data were consistent with those reported in the literature.[18] **^{1}H NMR** (400 MHz, $CDCl_3$) δ 7.97 (br. s., 1 H), 7.68 (d, *J* = 7.9 Hz, 1 H), 7.37 (d, *J* = 8.2 Hz, 1 H), 7.20 (t, *J* = 7.3 Hz, 1 H), 7.12 (t, *J* = 7.2 Hz, 1 H), 7.00 (s, 1 H), 3.74 - 3.63 (m, 2 H), 3.25 (sxt, *J* = 7.2 Hz, 1 H), 2.14 - 2.02 (m, 1 H), 1.95 (qd, *J* = 6.7, 13.4 Hz, 1 H), 1.45 (br. s., 1 H), 1.40 (s, 3 H). **^{13}C NMR** (101 MHz, $CDCl_3$) δ 136.5, 126.6, 121.9, 121.6, 120.1, 119.3, 119.1, 111.2, 61.5, 40.3, 27.7, 21.7. **HRMS** (ESI-MS) m/z: $[M-H]^-$ calcd for $C_{12}H_{14}NO$ 188.1081, found 188.1083. **HPLC** Luxcellulose-1 (Chiralcel OD-H) (85:15 *n*-heptane:iPrOH, 1 mL/min), t_R = 14.4 min (*R*); 16.0 min (*S*). **HPLC** Luxcellulose-1 (Chiralcel OD-H) (90:10 *n*-hexane:EtOH, 1 mL/min), t_R = 16.1 min (*R*); 17.8 min (*S*). Absolute configuration of products was assigned by comparison of order of elution with the literature and previous work (also see ESI section 11).[5,19]

Reference product 3f (3-(5-methyl-1H-indol-3-yl)butan-1-ol)

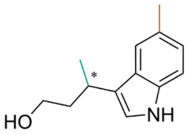

Compound **3f** (33 mg, 0.16 mmol; 54% yield) was obtained as a colourless gum from 5-methylindole (**2f**, 39 mg, 0.30 mmol, 1.0 equiv) and **1a** (75 µL, 0.90 mmol, 3.0 equiv) following the general procedure. The spectroscopic data were consistent with those reported in the literature.[20] **^{1}H NMR** (400 MHz, $CDCl_3$) δ 7.92 (br. s., 1 H), 7.46 (s, 1 H), 7.26 (d, *J* = 9.2 Hz, 1 H), 7.03 (d, *J* = 8.2 Hz, 1 H), 6.95 (s, 1 H), 3.75 - 3.63 (m, 2 H), 3.21 (qd, *J* = 7.1, 14.2 Hz, 1 H), 2.48 (s, 3 H), 2.08 (qd, *J* = 6.9, 13.5 Hz, 1 H), 1.95 (qd, *J* = 6.7, 13.4 Hz, 1 H), 1.40 (d, *J* = 6.8 Hz, 3 H). **^{13}C NMR** (101 MHz, $CDCl_3$) δ 135.9, 128.5, 128.0, 124.1, 121.6, 120.6, 119.9, 111.6, 61.7, 41.2, 28.3, 22.4, 22.2. **HRMS** (ESI-MS) m/z: $[M-H]^-$ calcd for $C_{13}H_{16}NO$ 202.1237, found 202.1238. **HPLC** Luxcellulose-3 (Chiralcel OJ-H) (80:20 *n*-heptane:iPrOH, 0.5 mL/min), t_R = 18.0 min; 23.0 min.

Reference product 3g (3-(5-methoxy-1H-indol-3-yl)butan-1-ol)

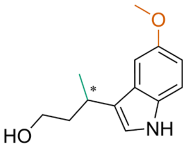

Compound **3g** (24 mg, 0.11 mmol; 37% yield) was obtained as a yellow gum from 5-methoxyindole (**2f**, 44 mg, 0.30 mmol, 1.0 equiv) and **1a** (75 µL, 0.90 mmol, 3.0 equiv) following the general procedure. The spectroscopic data were consistent with those reported in the literature.[18] **^{1}H NMR** (400 MHz, $CDCl_3$) δ 7.89 (br. s., 1 H), 7.28 - 7.23 (m, 1 H), 7.12 (br. s, 1 H), 6.98 (br. s, 1 H), 6.87 (br. d, *J* = 8.9 Hz, 1 H), 3.88 (s, 3 H), 3.76 - 3.64 (m, 2 H), 3.21 (qd, *J* = 6.9, 14.0 Hz, 1 H), 2.05 (br. qd, *J* = 7.0, 13.7 Hz, 1 H), 1.94 (br. qd, *J* = 6.5, 13.5 Hz, 1 H), 1.40 (d, *J* = 7.2 Hz, 3 H). **^{13}C NMR** (101 MHz, $CDCl_3$) δ 153.7, 131.7, 127.0, 121.4, 120.9, 112.0, 111.8, 101.4, 61.5, 56.0, 40.2, 27.6, 21.7. **HRMS** (ESI-MS) m/z: $[M-H]^-$ calcd for $C_{13}H_{16}NO_2$ 218.1187, found 218.1189. **HPLC** Luxcellulose-3 (Chiralcel OJ-H) (85:15 *n*-heptane:iPrOH, 1 mL/min), t_R = 16.6 min; 18.6 min.

Reference product 3h (3-(5-bromo-1H-indol-3-yl)butan-1-ol)

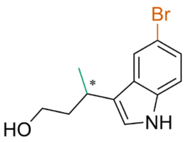

Compound **3h** (61 mg, 0.23 mmol; 76% yield) was obtained as a yellow gum from 5-bromoindole (**2h**, 59 mg, 0.30 mmol, 1.0 equiv) and **1a** (75 µL, 0.90 mmol, 3.0 equiv) following the general procedure. The spectroscopic data were consistent with those reported in the literature.[18] **^{1}H NMR** (600 MHz, $CDCl_3$) δ 8.10 (br. s, 1H), 7.78 (d, *J* = 1.8 Hz, 1H), 7.26 (dd, *J* = 8.6, 1.9 Hz, 1H), 7.21 (d, *J* = 8.6 Hz, 1H), 6.98 (d, *J* = 2.4 Hz, 1H), 3.66 (qt, *J* = 10.6, 6.6 Hz, 2H), 3.17 (hept, *J* = 7.1 Hz, 1H), 2.02 (ddt, *J* = 14.2, 8.0, 6.4 Hz, 1H), 1.91 (dq, *J* = 13.4, 6.7 Hz, 1H), 1.37 (d, *J* = 7.0 Hz, 3H). **^{13}C NMR** (151 MHz, $CDCl_3$) δ 135.1, 128.4, 124.7, 121.9, 121.4, 121.4, 112.6, 112.4, 61.4, 40.1, 27.5, 21.6. **HRMS** (ESI-MS) m/z: $[M-H]^-$ calcd for $C_{12}H_{13}BrNO$ 266.0186, found 266.0186. **HPLC** Luxcellulose-3 (Chiralcel OJ-H) (85:15 *n*-heptane:iPrOH, 1 mL/min), t_R = 9.9 min; 11.2 min.

11. Calibration Curves and HPLC / SFC chromatograms

Calibration curves were made using synthesized reference compounds (see ESI section 10). Reference products were thoroughly dried under vacuum before sample preparation. Samples were prepared the same as when setting up the Friedel-Crafts alkylation reactions, but without protein and with 0.1-1.1 mM of reference product instead of substrates. Workup and analysis were then done the same as would be done for catalysis reactions. Calibration entries are an average of two independent experiments and errors bars are the standard deviation of the results. Retention times sometimes slightly shifted over time, but authenticity of the products, substrates and internal standard was confirmed by their UV-vis absorbance. For each of these, their general retention time and characteristic maximum absorbance (λ_{max}, not taking into account absorbance < 220 nm) are stated. For **3a**-**3d** and **3f**-**3h**, ee is assigned relative to V15aY, in which the "+" symbol represents the enantiomer obtained with V15aY and the "–" symbol represents the opposite enantiomer. For **3a**-**3c** and **3e**, opposite enantiomers of the products were obtained compared to previous work using V15pAF.[5] Absolute configuration of **3e** was assigned by using the same separation method as reported in literature (literature reported method: Chiracel OD−H and OD guard column, 90:10 hexanes:EtOH, 1 mL/min, (*R*) = 17.6 min and (*S*) = 20.2 min)[19], and comparing the order of elution of products (method used in this work: Luxcellulose-1 (equivalent to Chiralcel OD-H), 90:10 *n*-hexane:EtOH, 1 mL/min, (*R*) = 16.1 min and (*S*) = 17.8 min). Following the order of elution, the major enantiomer obtained in catalysis using V15aY is (*R*). Moreover, using the same separation method as used in previous work for V15pAF (85:15 *n*-heptane:iPrOH)[5] it was further demonstrated that the major enantiomer for **3e** obtained in catalysis using V15aY (*R*) is opposite compared to when V15pAF was used (*S*).

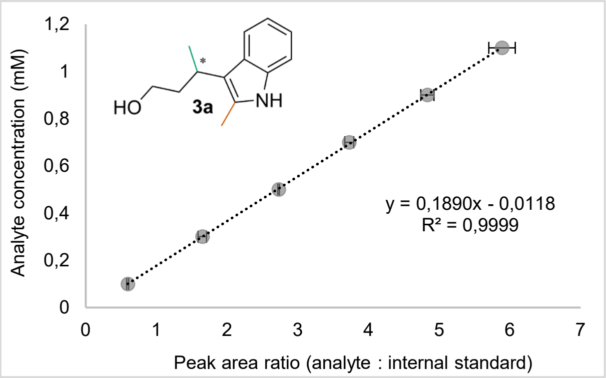

<u>HPLC – Product **3a** (3-(2-methyl-1H-indol-3-yl)butan-1-ol)</u>

HPLC method: Chiralpak AS-H, 90:10 *n*-heptane:iPrOH, 0.5 mL/min, 30 min, 20 µL injection, integrated at 282 nm.

2a: 9.4-9.5 min (λ_{max} 268 nm)
3a "–": 12.7-13.0 min (λ_{max} 282 nm)
3a "+": 14.0-14.4 min (λ_{max} 282 nm)
IS: 18.8-19.3 min (λ_{max} 281 nm)

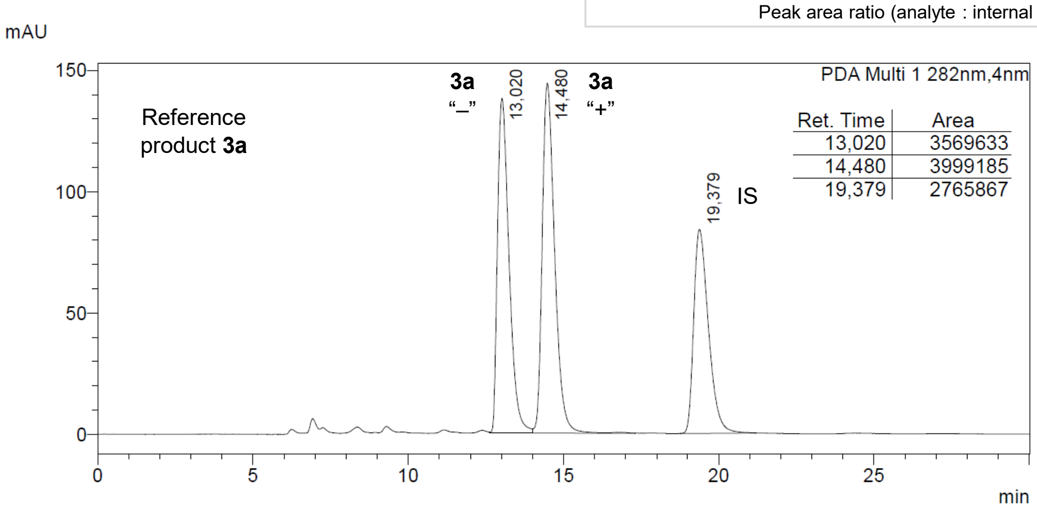

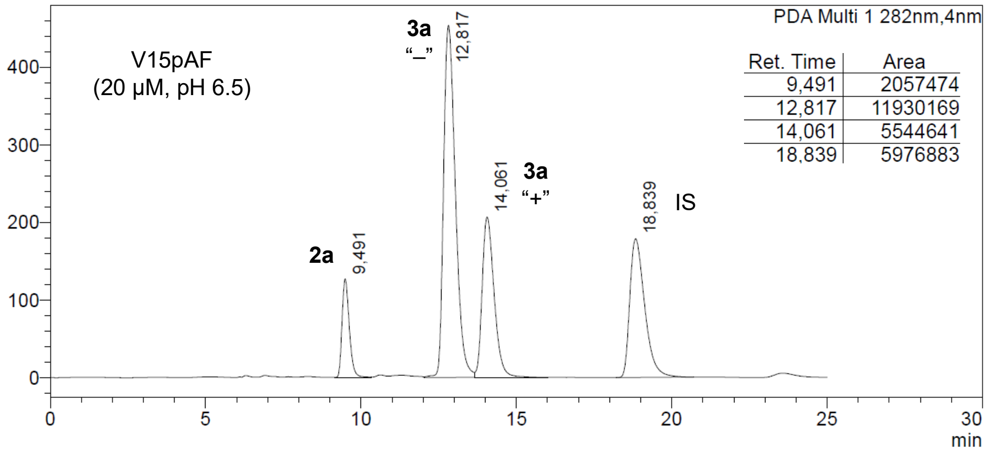

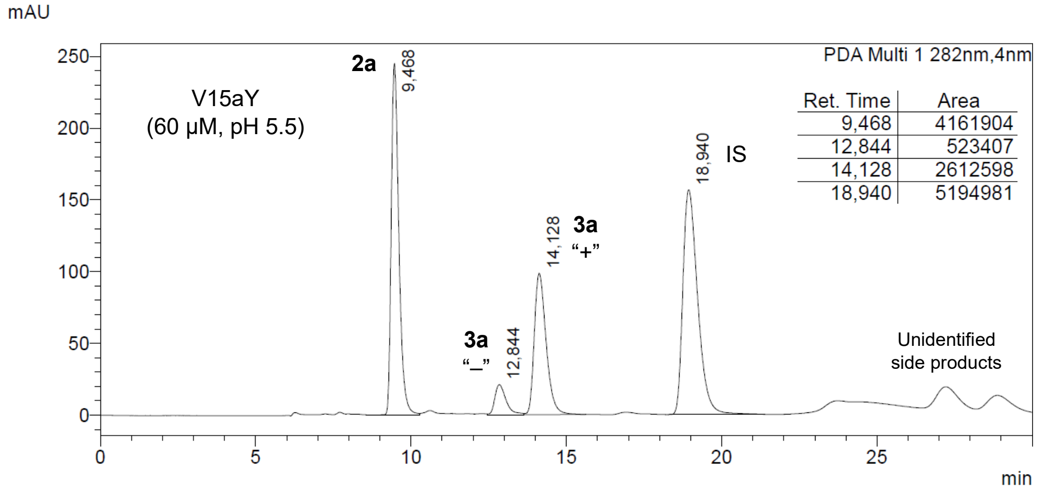

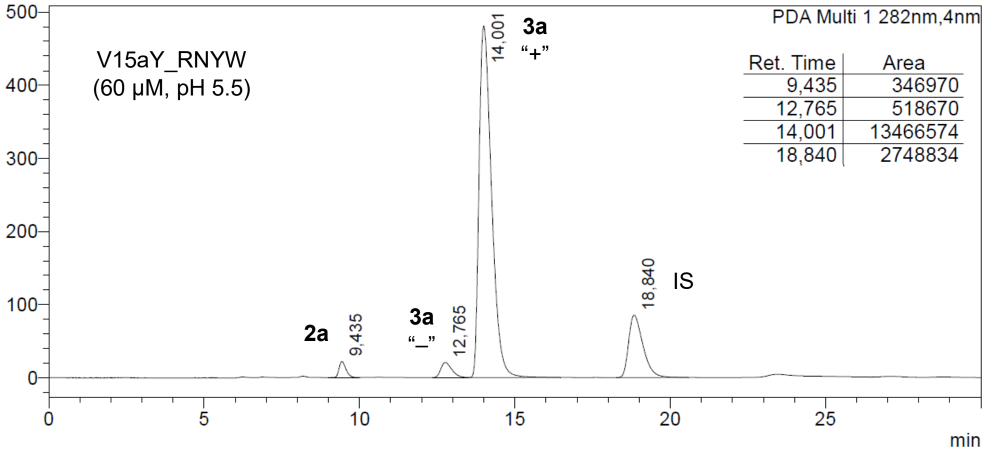

<u>SFC – product **3a** (3-(2-methyl-1H-indol-3-yl)butan-1-ol)</u>

SFC Method: Chiralcel OJ-3, $scCO_2$ (A) and MeOH (B) as mobile phase, linear gradient from 3% B to 50% B in 4.5 min, followed by 50% B for 2 min (6.5 min total), column was re-equilibrated at 3% B for 1.5 min in between samples (not shown in SFC-traces), 1.8 mL/min, 10 µL injection, integrated at 282 nm.

3a "–": 2.7 min (λ_{max} 282 nm)
3a "+": 2.9 min (λ_{max} 282 nm)
IS: 3.5 min (λ_{max} 281 nm)
2a: 3.6 min (λ_{max} 269 nm)

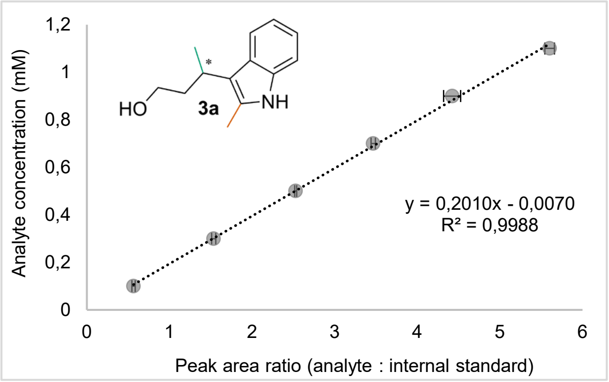

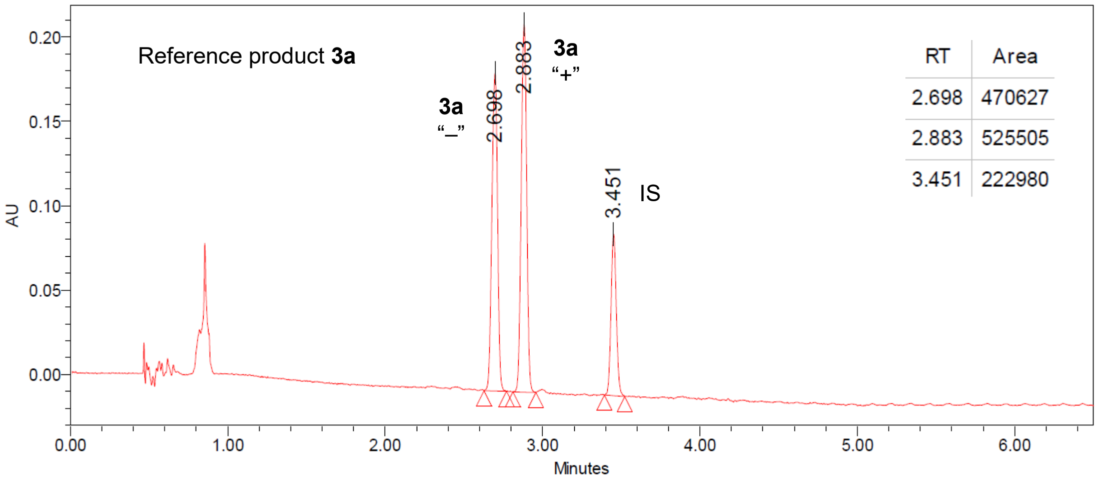

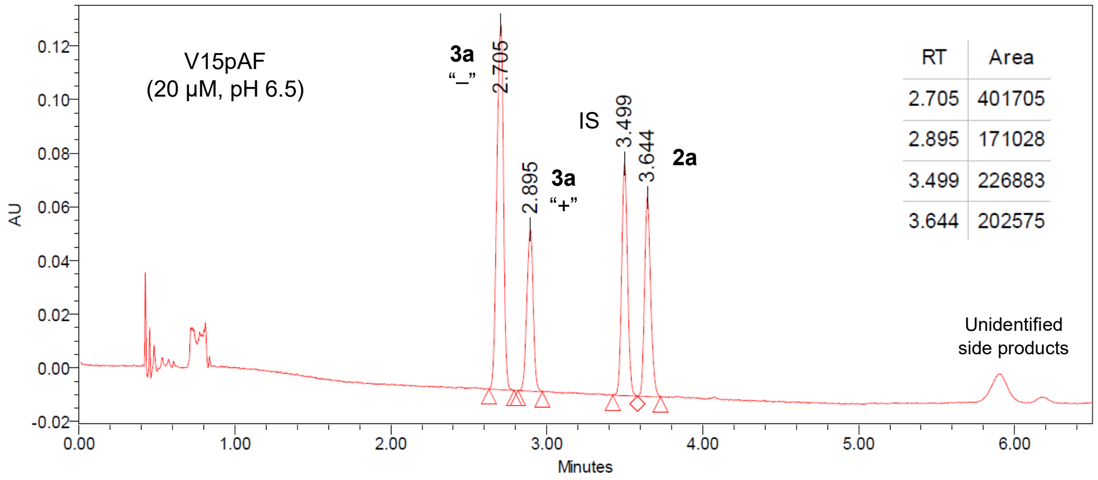
V15pAF
(20 μM, pH 6.5)
3a "–"
2.705
2.895
3a "+"
IS
3.499
3.644
2a
RT Area
2.705 401705
2.895 171028
3.499 226883
3.644 202575
Unidentified side products
AU
Minutes

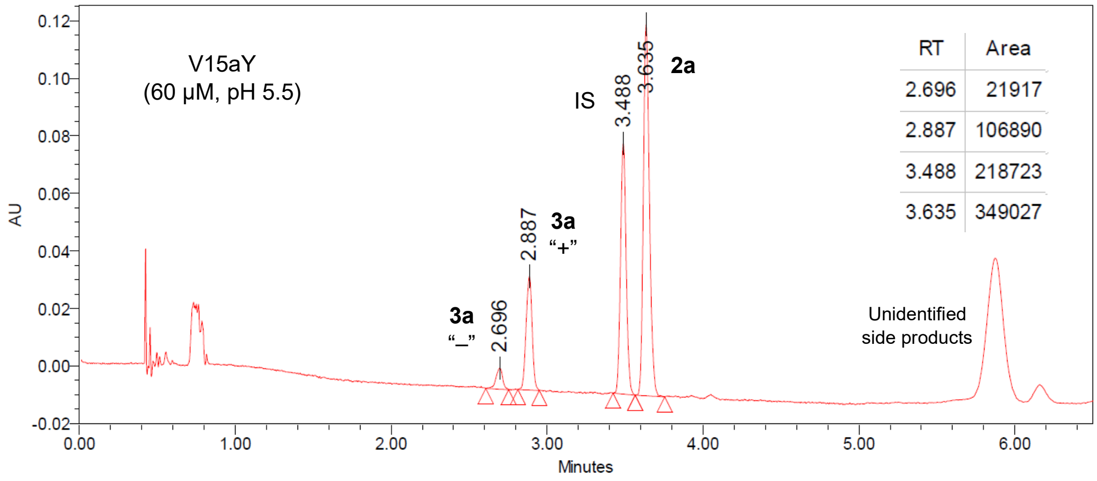
V15aY
(60 μM, pH 5.5)
3a "–"
2.696
2.887
3a "+"
IS
3.488
3.635
2a
RT Area
2.696 21917
2.887 106890
3.488 218723
3.635 349027
Unidentified side products
AU
Minutes

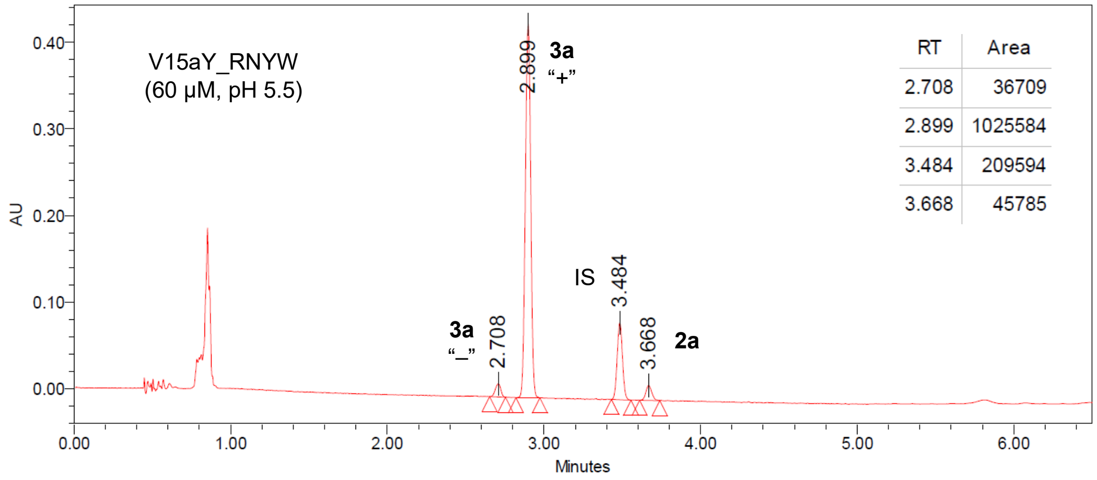
V15aY_RNYW
(60 μM, pH 5.5)
2.899
3a "+"
3a "–"
2.708
IS
3.484
3.668
2a
RT Area
2.708 36709
2.899 1025584
3.484 209594
3.668 45785
AU
Minutes

<u>HPLC – Product **3b** (3-(2-methyl-1H-indol-3-yl)hexan-1-ol)</u>

HPLC method: Luxcellulose-3 (Chiralcel OJ-H), 80:20 *n*-heptane:iPrOH, 1 mL/min, 25 min, 20 µL injection, integrated at 282 nm.

3b "–": 5.9 min (λ_{max} 282 nm)
3b "+": 6.7 min (λ_{max} 282 nm)
2a: 13.4 min (λ_{max} 268 nm)
IS: 16.2 min (λ_{max} 281 nm)

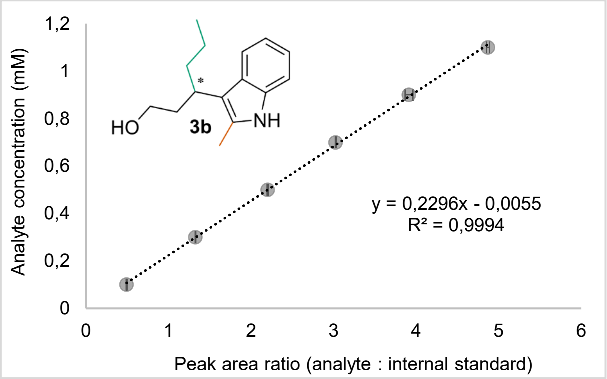

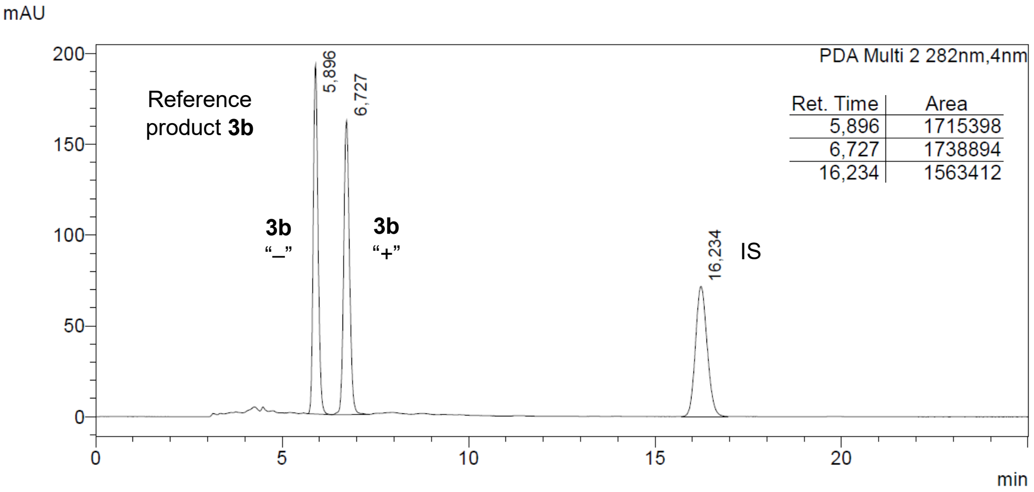

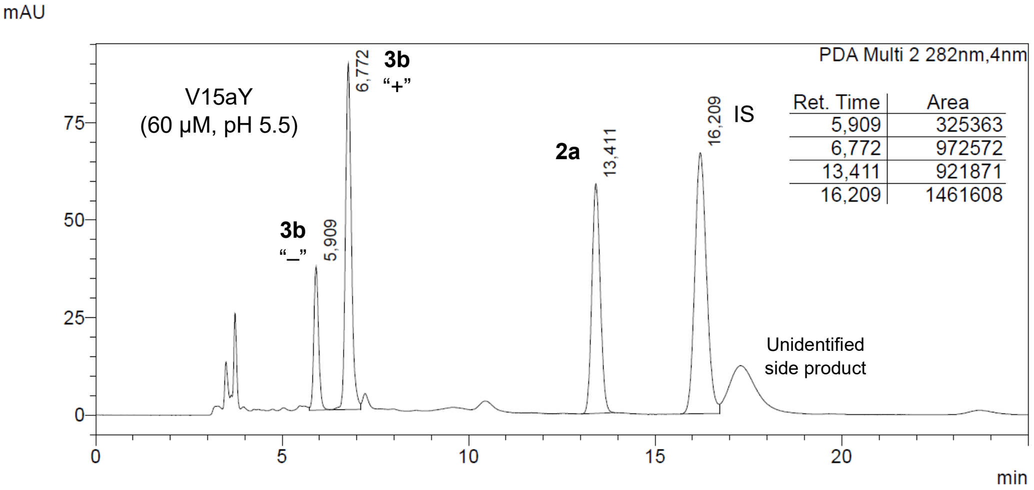

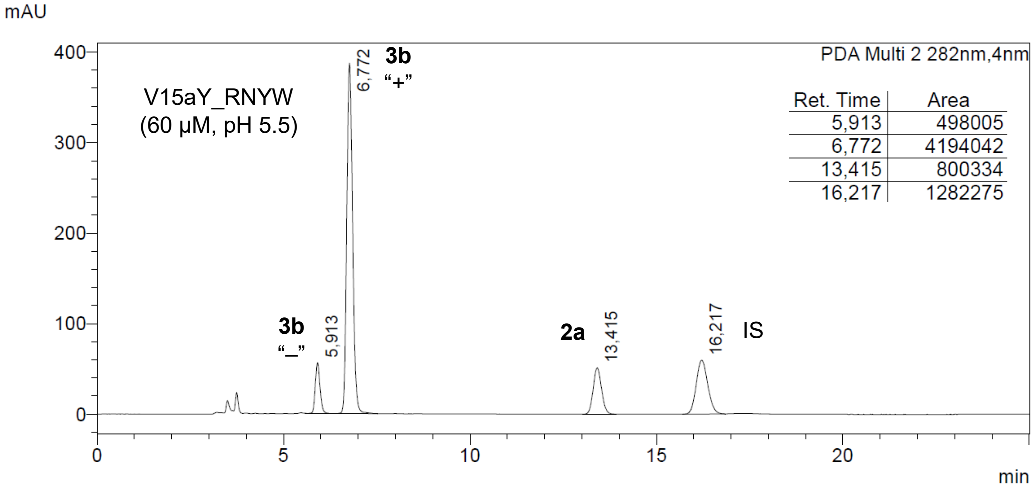

HPLC – Product **3c** (4-methyl-3-(2-methyl-1H-indol-3-yl)pentan-1-ol)

HPLC method: Chiralpak AS-H, 90:10 *n*-heptane:iPrOH, 0.5 mL/min, 40 min, 20 µL injection, integrated at 282 nm.

2a: 9.4 min (λ_{max} 268 nm)
3c "–": 11.1 min (λ_{max} 282 nm)
3c "+": 12.8 min (λ_{max} 282 nm)
IS: 18.9 min (λ_{max} 281 nm)

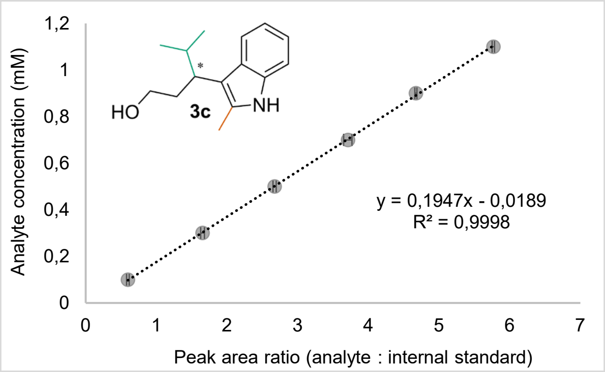

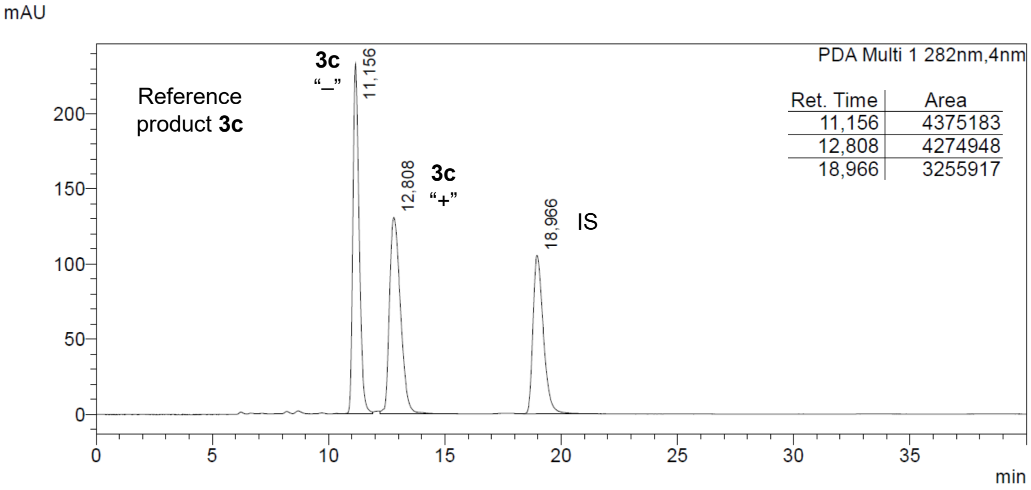

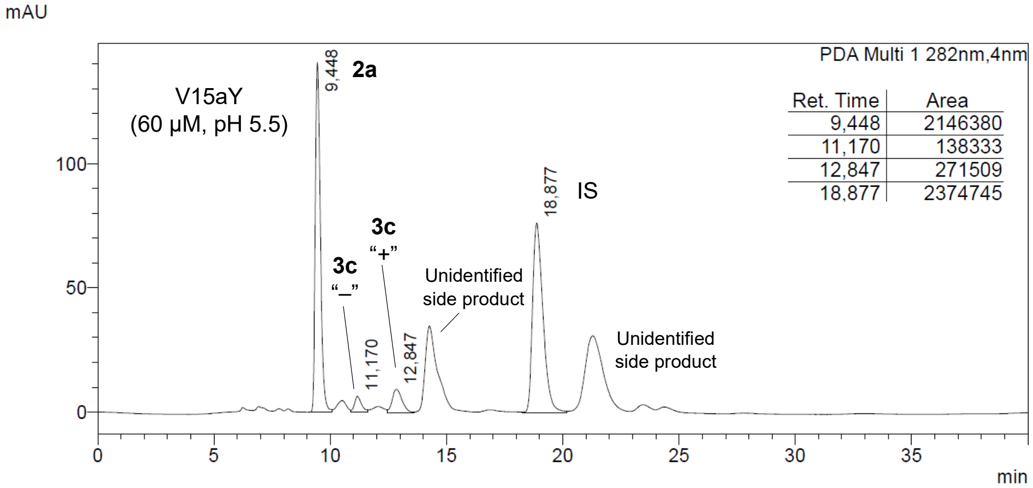

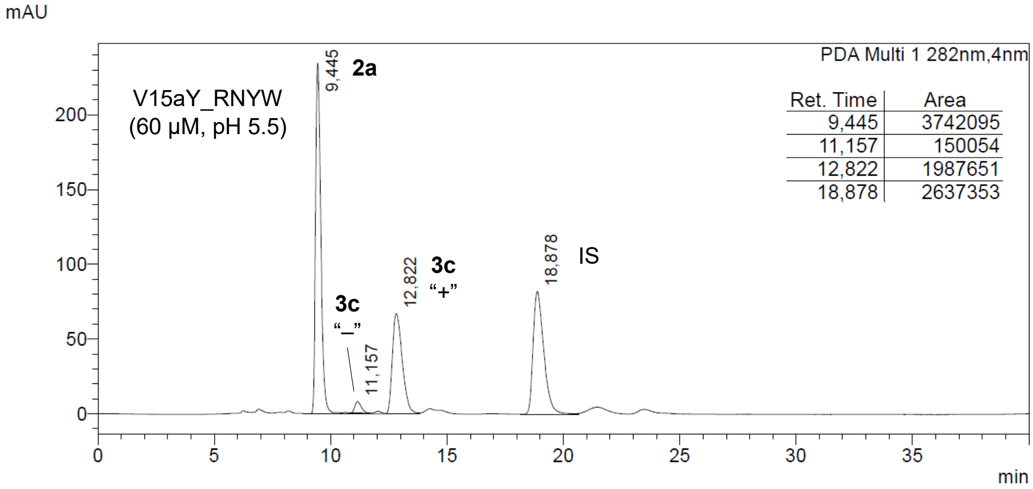

HPLC – Product **3d** (3-(2-methyl-1H-indol-3-yl)-3-phenylpropan-1-ol)

HPLC method: Luxcellulose-1 (Chiralcel OD-H), 85:15 *n*-heptane:iPrOH, 1 mL/min, 40 min, 20 µL injection, integrated at 282 nm

2a: 8.2 min (λ_{max} 268 nm)
1d: 9.3 min (λ_{max} 235, 260 nm)
IS: 20.4-20.7 min (λ_{max} 281 nm)
3d "–": 25.8-26.0 min (λ_{max} 282 nm)
3d "+": 28.3-28.9 min (λ_{max} 282 nm)

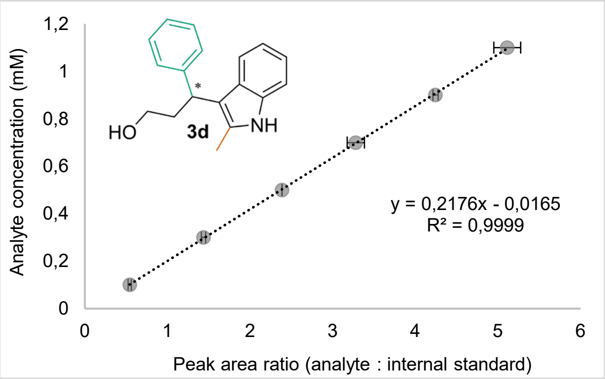

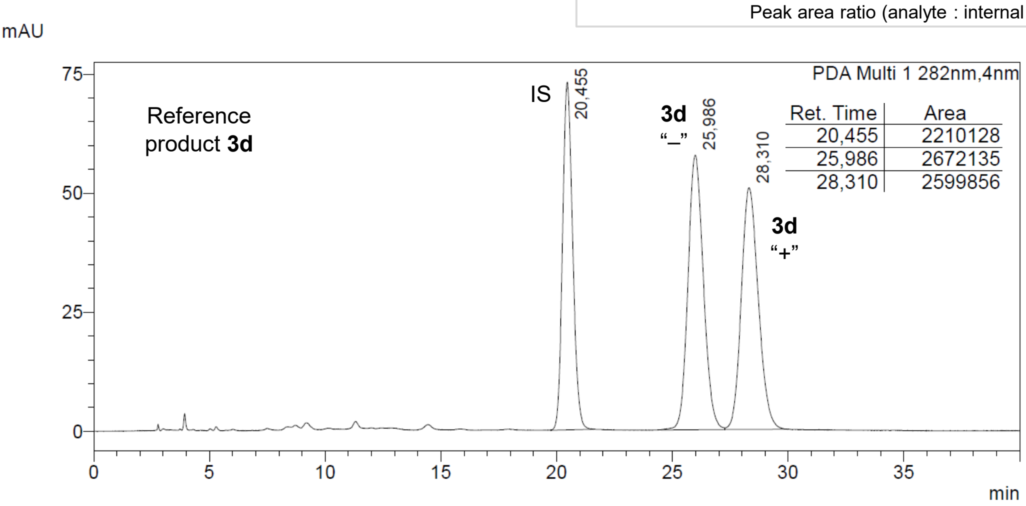

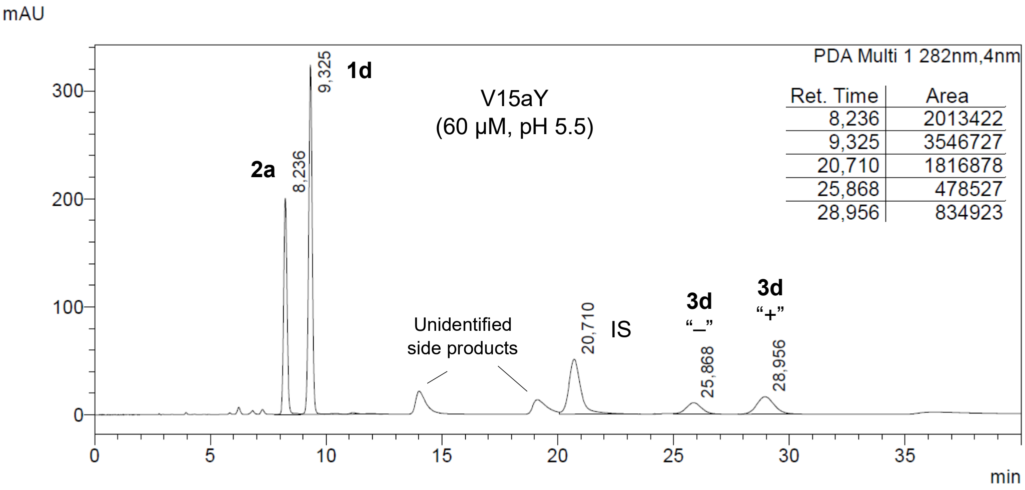

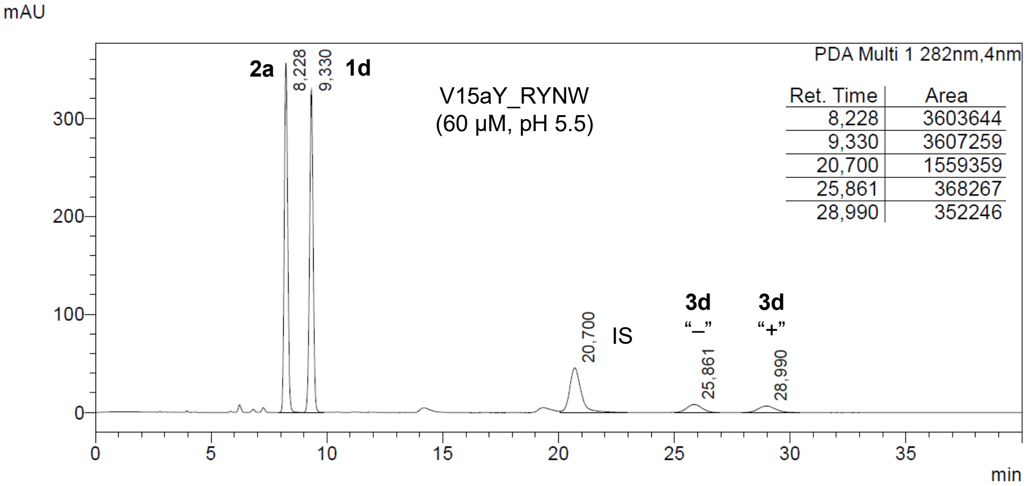

HPLC – Product **3e** (3-(1H-indol-3-yl)butan-1-ol)

HPLC method: Luxcellulose-1 (Chiralcel OD-H), 85:15 *n*-heptane:iPrOH, 1 mL/min, 40 min, 20 µL injection, integrated at 282 nm. Absolute configuration of products is assigned by comparison of order of elution with the literature and previous work.[5,19]

2e: 10.0 min (λ_{max} 268 nm)
3e (*R*): 14.4 min (λ_{max} 282 nm)
3e (*S*): 16.0 min (λ_{max} 282 nm)
IS: 20.7 min (λ_{max} 281 nm)

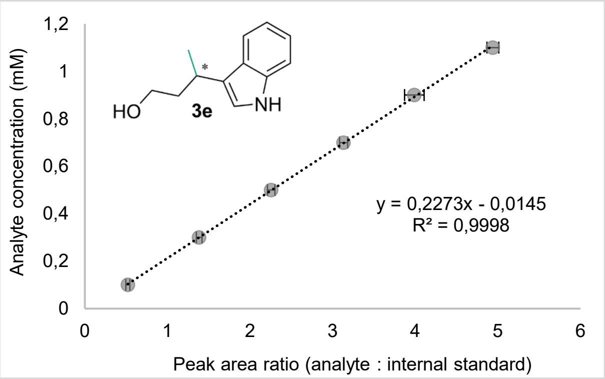

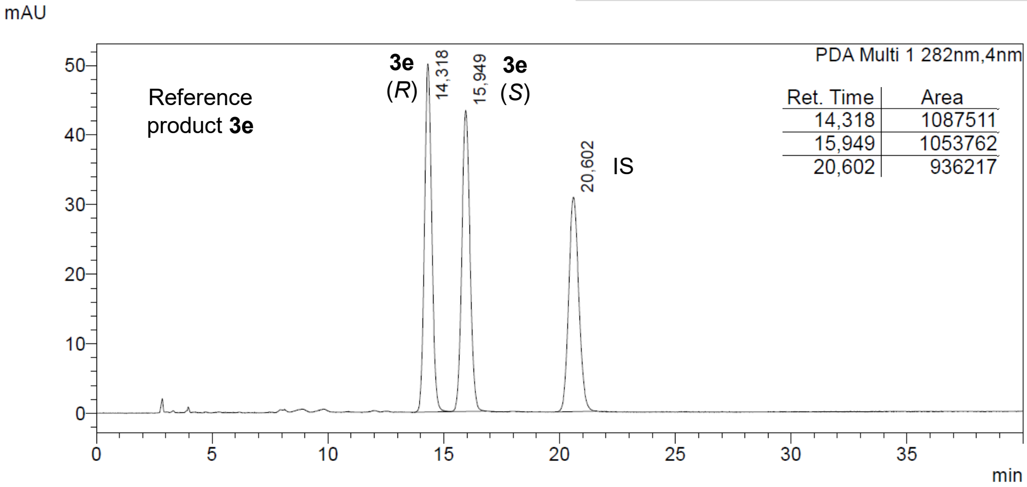

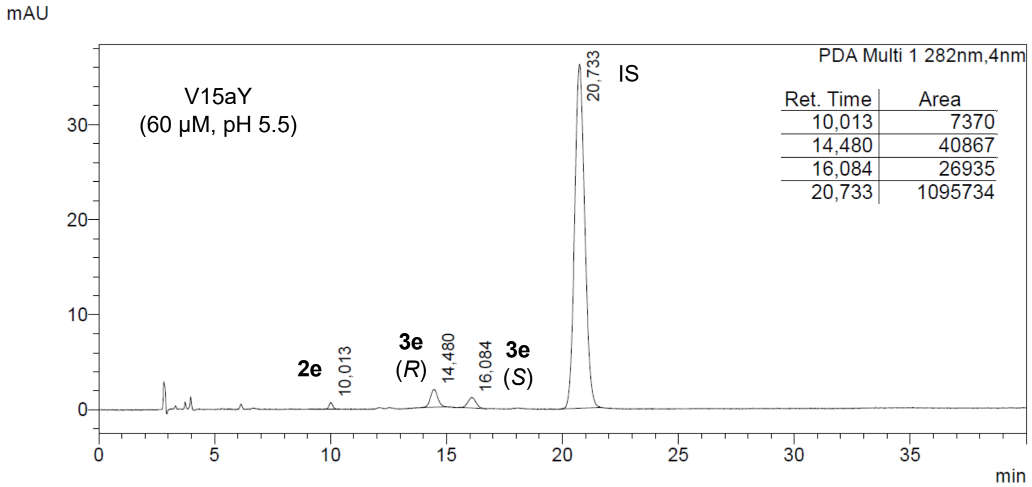

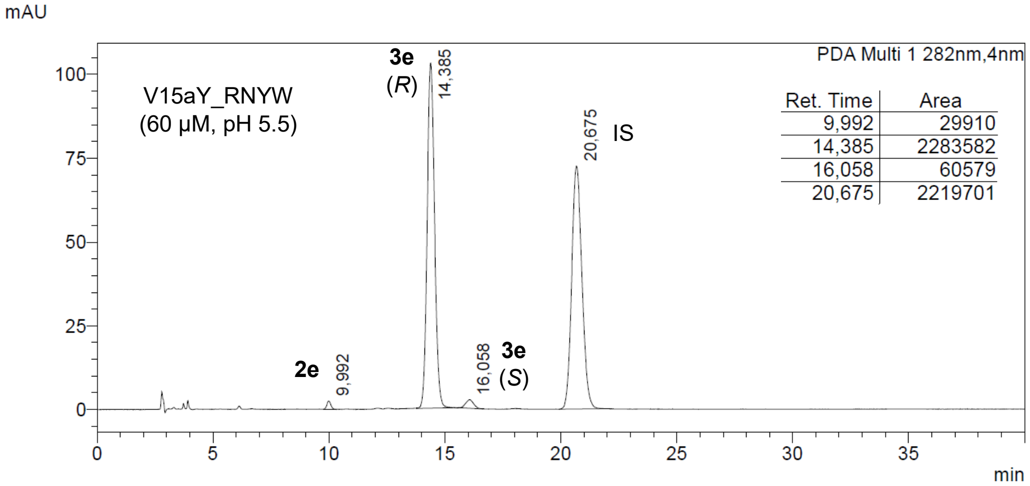

HPLC #2 – Product **3e** (3-(1H-indol-3-yl)butan-1-ol)

HPLC method #2: Luxcellulose-1 (Chiralcel OD-H), 90:10 *n*-hexane:EtOH, 1 mL/min, 40 min, 20 µL injection, integrated at 282 nm. Absolute configuration of products is assigned by comparison of order of elution with the literature and previous work.[5,19]

2e: 9.8 min (λ_{max} 268 nm)
3e (*R*): 16.1 min (λ_{max} 282 nm)
3e (*S*): 17.8 min (λ_{max} 282 nm)
IS: 23.2 min (λ_{max} 281 nm)

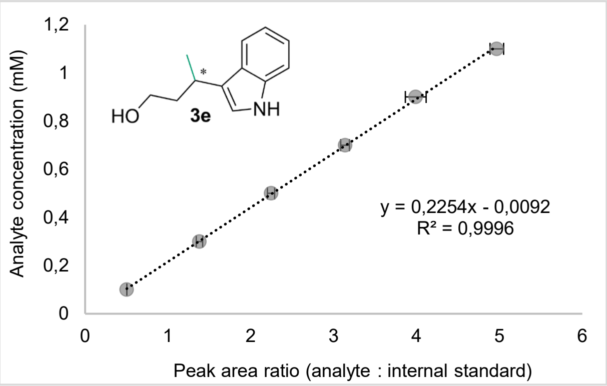

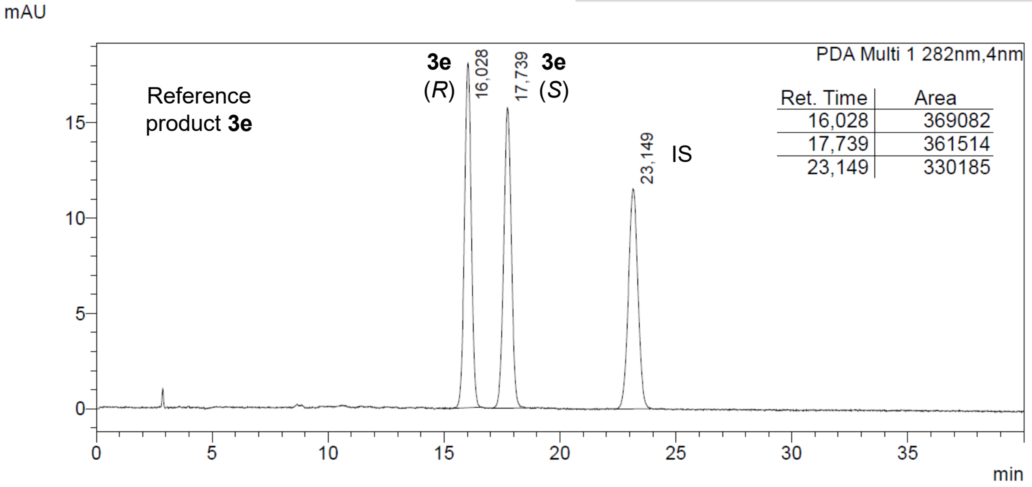

Ret. Time	Area
16,028	369082
17,739	361514
23,149	330185

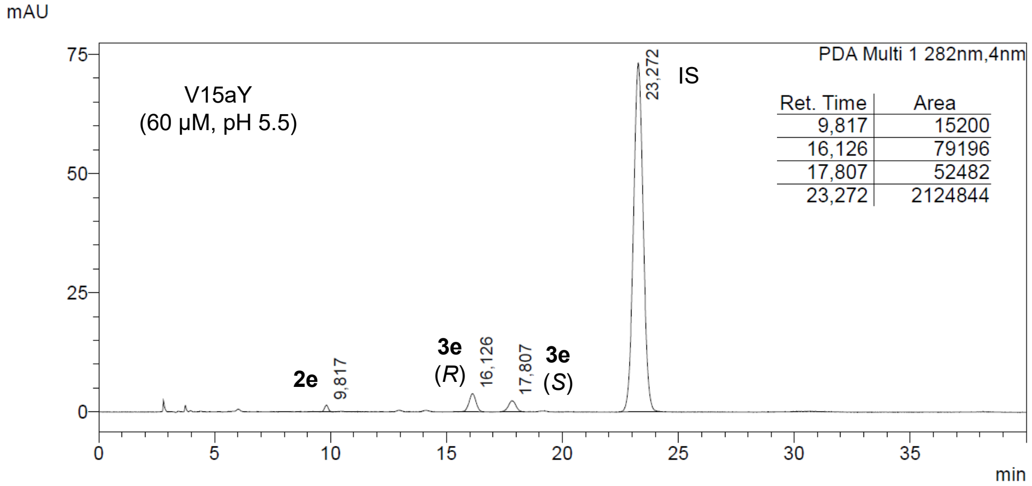

Ret. Time	Area
9,817	15200
16,126	79196
17,807	52482
23,272	2124844

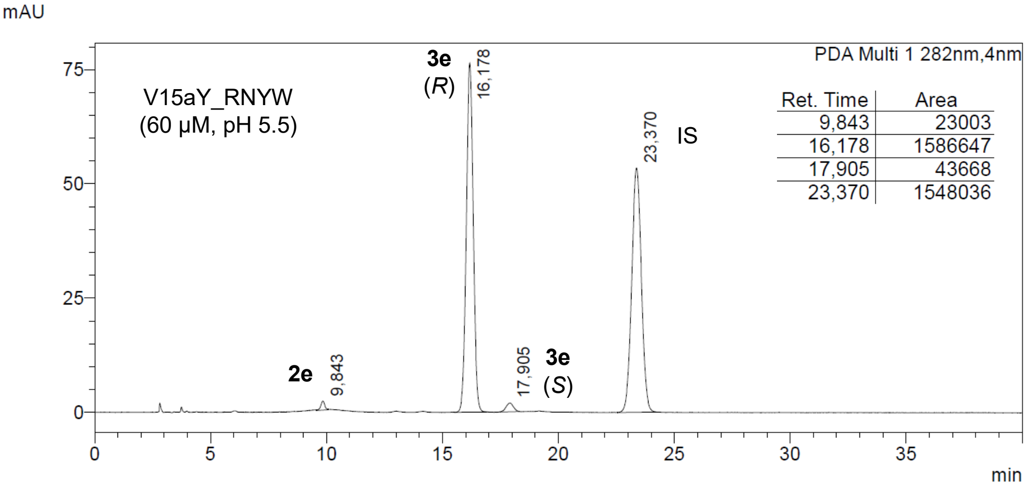

Ret. Time	Area
9,843	23003
16,178	1586647
17,905	43668
23,370	1548036

HPLC – Product **3f** (3-(5-methyl-1H-indol-3-yl)butan-1-ol)

HPLC method: Luxcellulose-3 (Chiralcel OJ-H), 80:20 *n*-heptane:iPrOH, 0.5 mL/min, 45 min, 20 µL injection, integrated at 287 nm

3f "+": 18.0 min (λ_{max} 287 nm)
3f "–": 23.0 min (λ_{max} 287 nm)
2f: 28.7 min (λ_{max} 268 nm)
IS: 32.4 min (λ_{max} 281 nm)

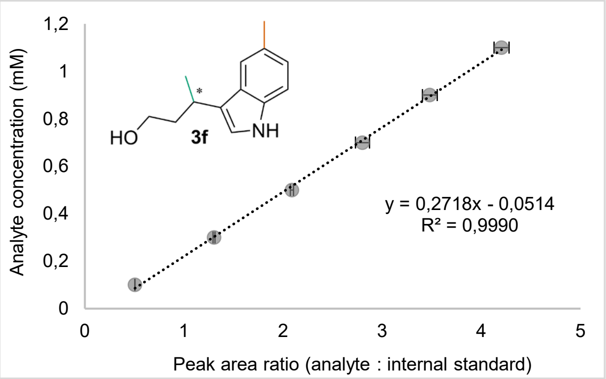

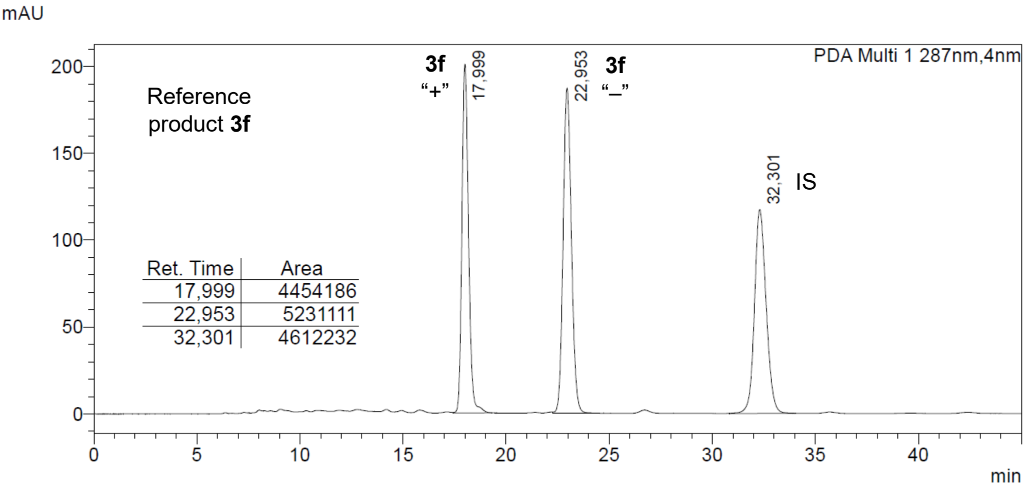

Ret. Time	Area
17,999	4454186
22,953	5231111
32,301	4612232

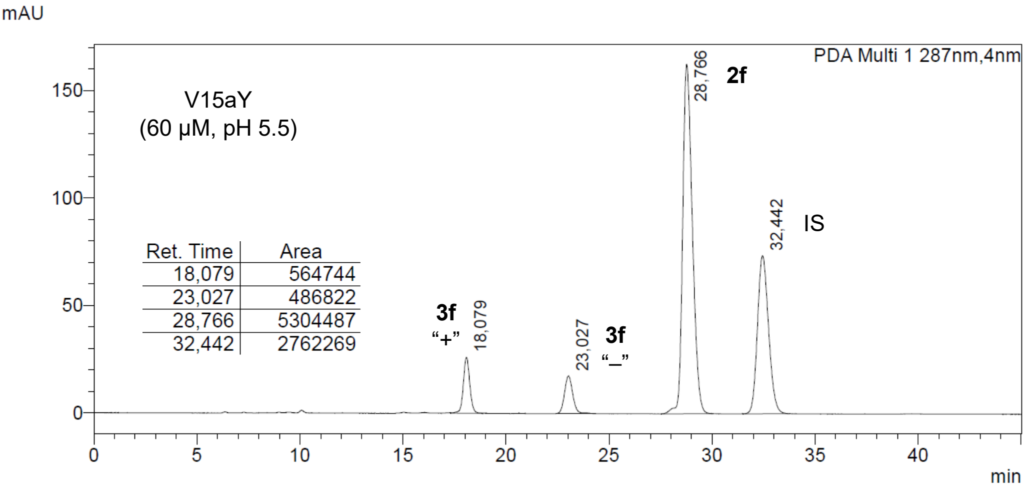

Ret. Time	Area
18,079	564744
23,027	486822
28,766	5304487
32,442	2762269

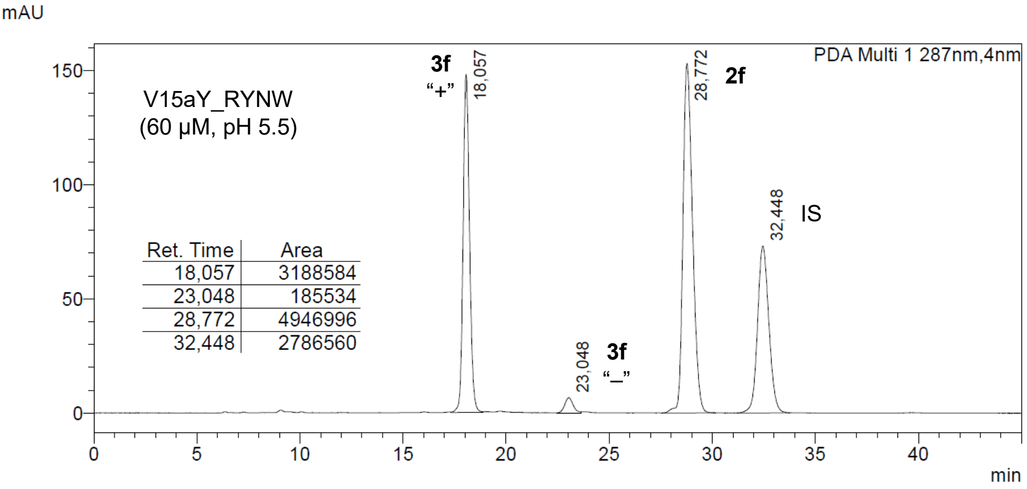

Ret. Time	Area
18,057	3188584
23,048	185534
28,772	4946996
32,448	2786560

HPLC – Product **3g** (3-(5-methoxy-1H-indol-3-yl)butan-1-ol)

HPLC method: Luxcellulose-3 (Chiralcel OJ-H), 85:15 *n*-heptane:iPrOH, 1 mL/min, 40 min, 20 µL injection, integrated at 278 nm.

3g "–": 16.6 min (λ_{max} 278 nm)
3g "+": 18.6 min (λ_{max} 278 nm)
IS: 25.1 min (λ_{max} 281 nm)
2g: 29.2 min (λ_{max} 268 nm)

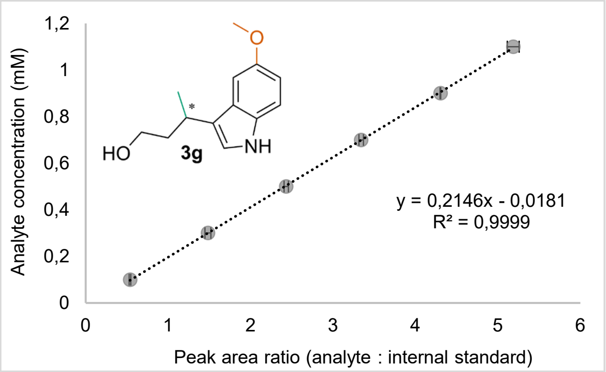

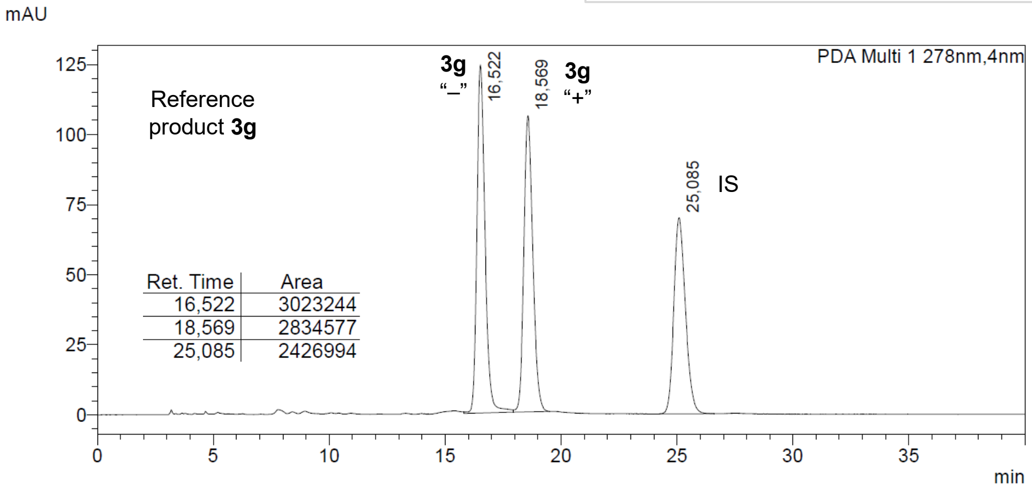

Ret. Time	Area
16,522	3023244
18,569	2834577
25,085	2426994

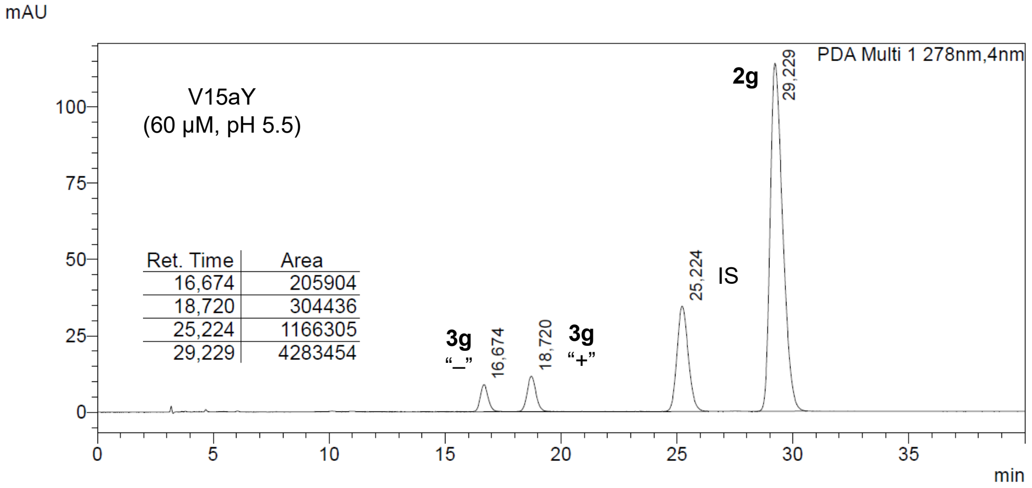

Ret. Time	Area
16,674	205904
18,720	304436
25,224	1166305
29,229	4283454

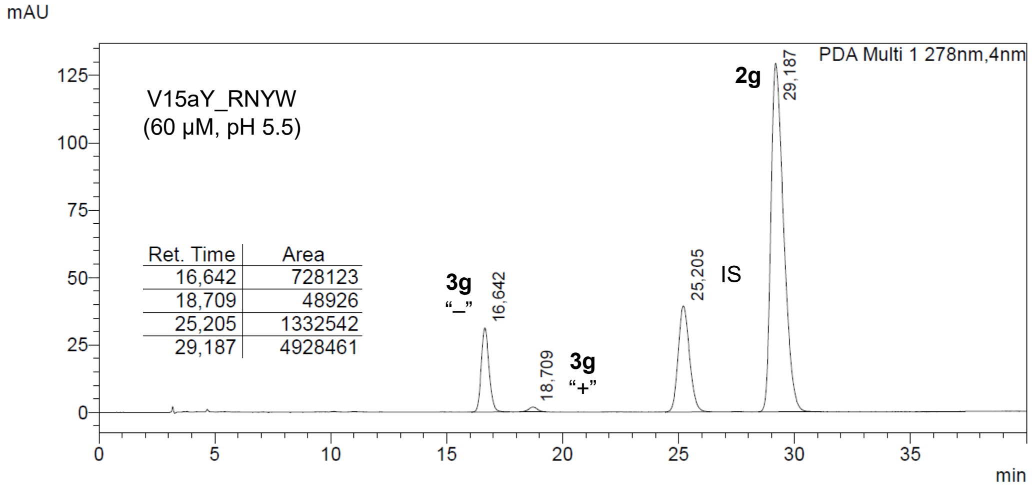

Ret. Time	Area
16,642	728123
18,709	48926
25,205	1332542
29,187	4928461

HPLC – Product **3h** (3-(5-bromo-1H-indol-3-yl)butan-1-ol)

HPLC method: Luxcellulose-3 (Chiralcel OJ-H), 85:15 *n*-heptane:iPrOH, 1 mL/min, 40 min, 20 µL injection, integrated at 291 nm.

3h "+": 9.9 min (λ_{max} 291 nm)
3h "–": 11.2 min (λ_{max} 291 nm)
2h: 12.5 min (λ_{max} 273 nm)
IS: 25.1 min (λ_{max} 281 nm)

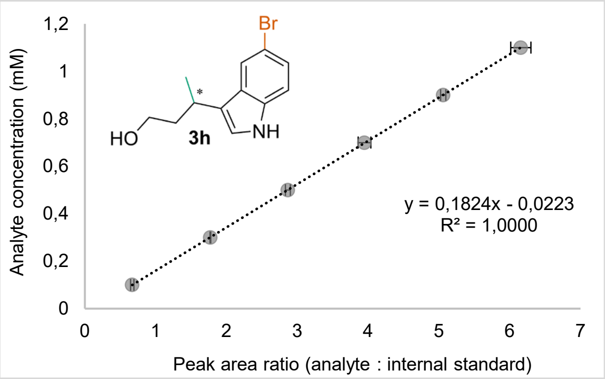

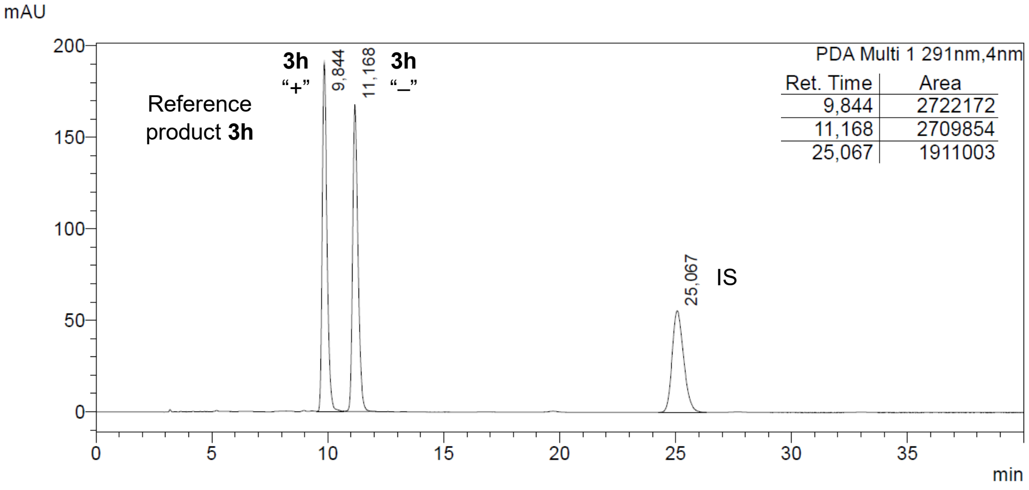

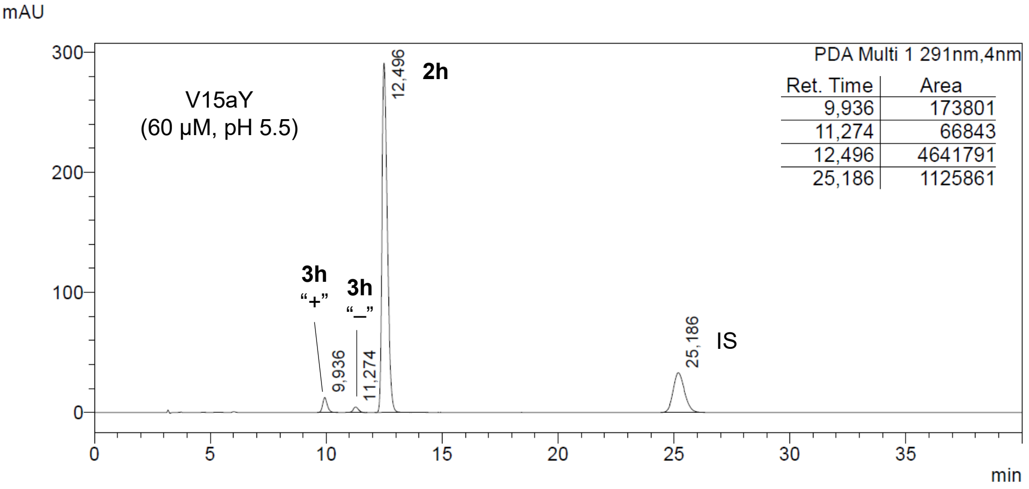

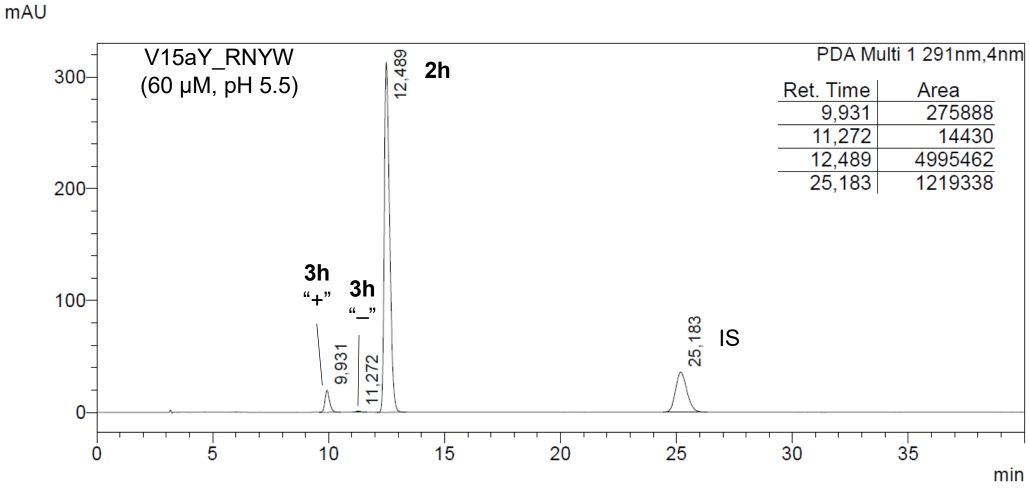

12. References

1 C. Mayer, C. Dulson, E. Reddem, A. W. H. Thunnissen and G. Roelfes, *Angew. Chem. Int. Ed.*, 2019, **58**, 2083–2087.

2 S. Zhang and H. Ai, *Nat. Chem. Biol.*, 2020, **16**, 1434–1439.

3 M. Amiram, A. D. Haimovich, C. Fan, Y.-S. Wang, H.-R. Aerni, I. Ntai, D. W. Moonan, N. J. Ma, A. J. Rovner, S. H. Hong, N. L. Kelleher, A. L. Goodman, M. C. Jewett, D. Söll, J. Rinehart and F. J. Isaacs, *Nat. Biotechnol.*, 2015, **33**, 1272–1279.

4 I. Drienovská, C. Mayer, C. Dulson and G. Roelfes, *Nat. Chem.*, 2018, **10**, 946–952.

5 R. B. Leveson-Gower, Z. Zhou, I. Drienovská and G. Roelfes, *ACS Catal.*, 2021, **11**, 6763–6770.

6 H. Im, *Bio-101*, 2011, **1**, e143, DOI: 10.21769/BioProtoc.143.

7 Z. Zhang and A. G. Marshall, *J. Am. Soc. Mass Spectrom.*, 1998, **9**, 225–233.

8 G. Winter, C. M. C. Lobley and S. M. Prince, *Acta Crystallogr. D Biol. Crystallogr.*, 2013, **69**, 1260–1273.

9 G. Winter, D. G. Waterman, J. M. Parkhurst, A. S. Brewster, R. J. Gildea, M. Gerstel, L. Fuentes-Montero, M. Vollmar, T. Michels-Clark, I. D. Young, N. K. Sauter and G. Evans, *Acta Crystallogr. Sect. Struct. Biol.*, 2018, **74**, 85–97.

10 W. Kabsch, *Acta Crystallogr. D Biol. Crystallogr.*, 2010, **66**, 125–132.

11 P. R. Evans and G. N. Murshudov, *Acta Crystallogr. D Biol. Crystallogr.*, 2013, **69**, 1204–1214.

12 J. Agirre, M. Atanasova, H. Bagdonas, C. B. Ballard, A. Baslé, J. Beilsten-Edmands, R. J. Borges, D. G. Brown, J. J. Burgos-Mármol, J. M. Berrisford, P. S. Bond, I. Caballero, L. Catapano, G. Chojnowski, A. G. Cook, K. D. Cowtan, T. I. Croll, J. É. Debreczeni, N. E. Devenish, E. J. Dodson, T. R. Drevon, P. Emsley, G. Evans, P. R. Evans, M. Fando, J. Foadi, L. Fuentes-Montero, E. F. Garman, M. Gerstel, R. J. Gildea, K. Hatti, M. L. Hekkelman, P. Heuser, S. W. Hoh, M. A. Hough, H. T. Jenkins, E. Jiménez, R. P. Joosten, R. M. Keegan, N. Keep, E. B. Krissinel, P. Kolenko, O. Kovalevskiy, V. S. Lamzin, D. M. Lawson, A. A. Lebedev, A. G. W. Leslie, B. Lohkamp, F. Long, M. Malý, A. J. McCoy, S. J. McNicholas, A. Medina, C. Millán, J. W. Murray, G. N. Murshudov, R. A. Nicholls, M. E. M. Noble, R. Oeffner, N. S. Pannu, J. M. Parkhurst, N. Pearce, J. Pereira, A. Perrakis, H. R. Powell, R. J. Read, D. J. Rigden, W. Rochira, M. Sammito, F. Sánchez Rodríguez, G. M. Sheldrick, K. L. Shelley, F. Simkovic, A. J. Simpkin, P. Skubak, E. Sobolev, R. A. Steiner, K. Stevenson, I. Tews, J. M. H. Thomas, A. Thorn, J. T. Valls, V. Uski, I. Usón, A. Vagin, S. Velankar, M. Vollmar, H. Walden, D. Waterman, K. S. Wilson, M. D. Winn, G. Winter, M. Wojdyr and K. Yamashita, *Acta Crystallogr. Sect. Struct. Biol.*, 2023, **79**, 449–461.

13 A. J. McCoy, R. W. Grosse-Kunstleve, P. D. Adams, M. D. Winn, L. C. Storoni and R. J. Read, *J. Appl. Crystallogr.*, 2007, **40**, 658–674.

14 P. Emsley, B. Lohkamp, W. G. Scott and K. Cowtan, *Acta Crystallogr. D Biol. Crystallogr.*, 2010, **66**, 486–501.

15 G. N. Murshudov, P. Skubák, A. A. Lebedev, N. S. Pannu, R. A. Steiner, R. A. Nicholls, M. D. Winn, F. Long and A. A. Vagin, *Acta Crystallogr. D Biol. Crystallogr.*, 2011, **67**, 355–367.

16 C. J. Williams, J. J. Headd, N. W. Moriarty, M. G. Prisant, L. L. Videau, L. N. Deis, V. Verma, D. A. Keedy, B. J. Hintze, V. B. Chen, S. Jain, S. M. Lewis, W. B. Arendall III, J. Snoeyink, P. D. Adams, S. C. Lovell, J. S. Richardson and D. C. Richardson, *Protein Sci.*, 2018, **27**, 293–315.

17 D. Carmona, M. P. Lamata, A. Sánchez, F. Viguri and L. A. Oro, *Tetrahedron Asymmetry*, 2011, **22**, 893–906.

18 X. Liang, S. Li and W. Su, *Tetrahedron Lett.*, 2012, **53**, 289–291.

19 J. F. Austin and D. W. C. MacMillan, *J. Am. Chem. Soc.*, 2002, **124**, 1172–1173.

20 Deepa, Mohd. J. Aalam, P. Kumar and S. Singh, *Tetrahedron Lett.*, 2023, **116**, 154343.
